# Supplementary material for: CAPRI enables comparison of evolutionarily conserved RNA interacting regions
Source: Nat Commun. 2019 Jun 18;10:2682. doi: 10.1038/s41467-019-10585-3 (PMC6581911; doi:10.1038/s41467-019-10585-3)
Supplement: Supplementary file 1 — Supplementary Information [file 41467_2019_10585_MOESM1_ESM.pdf]

CAPRI enables comparison of evolutionarily conserved RNA interacting regions

Panhale A et al. 2019

Supplementary Notes,

Supplementary Figures and

Supplementary References

Supplementary Note 1: Comparison of UV and FA crosslinking

UV irradiation at 254 nm is a robust method to create covalent “zero length” crosslinks between nucleotides and neighbouring amino acid residues. Since the reaction is initiated by excitation of nucleobases in RNA the covalent crosslinks are formed specifically between RNA and protein and not intra- or inter-protein. Although, inter-peptide crosslinks mediated by UV have been reported they require either extensive UV irradiation for 30 minutes[1] or with laser based UV irradiation[2]. Also, the rapid kinetics of the reaction make the interaction specific to neighbouring amino acids[3–5]. The covalent bond between nucleotides and proteins is stable and can withstand highly stringent detergent and temperature washing conditions[6,7]. Thus UV irradiation is highly suited to study RNA protein interactions. However, UV has a bias towards single-stranded RNA regions and crosslinks mediated via the nucleobase uridine[7–9].

Formaldehyde (FA) is the shortest bifunctional chemical crosslinker and can undergo diverse chemical reactions[10]. It can quickly diffuse through living cells thereby freezing biomolecular interactions and preventing intercompartment mixing[11]. FA can form intra- and intermolecular covalent bonds between nucleic acids and proteins. Similar to UV-mediated crosslinks, these crosslinks can withstand stringent detergent washes, however the covalent bonds can be reversed with heat. Hence, FA has been extensively used to study chromatin/DNA-protein interactions (e.g. ChIP, Bio-TAP-XL[12], PiCh[13], RIME[14], CAPTURE[15] and reviewed in Hoffman, E.A. et al.[16]). It has also been used to study protein complexes[17–19]. FA has also been recently used to investigate RNA-protein interactions of specific RNAs using biotinylated antisense probes as part of the CHIRP-MS[20], dCHIRP[21] and CHART[22] protocols. It has also been used in protein-centric approaches to interrogate short range RNA-protein interactions in the RIPiT-Seq technique[23]. Under the crosslinking conditions and short incubation times (0.1% fresh monomeric FA incubated for 10 min at room temperature) used in the present study, most of the reactions would be driven through immonium cations and largely limited to lysine, tryptophan side chains, and the amino termini of proteins[10]. We additionally ensured that FA crosslinking does not create extensive networks of crosslinks by optimising for its yields to be similar to UV irradiation.

Supplementary Note 2: Adjacent versus crosslinked peptides

Crosslinked peptides emanating from a single location in close proximity to RNA are more diverse than corresponding adjacent peptides(Supplementary Fig. 5a). This diversity arises from the various combinations of amino acids and nucleotides which can occur at the crosslinking

site and the variable number of nucleotides left behind after RNase digestion. Each of these heteroconjugate peptides are detected separately in mass spectrometric analysis. The adjacent peptides on the other hand are homogeneous and thus more abundant and easier to detect.

Supplementary Note 3: Adjacent peptide extension

We extended adjacent peptides to the neighbouring LysC digestion sites. The resulting ADJ-peptides encompass the predicted crosslinking sites. In many cases a single LysC ADJ-peptide may be composed of more than one internal tryptic adjacent peptide. Adjacent peptides add up to the coverage shown for each of the ADJ-peptides in the protein profiles shown in the manuscript.

Supplementary Note 4: Crosslinked peptide analysis

RNA-protein interactions can be studied by covalently fixing them through UV irradiation (wavelength 254 nm). UV selectively excites ribonucleobases, which preferentially form covalent adducts with hydrophobic and aromatic amino acids (W, Y, F, H, M, I, L, V, G, A, C) (reviewed in Schmidt et al.[24]). RNA-peptide heteroconjugates suitable for LC-MS are generated by trimming the RNA to the length of one to maximally three ribonucleotides using nucleases and converting the proteins into peptides by using specific endoproteinases like trypsin.

The ionization efficiency of heteroconjugates is usually decreased by the presence of the phosphodiester backbone that partially remains negatively charged (pKa values of non-bridging oxygen atoms < 1.5) at the pH value (2-3) of the LC-MS buffer systems in use. Moreover, the presence of the highly polar ribose moiety can potentially lead to unfavourable chromatographic behaviour (impaired retention, peak shape distortion) on C18 reversed phase columns. As a consequence, identification of crosslinked peptides requires much higher sample amounts compared to standard MS-based proteomics experiments (similar to phosphoproteomics). To overcome this, enrichment strategies harnessing the physicochemical properties of the ribose phosphodiester backbone have been established[25].

Previous studies have demonstrated that the mass of a crosslinked RNA-peptide heteroconjugate is additive, i.e. the mass of the molecule equals the sum of the masses of its constituents, peptide and oligonucleotide[26]. The latter has been exploited in the context of homogeneously purified RNPs to screen MS1 spectra for the presence of precursor ion masses that can be matched by allowing a combinatorial permutation of ribonucleotide masses to be

added to known peptide ion masses[7]. In later studies, crosslinked peptide spectra were extracted from MS2 data by first identifying nucleobase marker ions, which result from (partial) collision-induced dissociation of the covalently bound ribonucleotides during MS2 fragmentation[8,25]. These marker ions are usually observed for three of the four RNA bases in the lower mass range of MS2 spectra, (e.g. m/z 112 for C (H^+), m/z 136 for A (H^+), m/z 152 for G (H^+) and the rarely observed m/z 113 for U (H^+)). Assuming a crosslink is exclusively established via the nucleobase, the presence of marker ions can only be observed for RNA-peptide heteroconjugate molecular ions, which harbor at least a dinucleotide modification. This knowledge was integrated into a computational tool (RNP^{XL}) embedded in the OpenMS environment[8], which enables semi-automated RNA-protein crosslink identification. RNP^{XL} executes a highly stringent and sophisticated multi-step filtering at the MS raw data level thereby trying to remove all MS2 spectra that are not likely to be RNA-peptide heteroconjugates. From the remaining MS1 precursor ion masses RNP^{XL} performs a permuted subtraction of the calculated exact masses of (user-defined) possible combinations of ribonucleotide adducts (e.g. UA, UG, UC, UU, UAA, UAG and so on). This returns a list of theoretically possible precursor ion masses exclusively considering the mass of the peptide moiety present in a crosslinked species. Finally, the latter are iteratively searched together with their corresponding MS2 spectra (OMSSA search engine) against a protein database in order to identify the peptide sequences of RNA-peptide heteroconjugates. Recently, a fast database search-based peptide identification tool, MSFragger, was described. MSFragger implements a novel fragment-ion indexing method to identify many RNA crosslinked peptides[27].

Supplementary Note 5: FA-based RBD capture

While UV is biased towards crosslinking of pyrimidines in single-stranded RNA (reviewed in Moore, K.S. and ‘t Hoen, P.A.C., 2019[9]), FA favours reactions with purines and is also able to target RNA double helices[28]. Moreover, UV can even photoactivate ribonucleobases buried in the core of protein structures. To the contrary, FA mainly freezes molecular interactions taking place at surface-exposed and accessible residues, at least under the conditions employed in the current work. Nevertheless, FA has some limitations for studying RNA-protein interactions, namely the additional formation of intra- and inter-protein crosslinks as well as the metastable nature of the covalent crosslink bonds.

Supplementary Note 6: Disordered amino acids in IDRs

Nucleic acid-binding proteins are known to contain the highest proportion of intrinsically disordered regions (IDRs) in the proteome. More than 30% of CAPRI-peptides mapped to IDRs, indicating a high preference for RNA-binding regions to be located in IDRs. The sequence composition of these regions is rich in disorder-promoting amino acids like proline (P), glycine (G), serine (S), glutamine (Q) and arginine (R) when compared to CAPRI peptides mapping to globular domains or to the proteome (Supplementary Fig. 16a,b). Interestingly, asparagine (N) was enriched in *Drosophila* IDRs(Supplementary Fig. 16a,c) whereas aromatic residues like tyrosine (Y) and tryptophan (W) were found in higher abundance in humans (Supplementary Fig. 16b,d). The disordered regions are composed of low sequence-complexity repeats and show high evolutionary sequence divergence[29]. We hence scanned these regions for enriched short regular expressions of amino acids using the DREME tool[30]. We found that the same triplets of amino acids including RGG, RSS, GFG and YGG were enriched in both species (Supplementary Fig. 16e,f). Interestingly, repeats of such triplets have been predicted to bind RNA[31–33]

Supplementary Figure 1

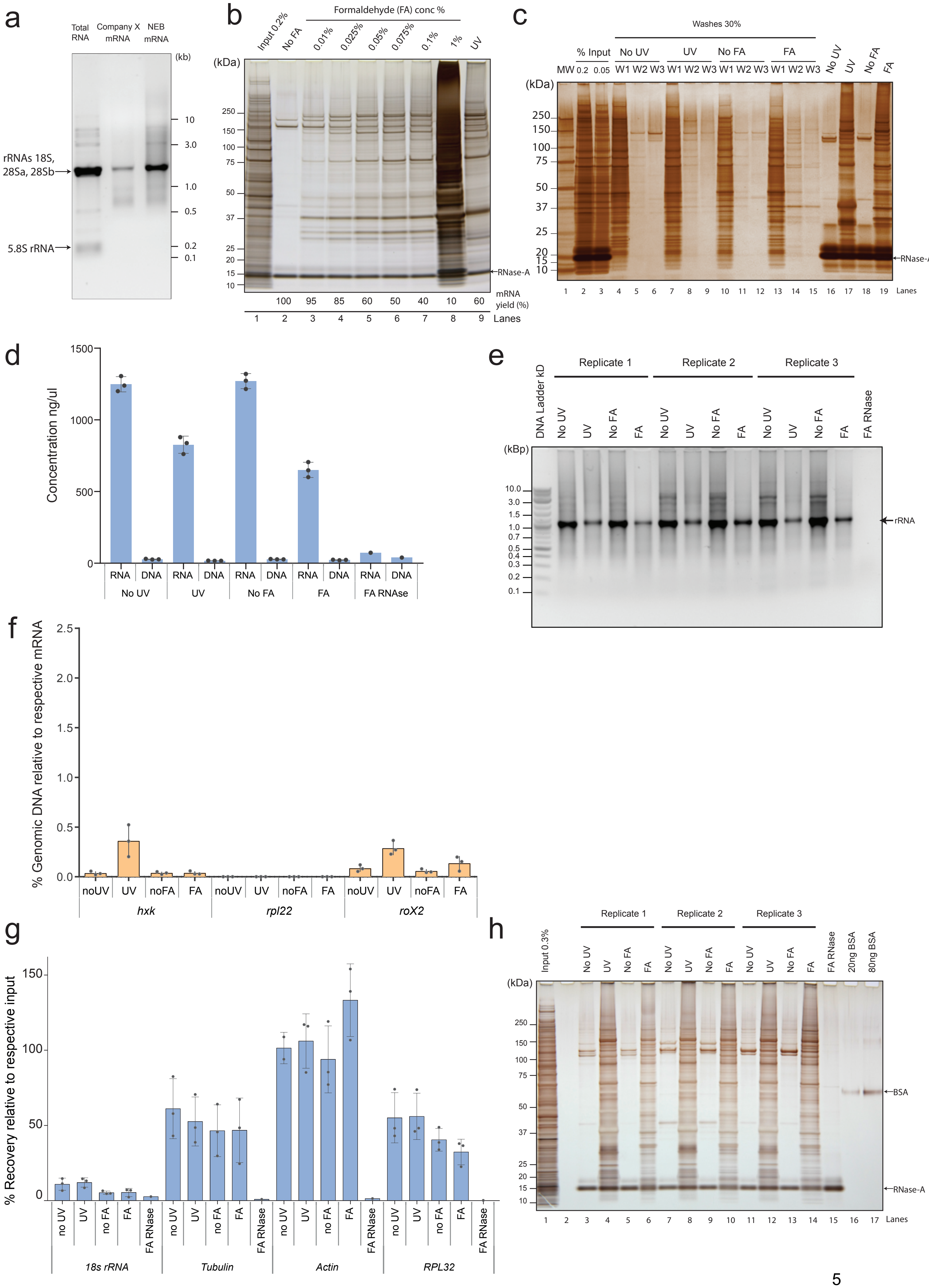

Supplementary Figure 1: RNA interactome capture in biological triplicates.

a. RNA profiles of total RNA and mRNA isolation by different oligo-dT-coupled beads.

b. Optimisation of formaldehyde crosslinking. Protein profiles analysed by silver staining after interactome capture with varying concentrations of formaldehyde (lanes 3-8). The RNA yields (rounded percent) relative to uncrosslinked samples are shown in the lane below the gel image. Proteins isolated from an equivalent number of UV crosslinked cells were loaded in lane 9 for comparison with FA yield.

c. Monitoring protein content through washing steps and elution in the UV and FA RNA interactome capture protocols. The first three washes (30% of each W1, W2, W3) have been included in lanes 4 to 15 to show the depletion of non-specifically interacting proteins in each of the conditions.

d. Nucleic acid yields (Qubit assay) No UV: sample derived from non-irradiated cells; UV: sample derived from UV-irradiated cells; no FA: sample without formaldehyde crosslinking; FA: formaldehyde-crosslinked sample; FA RNase: formaldehyde-crosslinked and RNase-pretreated sample. (Error bars represent standard deviation (s.d.) of three biological replicates. A single replicate of FA RNase sample was used.). Source data are provided as a Source Data file.

e. Analysis of RNA profiles from all biological replicates of interactome capture procedure by agarose gel electrophoresis (using equal volumes of eluate).

f. Fraction of genomic DNA (%) relative to the respective mRNA amounts based on qPCR quantification of the genes (mRNA and genomic DNA) *hxk, rpl22 and roX2*. Quantification was performed on equal volumes of reverse transcribed eluate (cDNA) and non-reverse transcribed eluate (genomic DNA). Error bars represent standard deviation (s.d.) of three biological replicates. Source data are provided as a Source Data file.

g. RNA quantitative PCR analysis was performed to estimate recovery and enrichment of mRNA (*tubulin, actin, RPL32*) as compared to 18s rRNA in each of the biological replicates. The recoveries of RNA were measured relative to the corresponding input samples. Error bars represent s.d. of three biological replicates, except the *actin* no UV sample: s.d. of two biological replicates. Source data are provided as a Source Data file.

h. Silver staining analysis of all three biological replicates (lanes 3-14).

Supplementary Figure 2

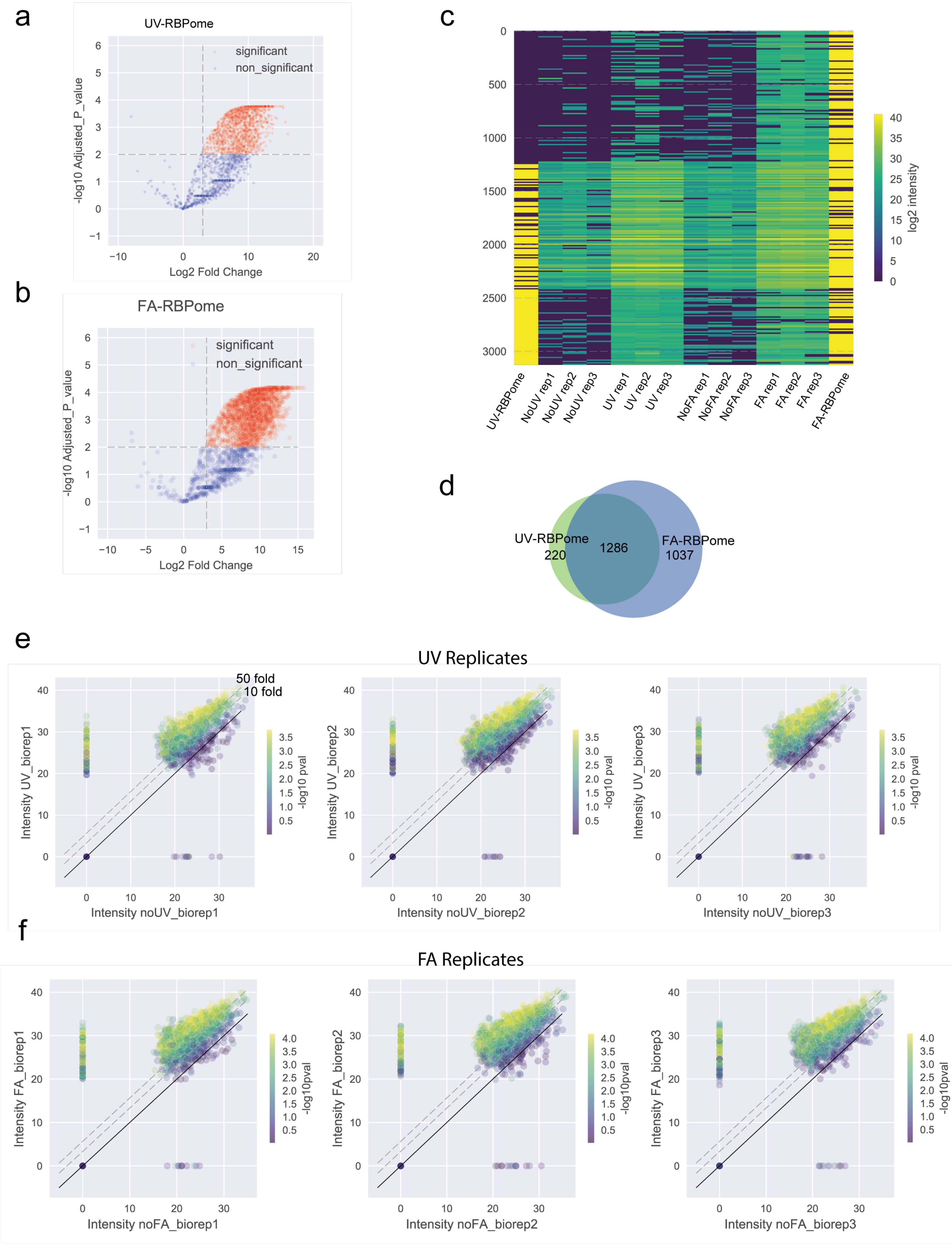

Supplementary Figure 2: Analysis of Interactome capture for UV and FA-RBPome.

a. Volcano plot displaying log2 fold change of UV crosslinked over non-crosslinked protein intensities versus -log10 adjusted p-values calculated using moderated t-test and applying Benjamini Hochberg correction for multiple hypothesis testing. Proteins showing a log2 fold change > 3 (right side of the vertical dotted line) and FDR of 1% (above horizontal dotted line) were selected as the UV-RBPome (red dots).

b. Volcano plot for *Drosophila* FA-RBPome as in (a) above.

c. K-means clustered Heatmap (k=3) representing the -log2 transformed iBAQ intensity (intensity based absolute quantification) values for each of protein groups detected in the interactome capture protocols. Each column represents a biological replicate with conditions described at the bottom. Proteins selected in the UV-RBPome are depicted with maximum intensity on the leftmost column of the heatmap. The proteins in FA-RBPome are shown with maximum intensity on the opposite side of the heatmap.

d. Overlap between the UV-RBPome and FA-RBPome.

e. Scatter plots of UV crosslinked protein intensities (UV-Biorep 1,2,3) vs the respective non-crosslinked protein intensities (no UV-Biorep 1,2,3). Each protein is represented by a dot, which is coloured according to the protein's corresponding -log10 adjusted p-values calculated as in (a). The majority of the selected interactome proteins (-log10 p-value > 2) lie above the tenfold change dashed grey line and fifty fold dashed line.

f. As in (e) for formaldehyde crosslinked samples.

Supplementary Figure 3

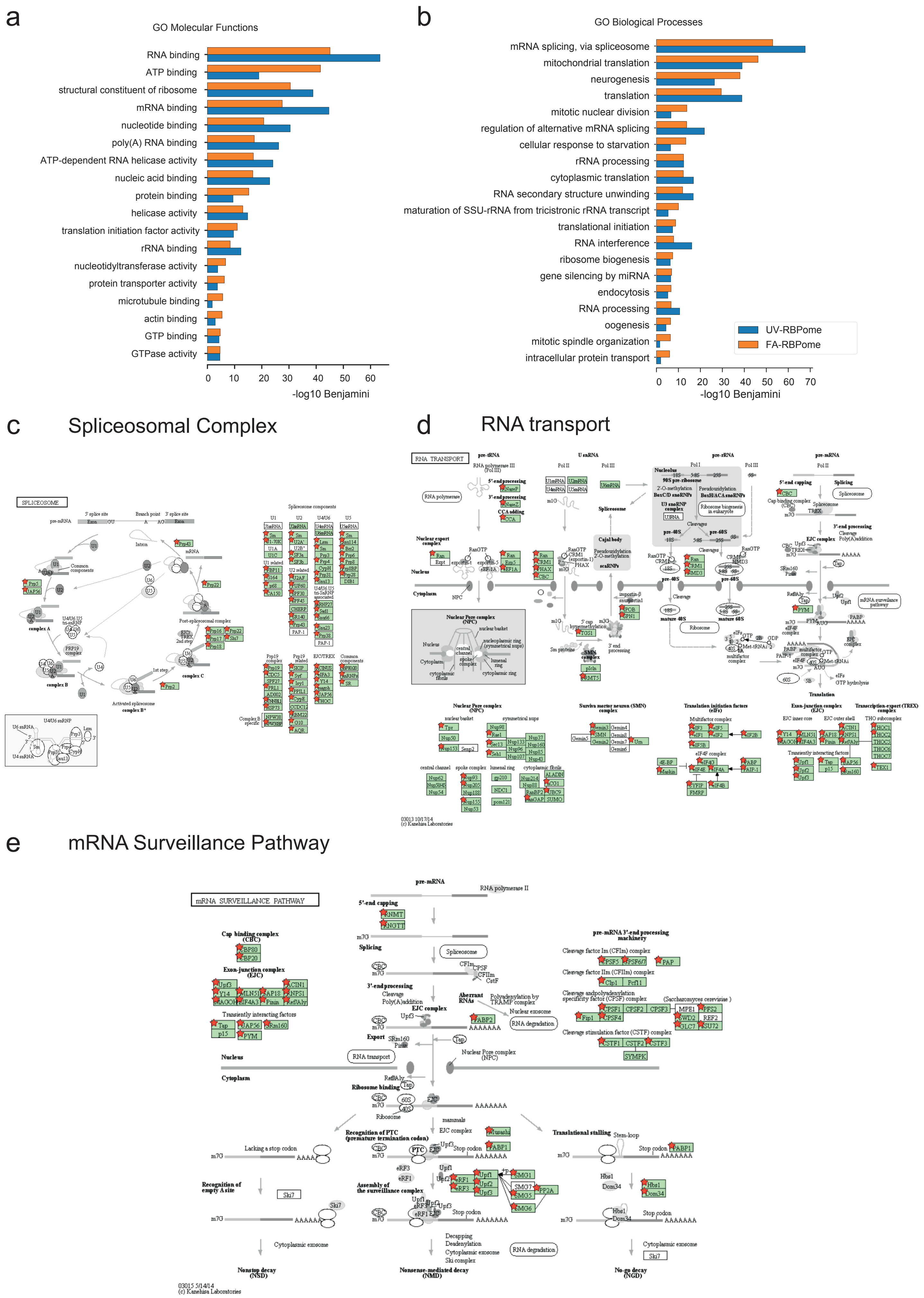

Supplementary Figure 3: Enriched Gene Ontology terms and enriched RNA related KEGG pathways

a. Bar chart of -log10 Benjamini adjusted p-values of enriched Gene Ontology terms for Molecular Functions (GO MF) using DAVID tool[34] in both UV- and FA-RBPomes.

b. As in (a) for Biological Processes (GO BP).

c. Generic Spliceosome KEGG pathway representing all *Drosophila* components in green boxes. The proteins belonging to each of the subcomplexes (like Prp19 or U2 complexes) are listed below with their respective names (Left). The proteins members of the FA-RBPome are denoted by red stars. Proteins in white boxes do not have any known *Drosophila* ortholog.

d. Generic RNA transport KEGG pathway annotated as in (c).

e. Generic mRNA surveillance KEGG pathway annotated as in (c).

Supplementary Figure: 4

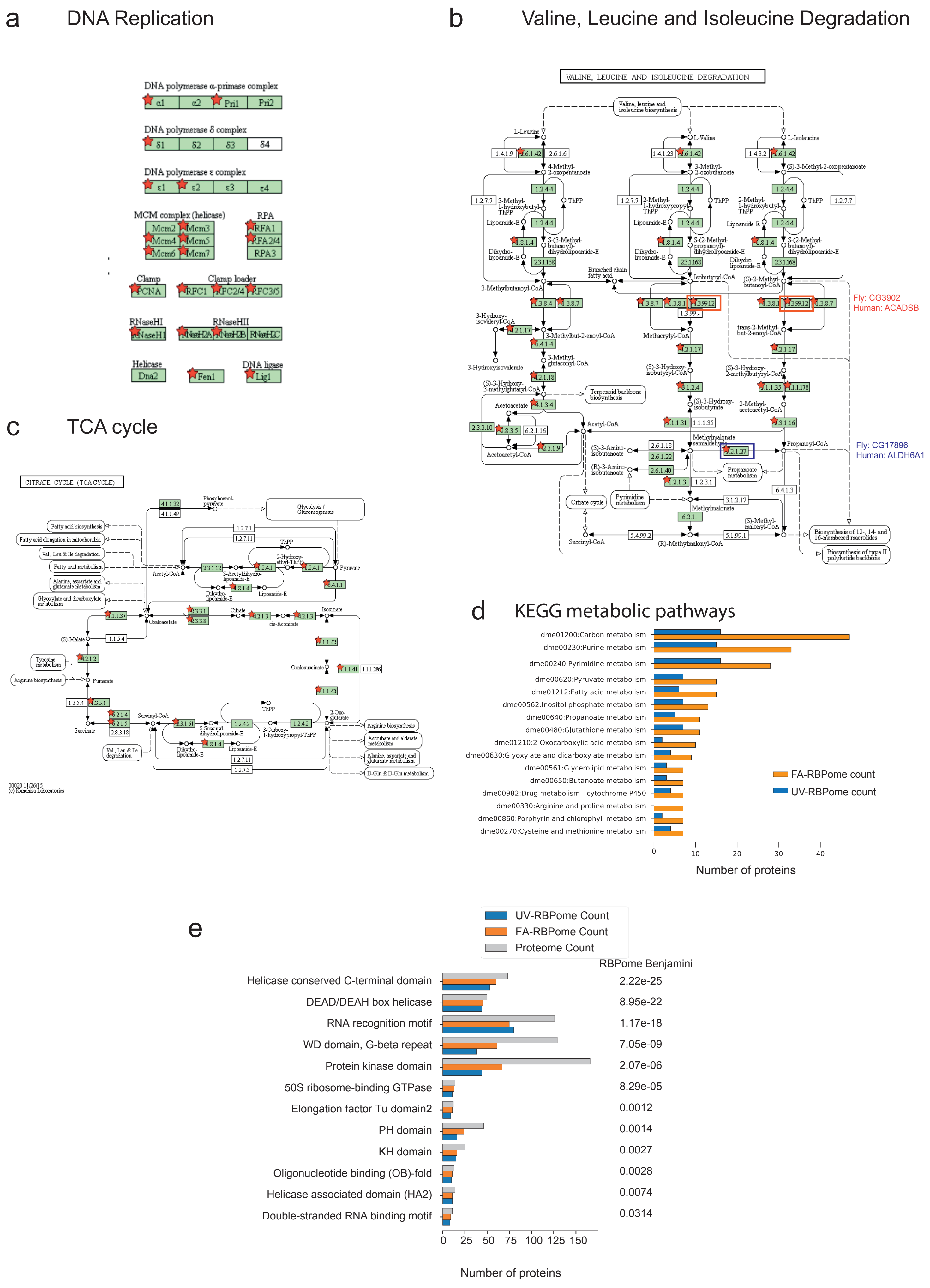

Supplementary Figure 4: KEGG pathways representing proteins from FA-RBPome and enriched Pfam domains in UV and FA RBPomes.

a. Members of the generic eukaryotic DNA replication machinery (from KEGG) representing all *Drosophila* components (green boxes) annotated for members from the FA-RBPome (red star).

b. Generic valine, leucine and isoleucine degradation KEGG pathway. Each of the enzymes is denoted by its KEGG enzyme number. Enzymes present in *Drosophila* are represented green boxes with a member of the FA-RBPome annotated with a red star. Proteins CG3902 (*Drosophila* ortholog of human ACADSB - orange box) and CG17896 (*Drosophila* ortholog of human ALDH5A1 - blue box) are highlighted with coloured boxes as they are validated to bind RNA in Fig. 6d.

c. Tricarboxylic acid (TCA) KEGG cycle as in (b).

d. Bar chart representing number UV- and FA-RBPomes protein members in some of the metabolic pathways (KEGG).

e. Bar chart representing the number of proteins with enriched Pfam domains. Protein counts from the entire proteome, UV-RBPome and FA-RBPome are represented for each domain. The Benjamini corrected p-values using DAVID tool[34] of only FA-RBPome are shown.

Supplementary Figure 5

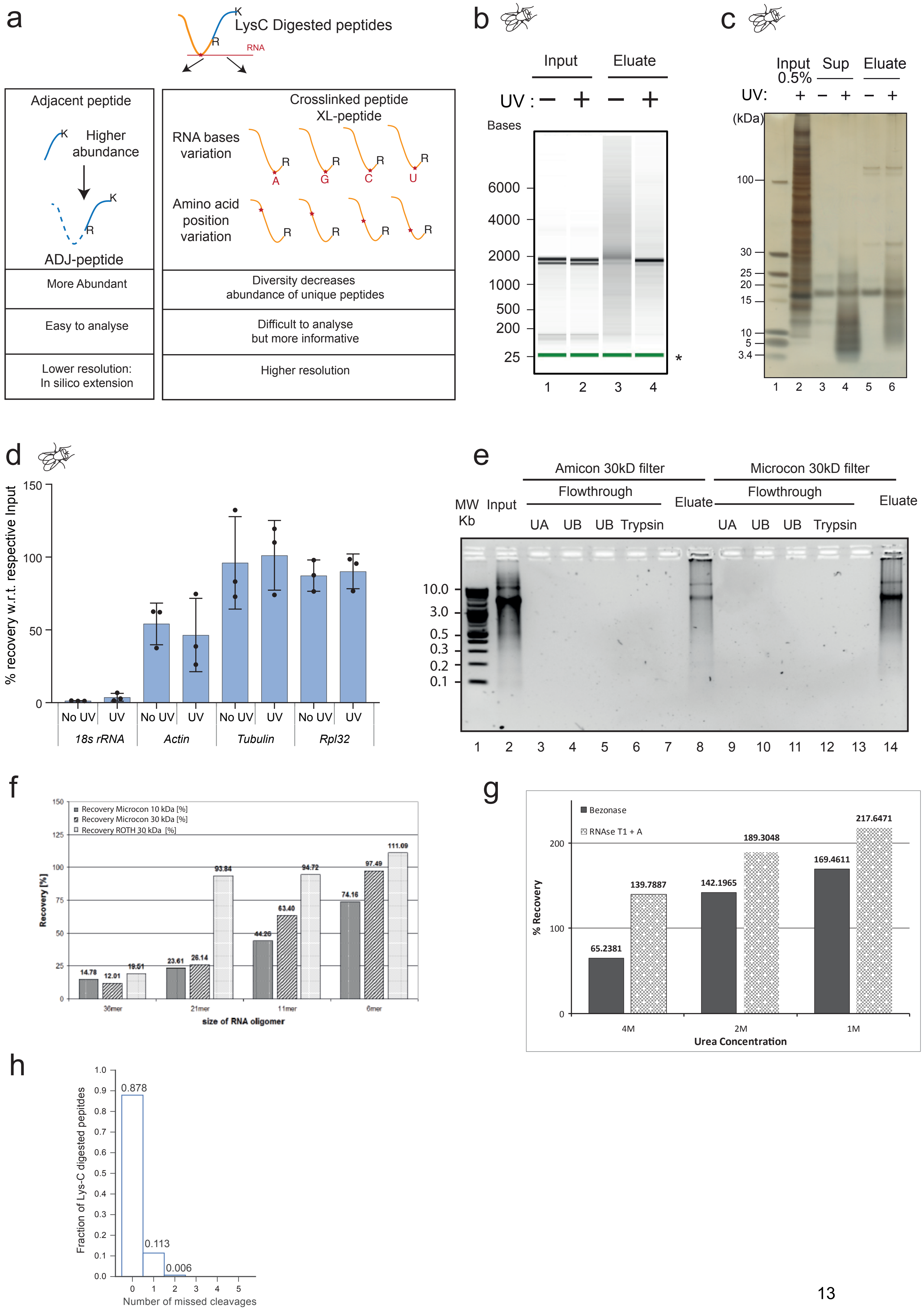

Supplementary Figure 5: RNA and protein controls for ProDIRP protocol.

a. Schematic representation of comparative characteristics of adjacent and XL-peptides. The right panel shows the origin of heterogeneity in crosslinked peptides. The adjacent peptide is homogenous and can be detected at higher abundance, however, it is extended in silico to the nearest Lys-C site.

b. RNA profiles of *Drosophila* RNA after on-bead digestion were detected by Bioanalyzer for no UV (-) / UV (+) inputs and eluates.

c. On-bead digestion of captured *Drosophila* interactome proteins was evaluated by observing profiles of released peptides in digested supernatant solution (Sup 5%) and eluted peptides (Eluate) from beads followed by digestion with RNase A.

d. *Drosophila* RNA quantitative PCR analysis was performed to show recovery and enrichment of mRNA (*GAPDH, HPRT, POLG*) as compared to 18s rRNA after on-bead digestion and elution. The recoveries of RNA were measured with respect to (w.r.t.) the respective input samples (no UV and UV). Error bars represent s.d. of three biological replicates. Source data are provided as a Source Data file.

e. Retention of PolA+ RNA by Amicon 30 kD MWCO and Microcon 30 kD MWCO filters. The RNA profile was analysed by gel electrophoresis: input (lane 2), RNA isolated from flowthrough (30%) after each stringent wash of RNA-FASP (lanes 3-6 and 9-12) (see methods for details), RNA collected from each of the filters by inversion (lanes 14).

f. Screening of commercial filters (Microcon 10 kD MWCO, Microcon 30 kD MWCO, Roth 30 kDa MWCO) to check for recovery of nucleic acid oligos of varying sizes (36, 21, 11 and 6 mers) passing through the filter by measuring UV absorbance of the oligos.

g. Optimisation of urea concentration in buffer for activity of Benzonase and RNase T1+A on RNA. RNA recovery from the Microcon 30 kD MWCO filters was evaluated by examining the RNA concentrations in input and RNAse-digested flowthrough using UV absorbance.

h. Missed cleavage observed in Lys-C cleaved peptides released on FASP filters and collected as flowthrough.

Supplementary Figure 6

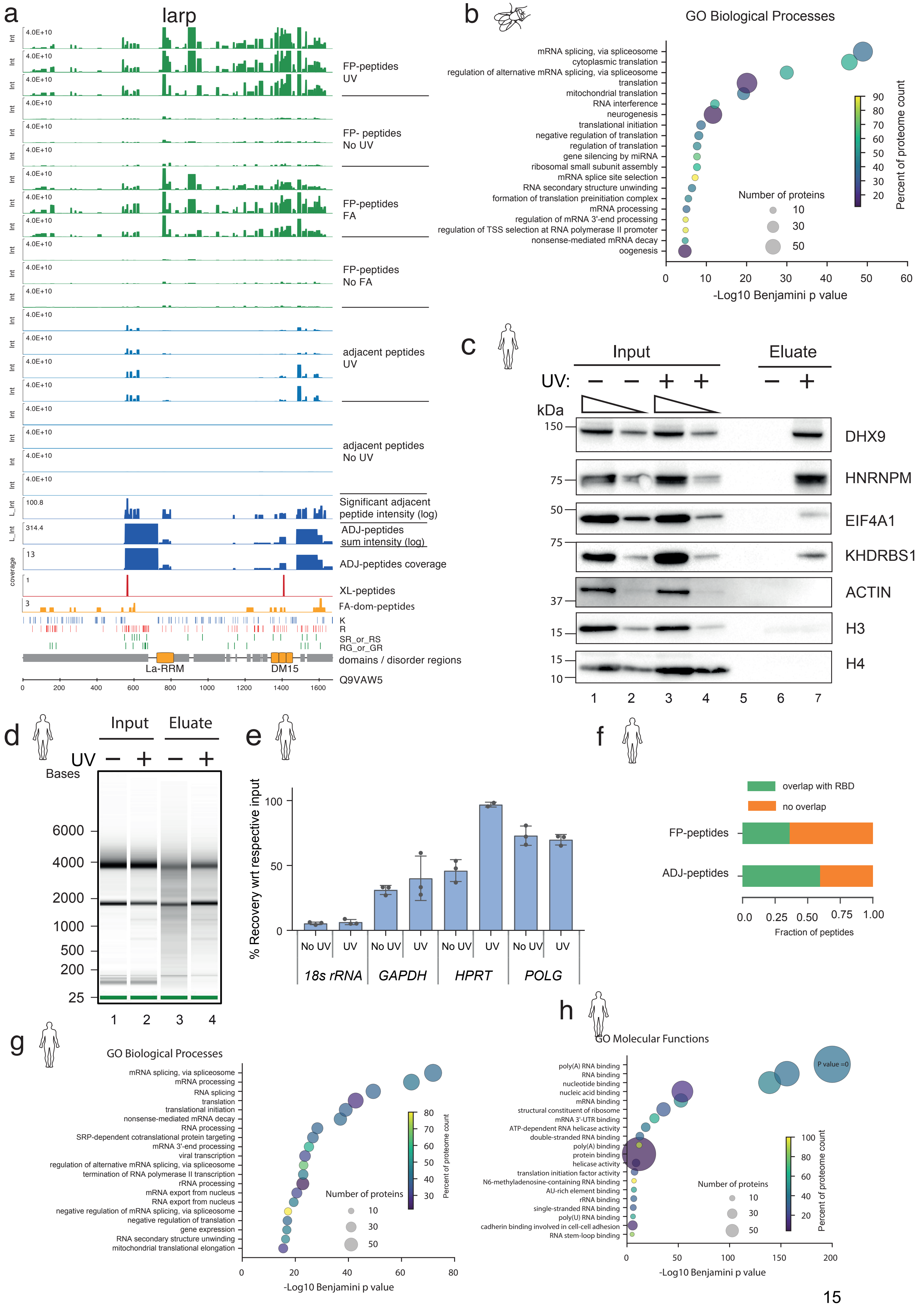

Supplementary Figure 6: Automated peptide mapping and Human CAPRI-RBPome.

a. An example of peptide intensity coverage plots for *Drosophila* larp protein. The tracks from top to bottom are as follows: full protein peptide intensities from the interactomes (UV, No UV, FA, No FA - green); intensities of adjacent peptides (UV, No UV - blue); Log average intensity of statistically significant adjacent peptides (blue), Log average intensity of extended adjacent peptides (ADJ-peptides, dark blue), crosslinked peptide coverage (XL-peptides), FA-dom-peptide coverage (orange), regular expression tracks showing tryptic cleavage sites (K and R), regular expression tracks showing RS and RG rich regions, protein profile annotated with InterPro domains (orange boxes) and disordered region (grey boxes). The peptide intensities for each of the biological replicates are scaled to the same maximum intensity.

b. Enriched Gene Ontology terms (Biological Processes) from Adjacent peptide protein interactome (ADJ-RBPome) in *Drosophila*.

c. Validation of the human interactome capture by Western blot of selected proteins. Antibodies against previously known *Drosophila* RNA binding proteins (DHX9, HNRNPM, EIF4A1, KHDRBS1) were used as positive controls, whereas antibodies against Actin, H3 and H4 were used as negative controls. Source data are provided in Supplementary Fig. 19.

d. On-bead Lys-C digested and eluted RNA profiles from human cells were analysed by Bioanalyzer and compared to input RNA (equal amount of RNA was loaded in all lanes).

e. Quantitative PCR analysis was performed in HEK293T cells to show recovery and enrichment of mRNA (*GAPDH, HPRT, POLG*) as compared to 18s rRNA after on-bead digestion and elution. The recoveries of RNA were measured with respect to the corresponding input (No UV / UV) samples. Error bars represent s.d. of three biological replicates, except the *HPRT* UV sample: s.d. of two biological replicates. Source data are provided as a Source Data file.

f. Fraction of peptides mapping to RNA binding domains in human FP-peptides and ADJ-peptides.

g. Enriched GO Biological Processes from ADJ-RBPome in humans.

h. As in (g) for GO Molecular Functions.

Supplementary Figure 7

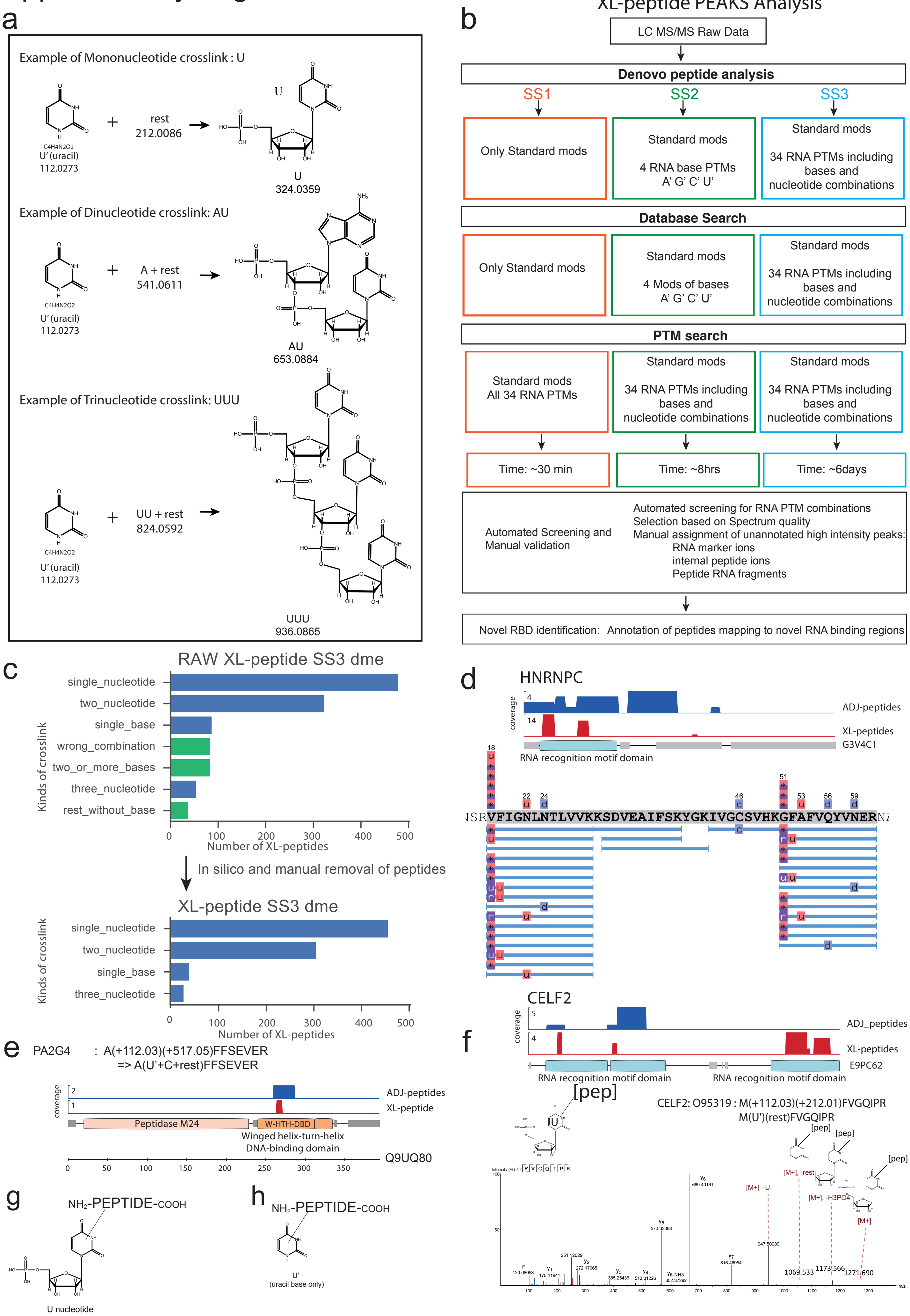

Supplementary Figure 7: Crosslinked peptide analysis by PEAKS.

a. Illustration of RNA PTM combinations exemplified by a single nucleotide (U), two nucleotides (AU) and three nucleotides (UUU). See Methods for details.

b. Workflow for analysis of crosslinked peptides by three search strategies in PEAKS. PEAKS consists of three steps of peptide identification: de novo peptide analysis, database search and PTM-search each with selected PTMs. Three search strategies (SS1,2,3) were used differing with respect to the search stage where RNA-PTMs are taken into consideration during the analysis. For search strategy 1 the RNA-PTMs are included only for the final PTM matching step and hence takes the least amount of time. In search strategy 2 the RNA base modifications (A',G',C',U') which remain covalently attached to the peptide fragments are used for de novo analysis. The rest of the RNA-PTMs are considered during the PTM search. In search strategy 3 all the RNA-PTMs are used in the de novo peptide analysis stage. The time required for each of the strategies is indicated as well. The crosslinked peptides were further pruned to remove wrong combinations of RNA-PTMs and remaining were manually curated. See Methods for details.

c. Bar charts showing classes of crosslinks observed in *Drosophila* XL-peptide raw data (Top) and *in silico* curated data (Below). See Methods for details.

d. (Top) Peptide coverage for human HNRNPC protein showing XL- and ADJ-peptides. (Below) Peptide coverage as seen in PEAKS software (amino acids 15-62) enabling visualisation of crosslinked peptide canonical peptide coverage. Each unique peptide is shown in blue mapped under the sequence. The nucleobase PTMs (A', G', C', U' modifications) are shown in red and "rest" containing PTMs of the nucleotides are shown in purple. Canonical PTMs are shown in blue. All the PTMs are stacked together on top of the protein sequence.

e. Sequence coverage for human protein PA2G4 / ebp1 depicting an XL-peptide and an ADJ-peptide in the DNA binding domain.

f. (Top) Peptide coverage for human protein CELF2. (Below) Annotated spectrum of a peptide with single nucleotide (U) crosslink.

g. Illustration of RNA-PTMs on a hypothetical peptide with the sequence PEPTIDE used to depict a canonical single nucleotide crosslink of uridine 5'-monophosphate (uridine nucleotide U = U'+rest).

h. As in (g) for a novel crosslink with uracil base only: U'

Supplementary Figure 8

a

	CAPRI Method	PolyA-RNA mediated crosslinked peptide<br>Kramer et al, 2014	RBD map<br>Castello et al, 2016<br>Liao et al, 2016	pCLAP<br>Mullari et al, 2017	RBR-ID<br>He et al, 2016
Isolation and detection RBDs	PolyA-RNA	PolyA-RNA	PolyA-RNA	PolyA-RNA	No isolation of RNA

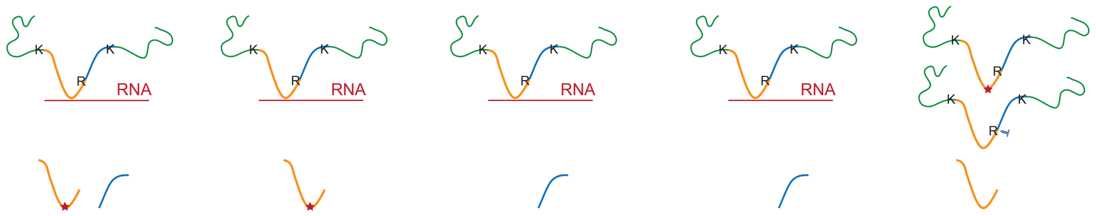

	CAPRI Method	PolyA-RNA mediated crosslinked peptide	RBD map	pCLAP	RBR-ID
	Crosslinked and adjacent peptide identification	Thio-U mediated Crosslinked peptide identification	Adjacent peptide and In silico Crosslinked site prediction	Adjacent peptide	Crosslinked peptide prediction by depletion
	Two step enrichement<br>1. PolyA enrichement<br>2. RNA FASP	Two step enrichement<br>1. PolyA enrichement<br>2. Size exclusion/TiO2	Two step enrichement<br>1. PolyA enrichement<br>2. PolyA enrichement	Single step enrichement<br>1. PolyA enrichement	No enrichment
Detection of crosslinked peptides	Software: PEAKS PTM<br>True detection<br>without prefilteration of spectra	Software: RNPxl<br>True detection<br>with prefilteration of spectra	Insilico site prediction<br>based on coverage of input,<br>released and bound peptides	Not detected	Crosslinked peptide prediction by depletion
Highest resolution possible	Single Amino acid<br>for XL-peptide.<br>LysC sites for ADJ-peptide	Single Amino acid	LysC peptide and<br>for XLink peptide: depends on<br>peptide coverage and sequence	LysC sites	Tryptic peptide.<br>No actual crosslink detected
Maximum stringency for disruption of non-specific RNA peptide interaction	1% LDS, 500mM LiCl<br>and<br>8M urea	0.5% LDS,<br>500mM LiCl	0.5% LDS,<br>500mM LiCl	2M Guanidium HCl<br>150mM NaCl	Not Applicable
Organisms	Drosophila,<br>Human	Yeast	Human and Mouse	Human	Mouse nuclei

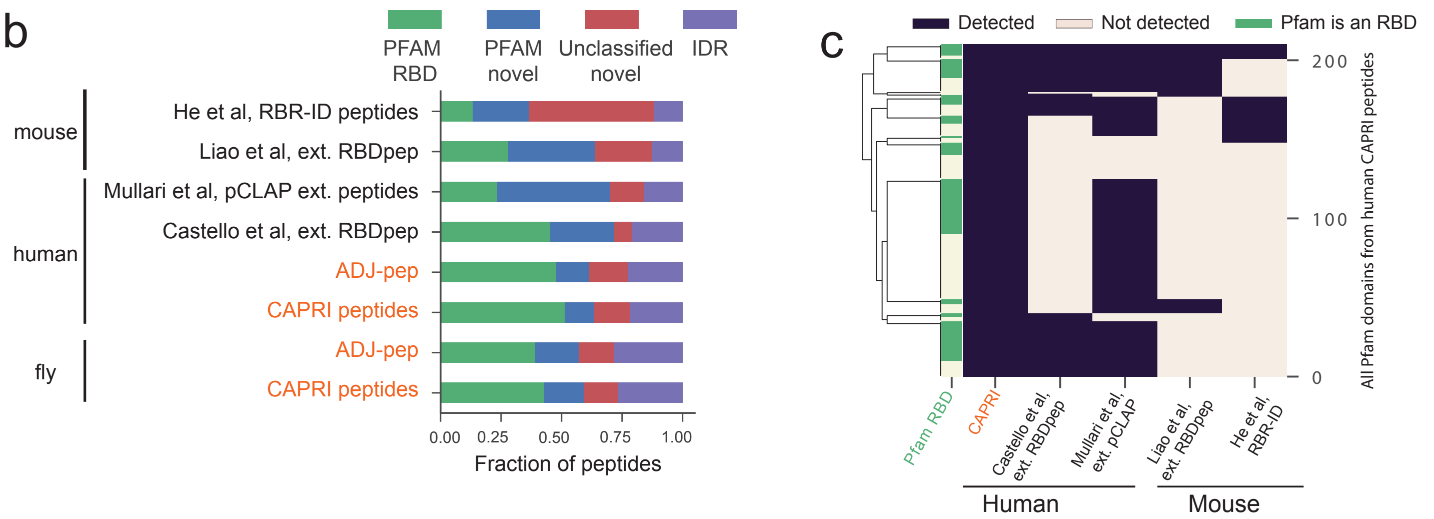

Supplementary Figure 8: Comparison of CAPRI with existing methods for identifying RNA binding regions.

a. Comparison of CAPRI technique with recently published RBD identification methods[8,35–38] highlighting the advantages of CAPRI.

b. Stacked bar chart showing the fraction of peptides identified in all the RBD capture experiments. The peptides are classified into four categories (PFAM RBD, new Pfam domain, IDR and the remaining unclassified novel regions.

c. Row-wise clustered heatmap representing the identification of all the human CAPRI PFAM domains along with their identification in other mammalian RBD capture methods[35–38]. The PFAM domain is marked in green if it is a known RBD.

Supplementary Figure 9

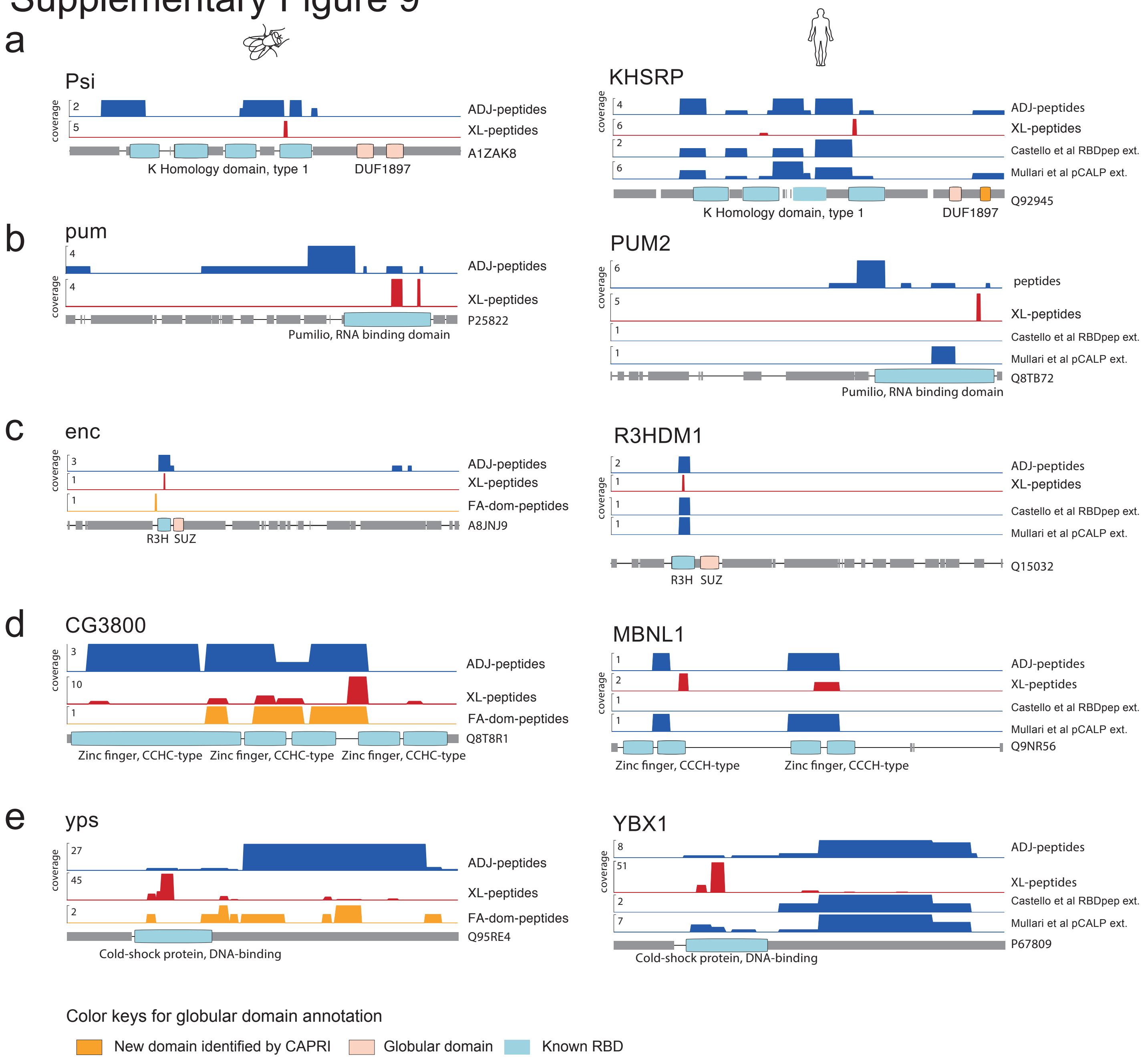

Supplementary Figure 9: Identification of RBDs by CAPRI peptides.

(a-e) Representation of peptide coverage for *Drosophila* (Left) and human orthologs (Right) with ADJ- (blue) and XL- peptides (red) identifying the canonical K-Homology domain and the non-canonical Pumilio, R3H, CCHC/CCCH zinc finger and Cold shock protein domains. Coverage profiles of FA-dom-peptides are depicted in *Drosophila* proteins when present. Coverage profiles of Castello extended RBDpeptides[36] and Mullari extended pCLAP peptides[38] are also depicted in the human proteins.

Supplementary Figure 10

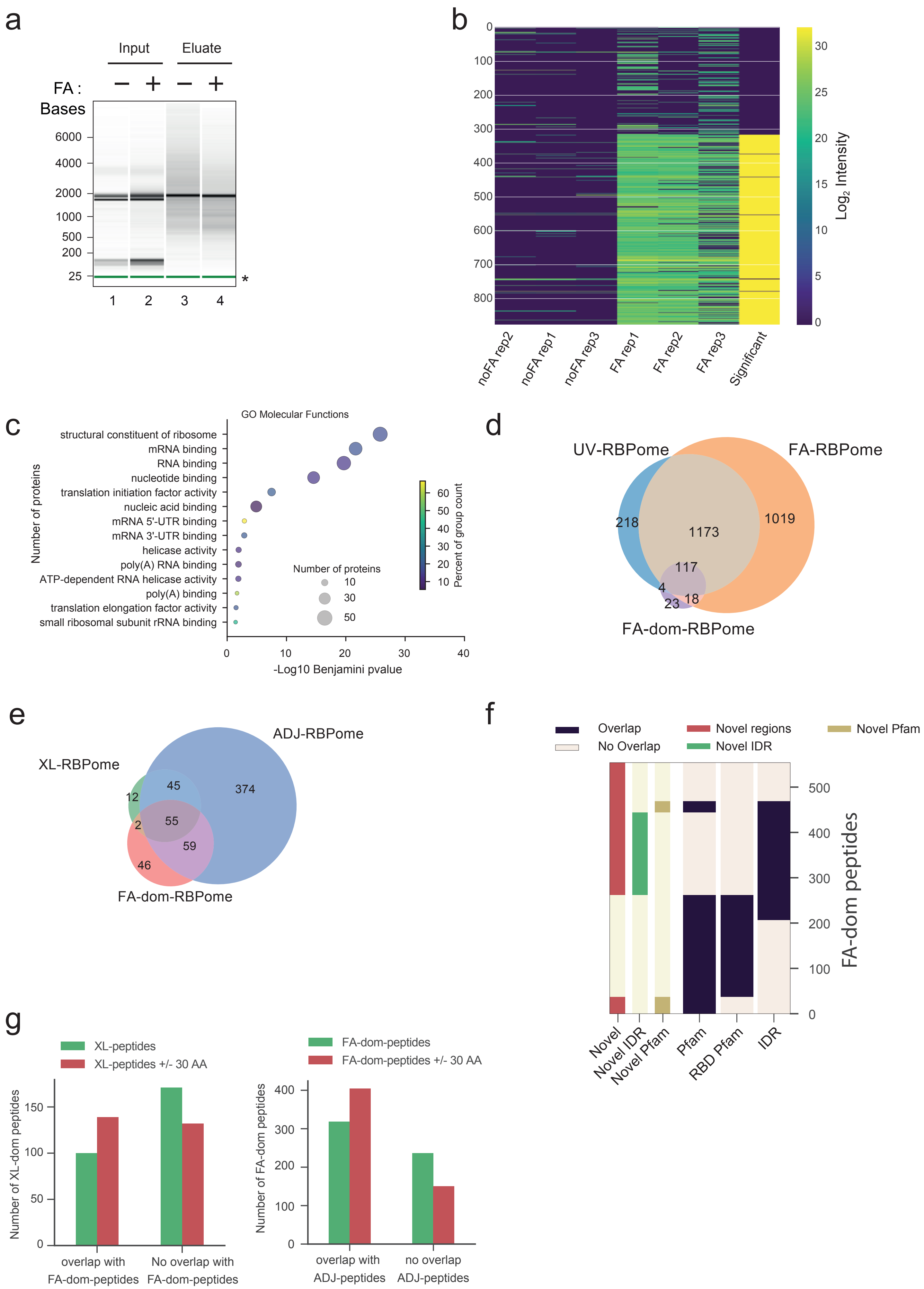

Supplementary Figure 10: FA domain peptides analysis.

a. RNA profiles for the FA based interactome capture in *Drosophila* cells analysed by Bioanalyzer and compared to input RNA (equal amount of RNA was loaded in all lanes). Asterisk marks 25 bases marker.

b. Clustered heatmap of peptide raw intensities (log_2) measured in three biological replicates. The FA-dom-peptides were selected by using a moderated t-test and a Benjamini Hochberg correction for multiple testing with an FDR of 0.15 followed by a heuristic cutoff of eight-fold intensity increase in the crosslinked relative to the non-crosslinked samples. They are depicted in the last lane with maximum intensity.

c. Enriched Gene Ontology terms (Molecular Functions) from FA-dom-peptide protein interactome (FA-dom-RBPome) in *Drosophila*.

d. Venn diagram representing the overlap of FA-dom-RBPome with the full protein interactomes UV- and FA-RBPomes.

e. Venn diagram showing the overlap of FA-dom-RBPome with the CAPRI interactomes ADJ- and XL-RBPomes.

f. Clustered heatmap representing the distribution of FA-dom-peptides overlapping with Pfam, RBD-Pfam and IDR. Different categories of peptides are annotated on the left of each panel.

g. Overlap between XL-peptides with or without extension and FA-dom-peptides (left). Overlap between FA-dom-peptides with or without extension and ADJ-peptides (right).

Supplementary Figure 11

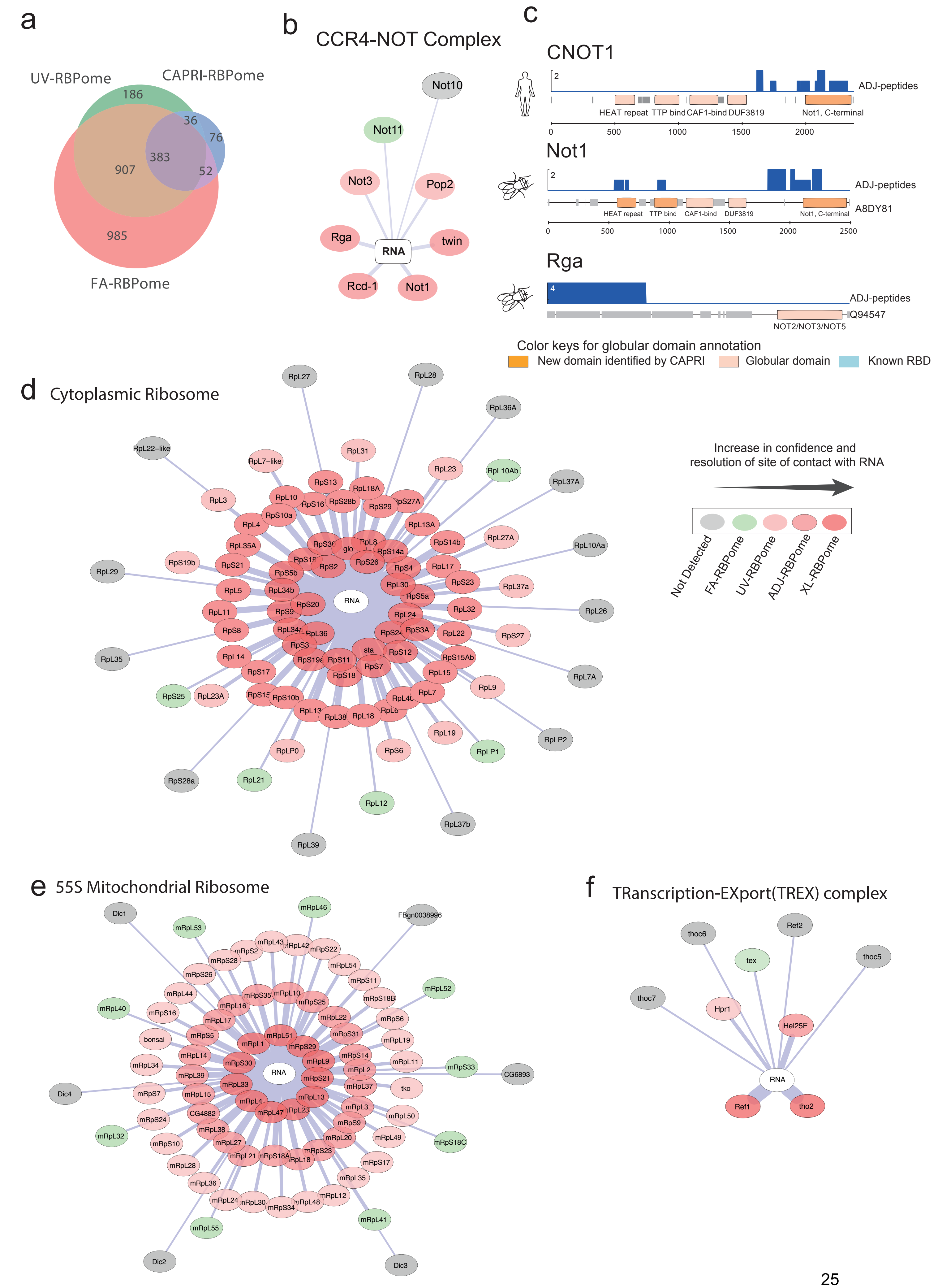

Supplementary Figure 11: Hierarchy of RNA protein complexes.

a. Overlap between the UV- and FA- and CAPRI-RBPomes.

b. A graphical representation of layered interactome for Ccr4-Not complex after combining information from *Drosophila* FA-, UV-, Adj- and XL- RBPomes. The proteins are arranged in concentric circles where the confidence and resolution of the site of interaction increases with increasing proximity to the RNA node (blue) shown in the centre. The layers going from outside to inside: not-detected (grey), FA-RBPome (green), UV-RBPome (light red), Adj-RBPome (red) and XL-RBPome (dark red).

c. The RNA interacting regions for CNOT1 and its fly ortholog Not1 in the top two protein profiles. Rga (a fly ortholog of human CNOT2) protein domain peptide coverage at the bottom.

d. As in (b) for cytoplasmic ribosome.

e. As in (b) for 55S mitochondrial ribosome.

f. As in (b) for TREX complex.

Supplementary Figure 12

a

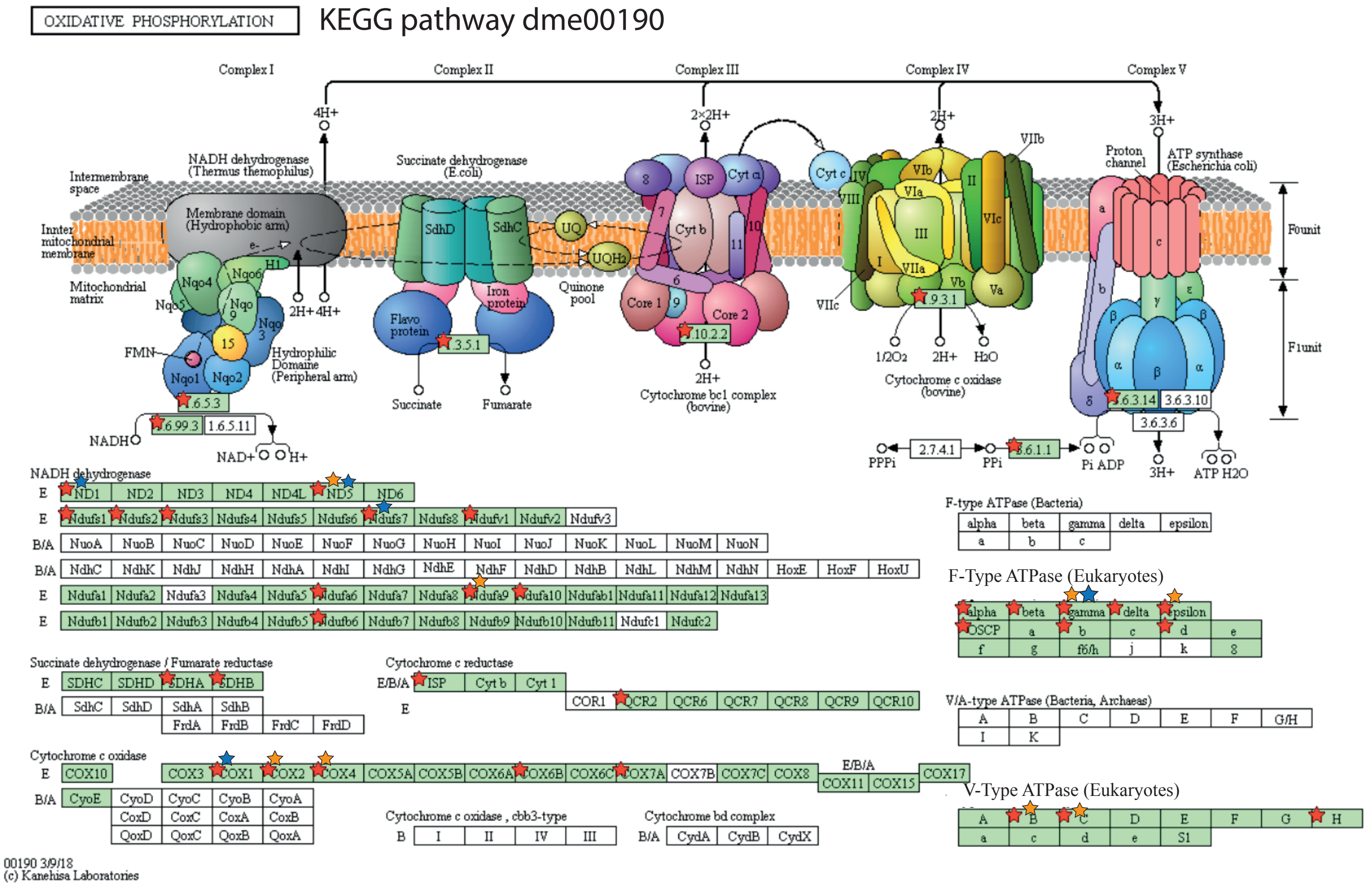

b

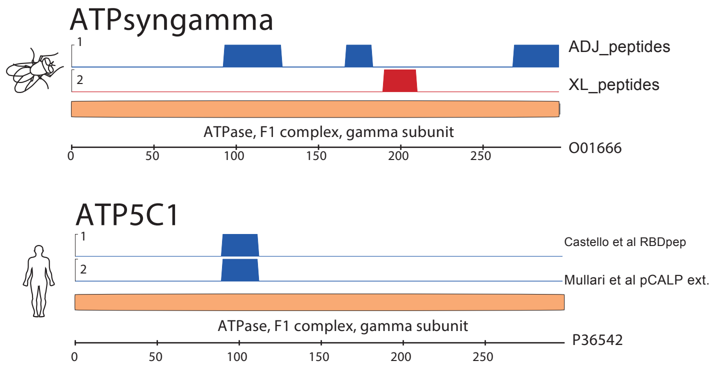

c

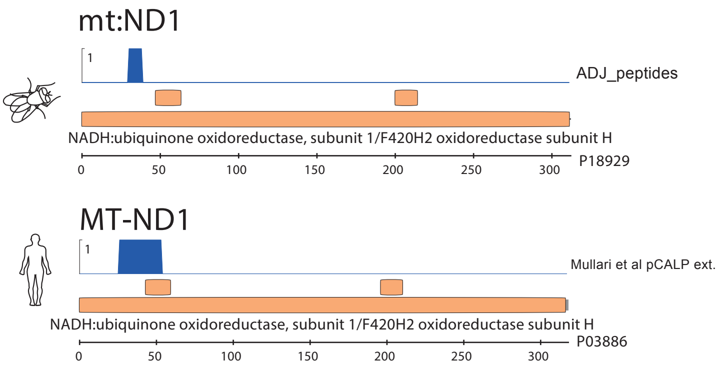

d

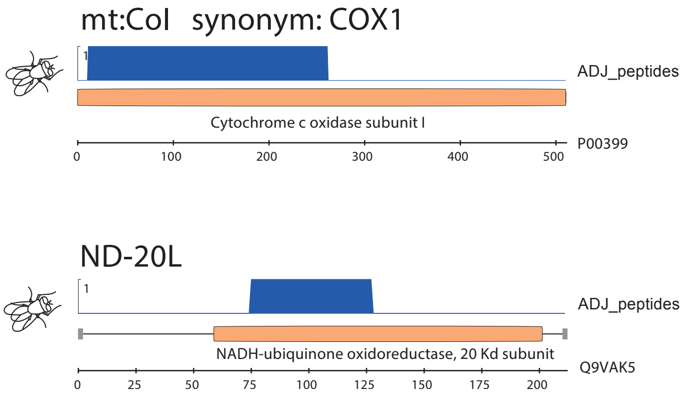

Supplementary Figure 12: RNA binding domains of proteins involved in oxidative phosphorylation.

a. Generic oxidative phosphorylation KEGG pathway representing all *Drosophila* components in green boxes. Proteins belonging to complexes I-V in the electron transport chain are listed below with their respective names (Below). Proteins which had been identified in the FA-, UV- and CAPRI-RBPomes are denoted by red,orange and blue stars, respectively. Proteins in white boxes do not have any known *Drosophila* ortholog.

b. Representation of peptide coverage for *Drosophila* and human orthologs of extramembranous subunit (F1) of mitochondrial membrane ATP synthase.

c. Peptide coverage for *Drosophila* mt:ND1 (mitochondrial NADH-ubiquinone oxidoreductase chain 1) and human MT-ND1 proteins.

d. Peptide coverage for *Drosophila* COX1 (Cytochrome c oxidase subunit 1) and ND-20L proteins (NADH dehydrogenase (ubiquinone) 20 kDa subunit-like).

Supplementary Figure 13

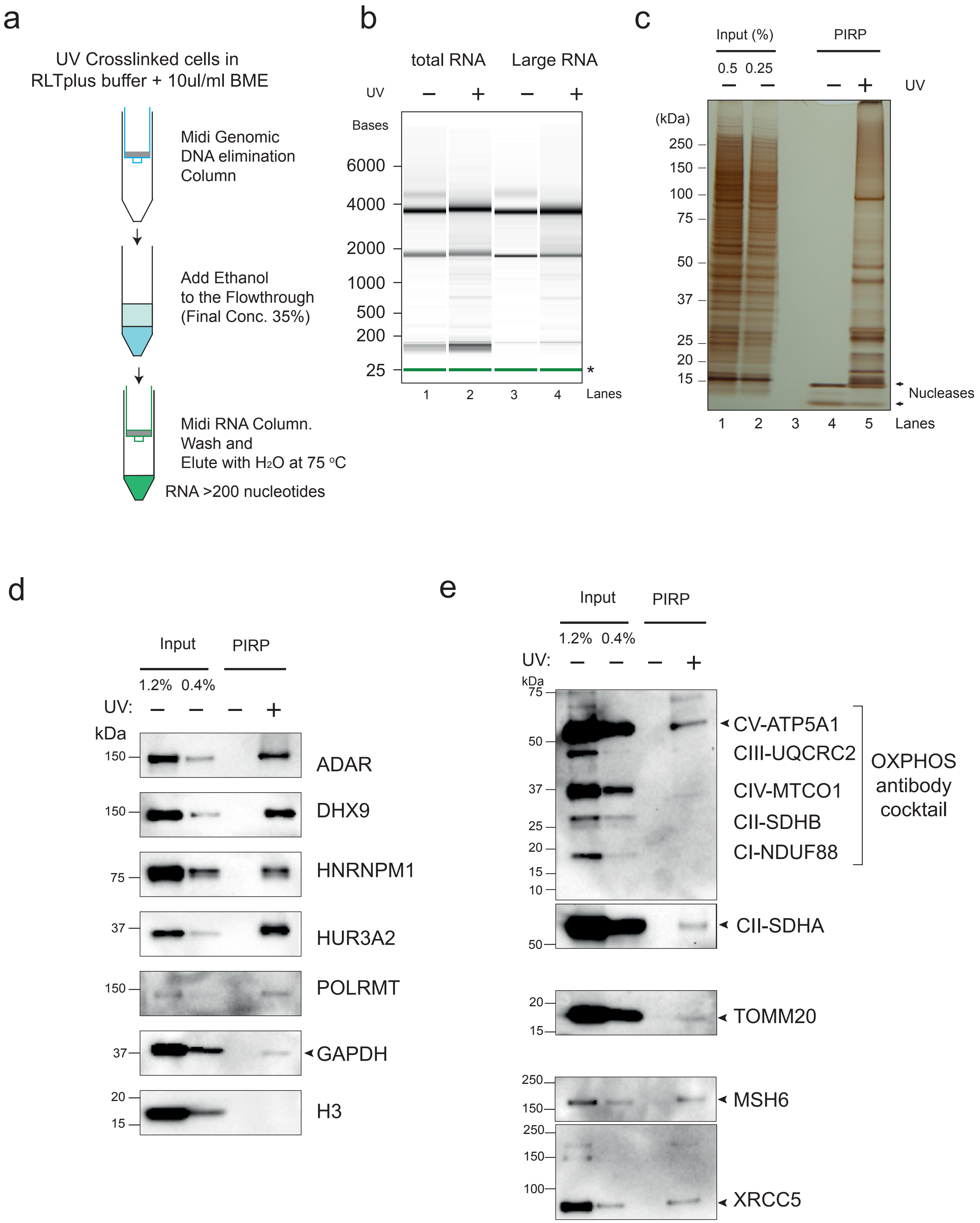

Supplementary Figure 13: Verification of new RNA binding proteins by large RNA interactome capture.

a. Workflow for isolating large RNA binding proteins. Briefly, UV irradiated and non-irradiated cells were resuspended in RLTplus buffer and passed through genomic DNA binding columns. Ethanol was added to the flowthrough to make up 35% of the final volume. The mixture was loaded on an RNA binding Midi column. The column was washed with RW1, RPE buffers and then dried by centrifugation. The RNA along with bound proteins was eluted from the column using hot nuclease-free water.

b. Bioanalyzer profiles of the eluted large RNA from crosslinked and non-crosslinked cells was analysed along with total RNA from the same cells. Asterisk denotes the 25 bases marker.

c. Silver stained protein profiles representing all large RNA bound proteins.

d. Western blots used to validate the PIRP protocol with classical RBPs (ADAR, DHX9, HNRNPM1, HUR3A2, POLRMT and non-classical RBPs like GAPDH. Source data are provided in Supplementary Fig. 20.

e. Validation of new RBPs involved in oxidative phosphorylation (ATP5A, SDHA), in tRNA import and protein import (TOMM20) and in DNA repair (MSH6 and XRCC5). Source data are provided in Supplementary Fig. 21.

Supplementary Figure 14

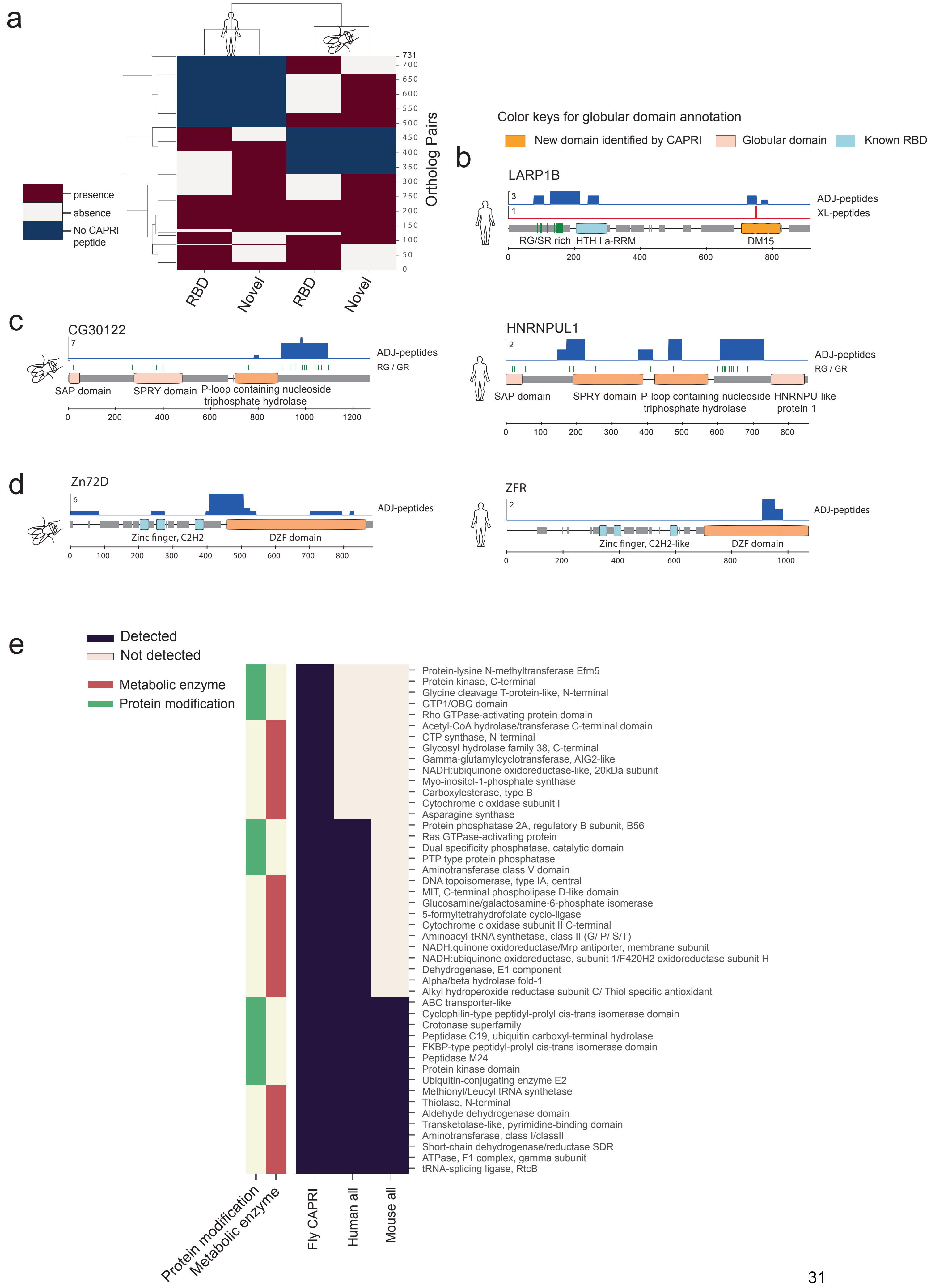

Supplementary Figure 14: Conserved novel globular domains.

a. Clustered heatmap showing the identification of Pfam-RBD or novel domains by peptides for each of the ortholog pairs.

b. Peptide coverage for human LARP1B.

c. Domain peptide coverage for *Drosophila* CG30122 and human HNRNPUL1 proteins.

d. Domain peptide coverage for *Drosophila* Zn72D and human ZFR proteins.

e. New metabolic and protein modification related Pfam domains identified in *Drosophila*.

Supplementary Figure 15

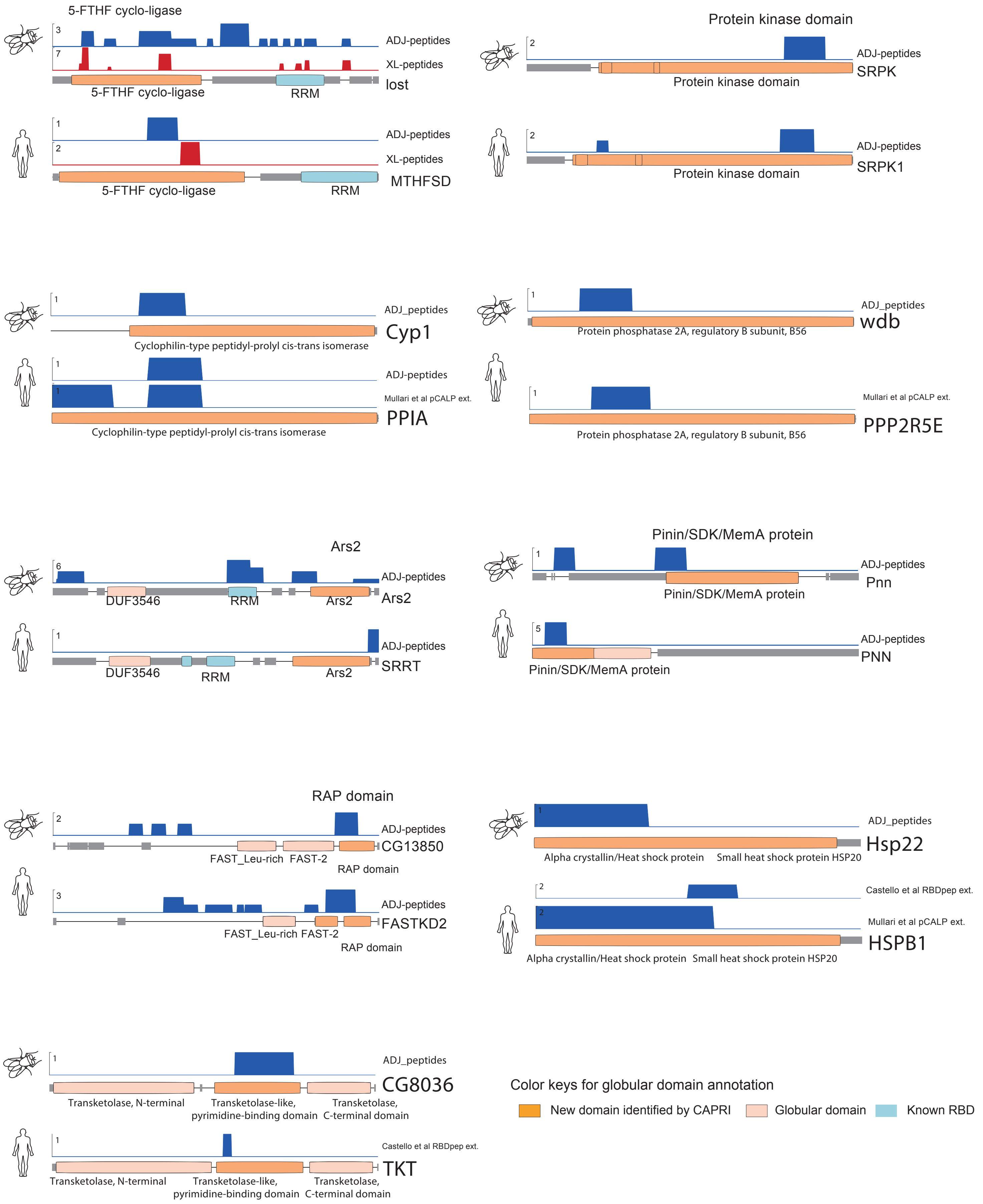

Supplementary Figure 15: New Pfam domains detected in *Drosophila* and humans.

Protein coverage profiles of all newly identified conserved RNA binding domains summarised in Fig. 8f.

Supplementary Figure 16

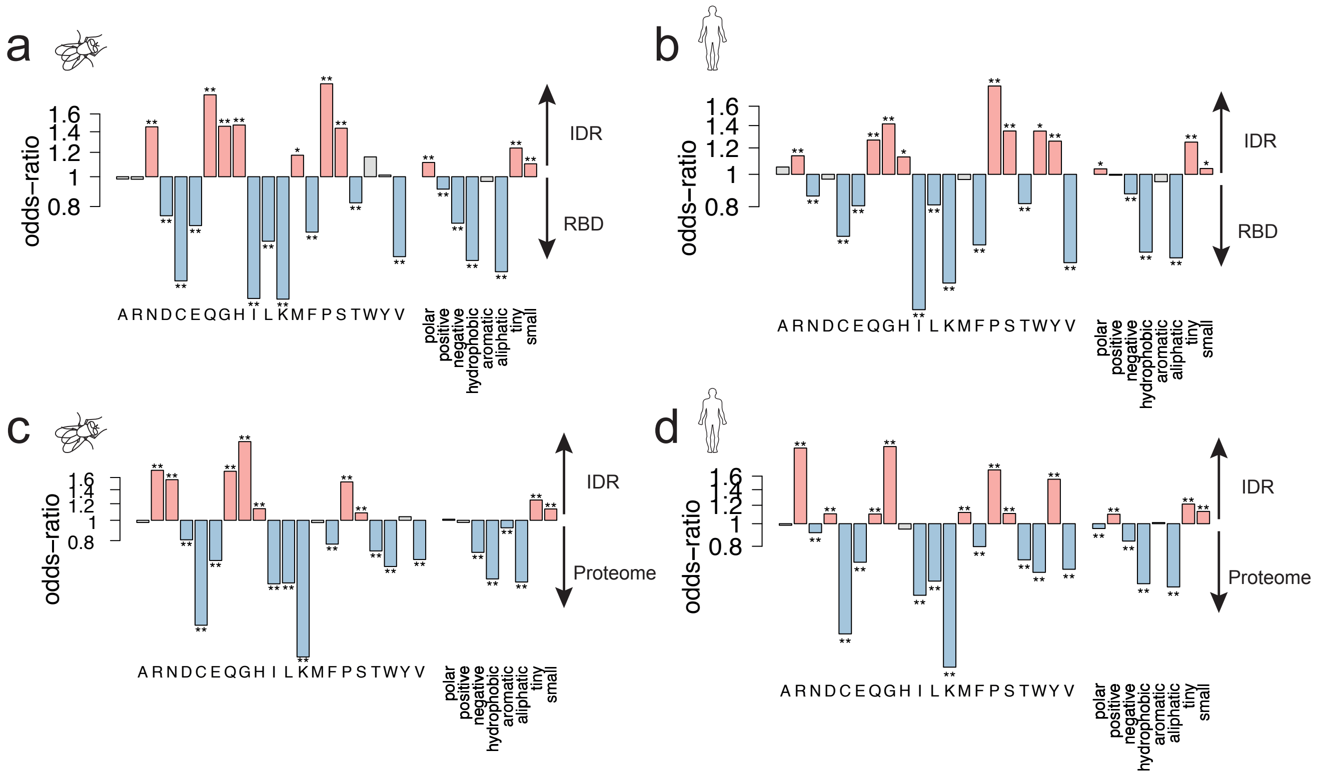

e ADJ-peptides Disordered vs Full protein RBPome

	Motif	Logo	E-value	Unerased E-value
1.	GGG		3.6e-072	3.6e-072
2.	RGG		5.5e-058	4.7e-065
3.	QZQ		6.6e-040	6.6e-040
4.	RBB		2.6e-028	2.6e-028
5.	GSS		1.3e-027	1.1e-029
6.	GGA		7.3e-027	2.2e-036
7.	GGB		1.5e-025	1.3e-045
8.	RBR		2.3e-024	2.7e-026
9.	GGS		1.5e-023	3.6e-041
10.	GPG		5.7e-022	1.1e-028
11.	BBN		3.0e-021	2.2e-025
12.	PPP		3.1e-018	3.1e-018
13.	SRS		3.8e-017	4.6e-017
14.	RGB		6.7e-016	6.7e-016
15.	GGP		2.4e-015	1.6e-033
16.	RRB		4.8e-015	2.7e-021
17.	RGS		3.5e-014	6.3e-017
18.	SBS		5.9e-014	3.5e-014
19.	PQQ		3.2e-013	1.8e-024
20.	PPR		5.9e-013	1.6e-018

f ADJ-peptides Disordered vs Full protein RBPome

	Motif	Logo	E-value	Unerased E-value
1.	RGG		1.7e-027	1.7e-027
2.	PPP		2.4e-021	2.4e-021
3.	GRG		1.6e-017	1.6e-027
4.	GGG		1.4e-014	1.1e-021
5.	BRG		2.2e-013	2.0e-020
6.	PPR		2.4e-010	3.4e-013
7.	SSS		1.5e-009	1.5e-009
8.	PGG		3.6e-009	1.6e-010
9.	RRG		5.5e-009	4.2e-012
10.	RRP		5.7e-008	1.3e-007
11.	RGR		1.9e-007	5.2e-025
12.	GSG		3.0e-007	2.7e-010
13.	GYG		2.5e-006	7.2e-010
14.	SYS		3.6e-006	9.9e-007
15.	QPP		2.0e-005	5.8e-007
16.	RSG		6.5e-005	2.4e-006
17.	PLP		2.5e-004	5.8e-008
18.	RGB		2.8e-004	1.0e-009
19.	SRP		2.9e-004	7.1e-006
20.	RRJ		8.5e-004	1.3e-003

Supplementary Figure 16: Amino acid composition comparison.

a, b. Amino Acid enrichment analysis of ADJ-peptides mapping to IDRs versus those mapping to globular RBDs of the respective organisms. Fisher's exact test was used and the p-values were corrected for multiple hypothesis testing by Benjamini-Hochberg correction (*, 10% FDR and **, 1% FDR) as in Castello et al[36].

c,d. Amino acid enrichment analysis of ADJ-peptides mapping to IDRs versus total proteome of the respective organisms. Statistical testing as in (a, b).

e,f. Discriminative Regular Expression Motif Elicitation (DREME) analysis[39]. Representation of enriched amino acid triplets in ADJ-peptides mapped to disordered regions versus sequences of all the proteins in the respective RBPomes of *Drosophila* (e) and humans (f). The E-value is enrichment p-value (Fisher's Exact test) times the number of candidate motif counts. The counts are calculated either after erasing (or without erasing) the sites that matched previously found motifs.

Supplementary Figure 17

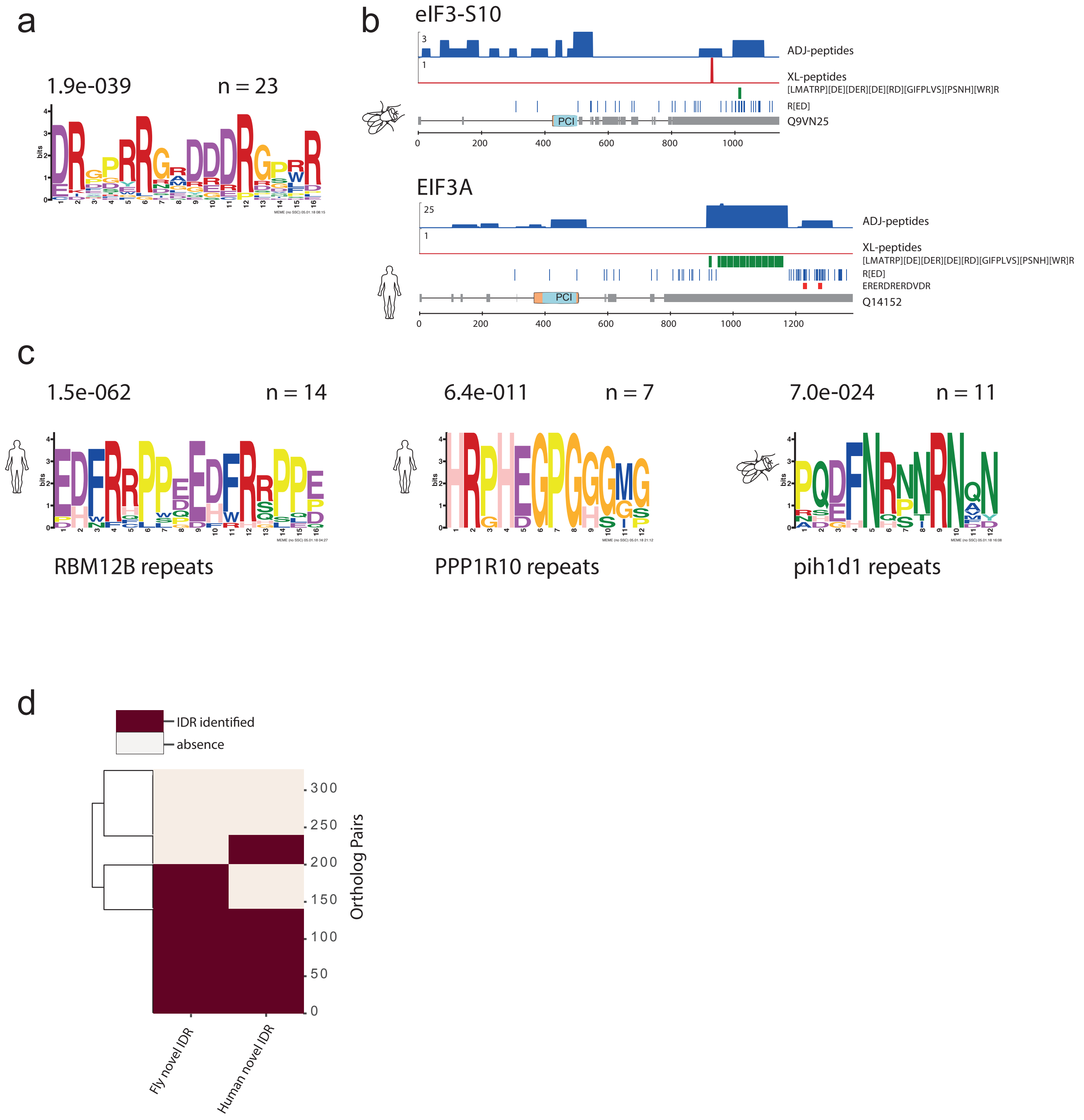

Supplementary Figure 17: Repeat motifs identified in *Drosophila* and humans.

a. Repeat motif identified in EIF3A (human) and eIF3-S10 (*Drosophila*) using MEME Motif tool. E-values as calculated by the MEME tool[30] and represent a modified p-value from a Fisher's t-test. The number of sites (n) are shown for each of the motifs.

b. Peptide coverage for human protein EIF3A and *Drosophila* ortholog eIF3-S10. Repeats matching the Regular Expression representing the repeat motif are shown in green. Other RD/E repeats are shown in blue.

c. Repeat motifs observed in RBM12B, PP1R10 and pih1d1 proteins annotated as in (a).

d. Clustered heatmap of ortholog pairs representing whether a novel IDR was recognised in the ortholog of the respective species.

Supplementary Figure 18

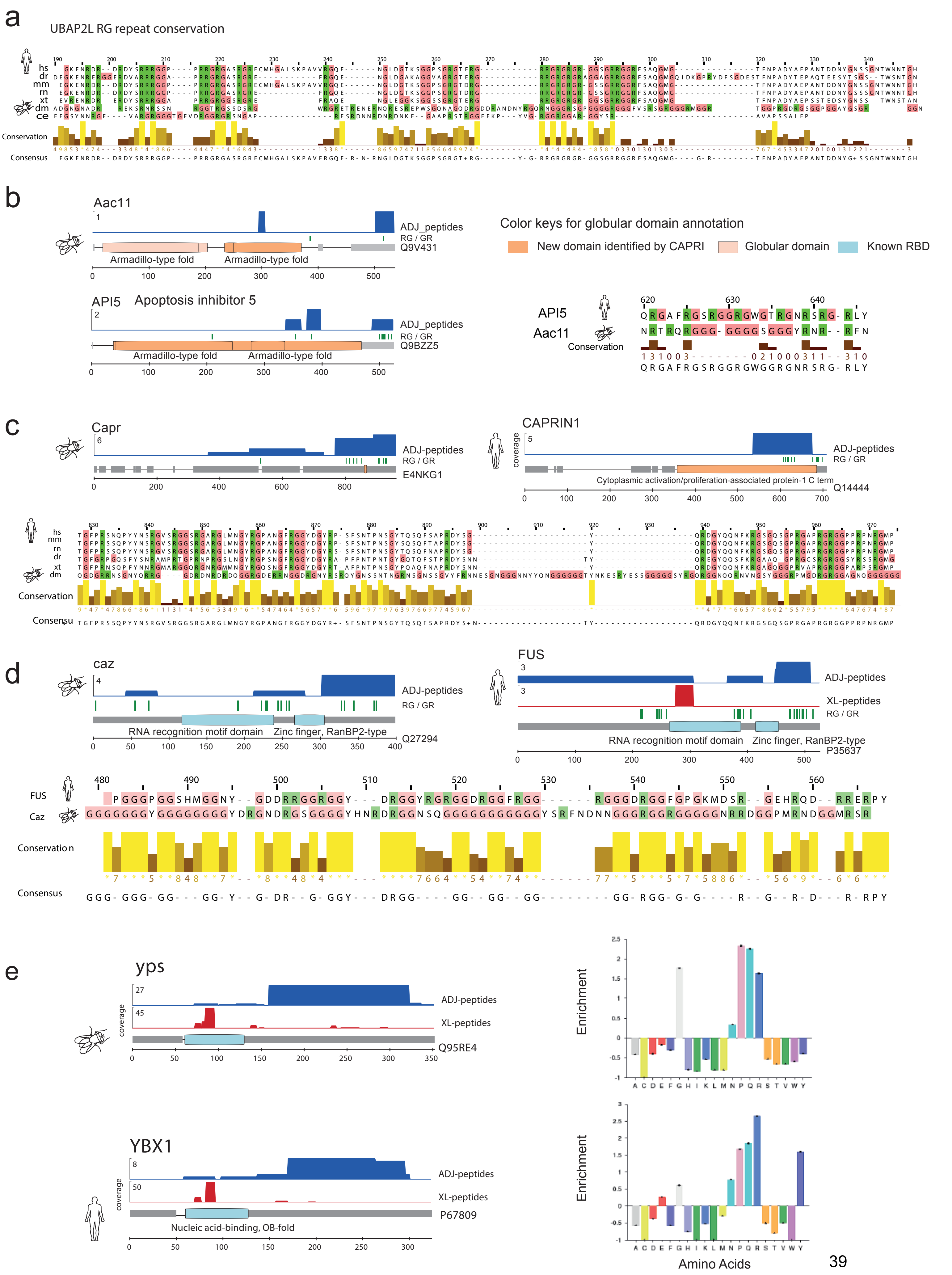

Supplementary Figure 18: Conservation of novel RNA binding IDRs.

a. Sequence conservation of RG repeats in human protein UBAP2L and *Drosophila* protein Lig including other model organisms. The conservation bar and consensus sequence are shown below the alignment. The R and G residues are coloured in green and pink respectively.

b. (Left) Peptide coverage representing the conservation of C-terminal peptide in API5 and Aac11. (Right) Sequence alignment of the same region annotated using the same colour scale as in (a).

c. (Top) Peptide coverage representing the conservation of C-terminal RNA binding region in Capr (Left) and CAPRIN1 (Right). (Below) Multiple sequence alignment of the C terminal region of CAPRIN1 with model organisms annotated using the same colour scheme as in (a).

d. (Top) Peptide coverage representing the conservation of C-terminal RNA binding region in caz (Left) and FUS (Right). (Below) Multiple sequence alignment of the C terminal region of CAPRIN1 with model organisms annotated using the same colour scheme as in (a). Only *Drosophila* and human sequences are shown.

e. Peptide coverage tracks (Left) of cold shock domain proteins yps (*Drosophila*) and YBX1 (human). Amino acids enriched (Right) in the disordered regions covered by CAPRI peptides in each of the proteins Yps and YBX1 proteins are evaluated by Composition Profiler tool[40].

Supplementary Figure 19

Uncropped blots for Fig. 2b

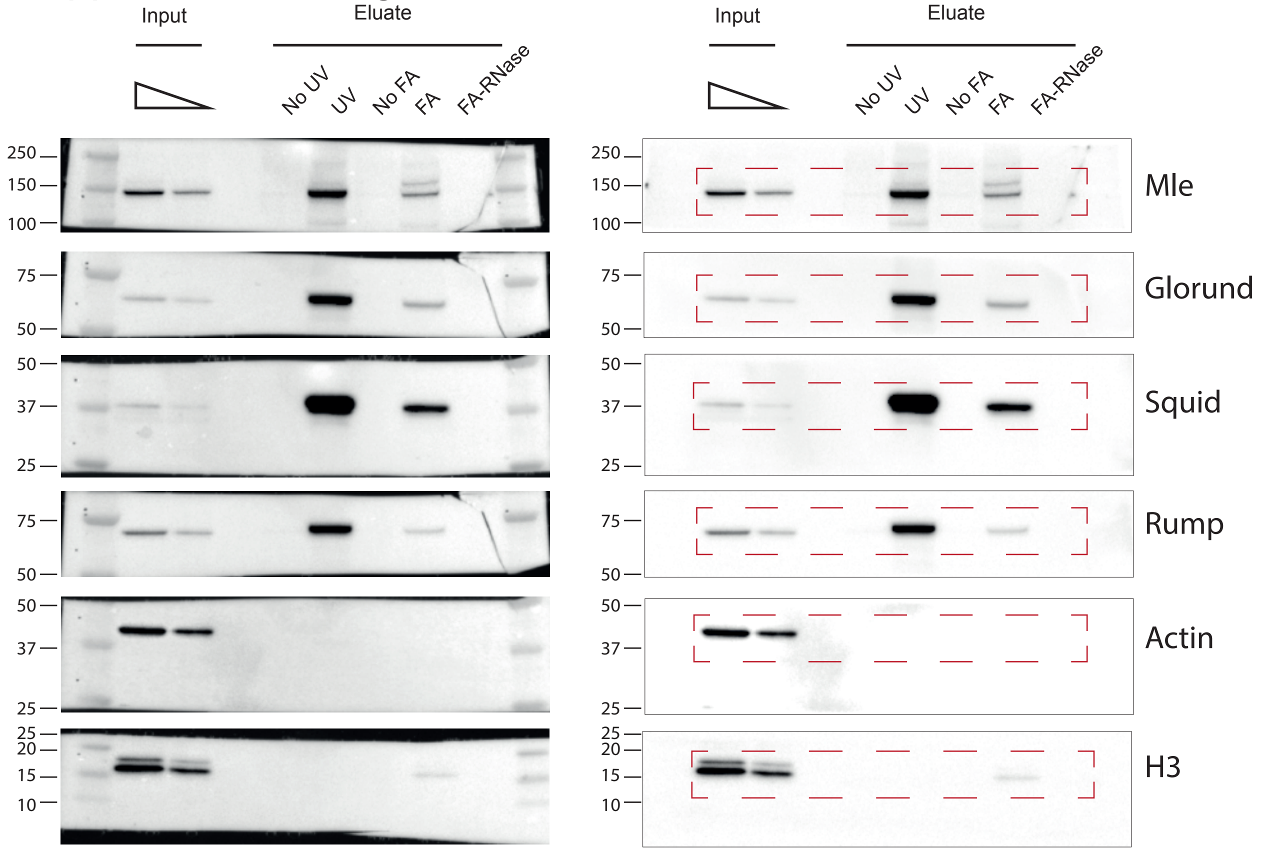

Uncropped blots for Supplementary Fig. 6c

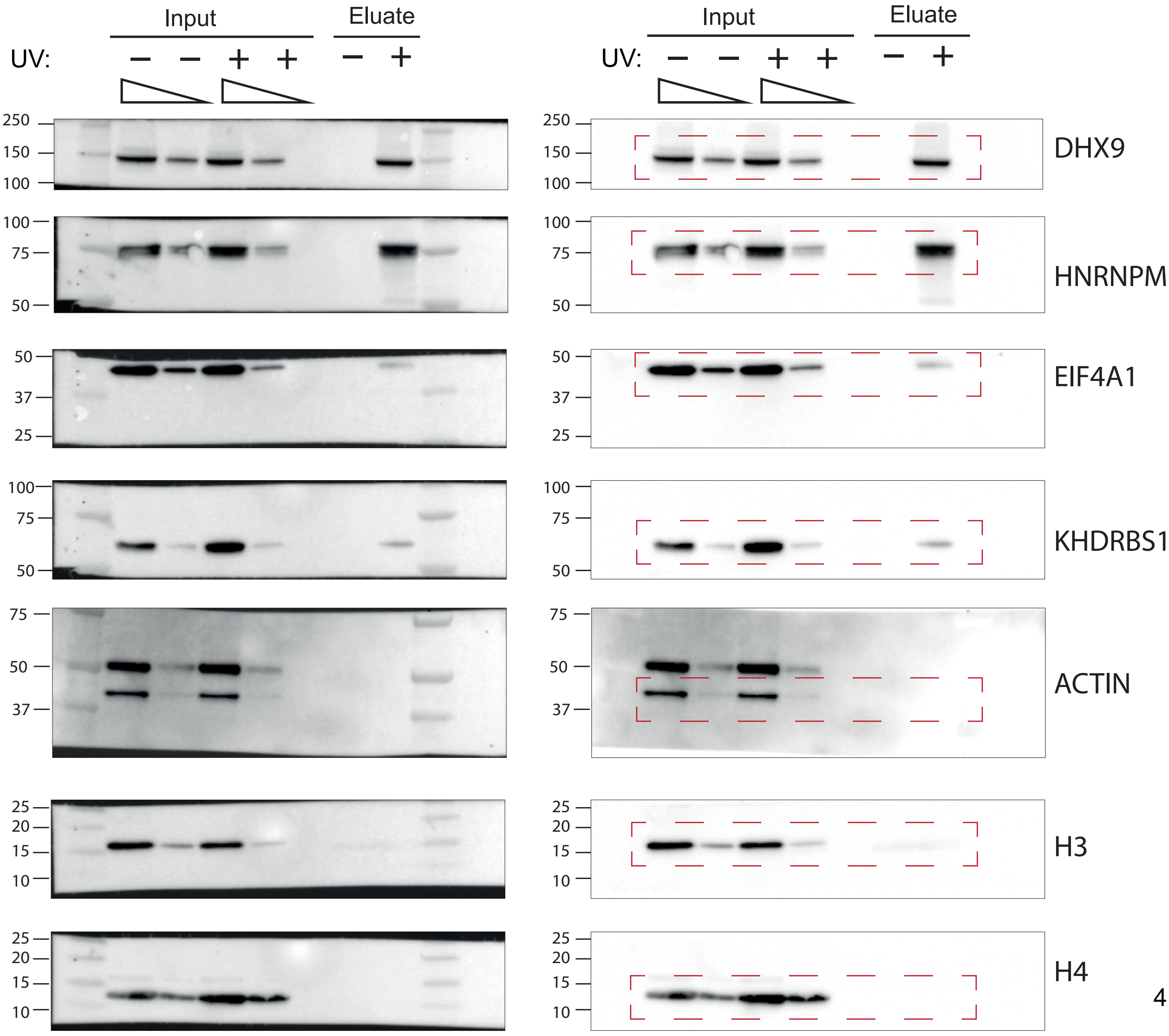

Supplementary Figure 20

Uncropped blots for Supplementary Fig. 13d

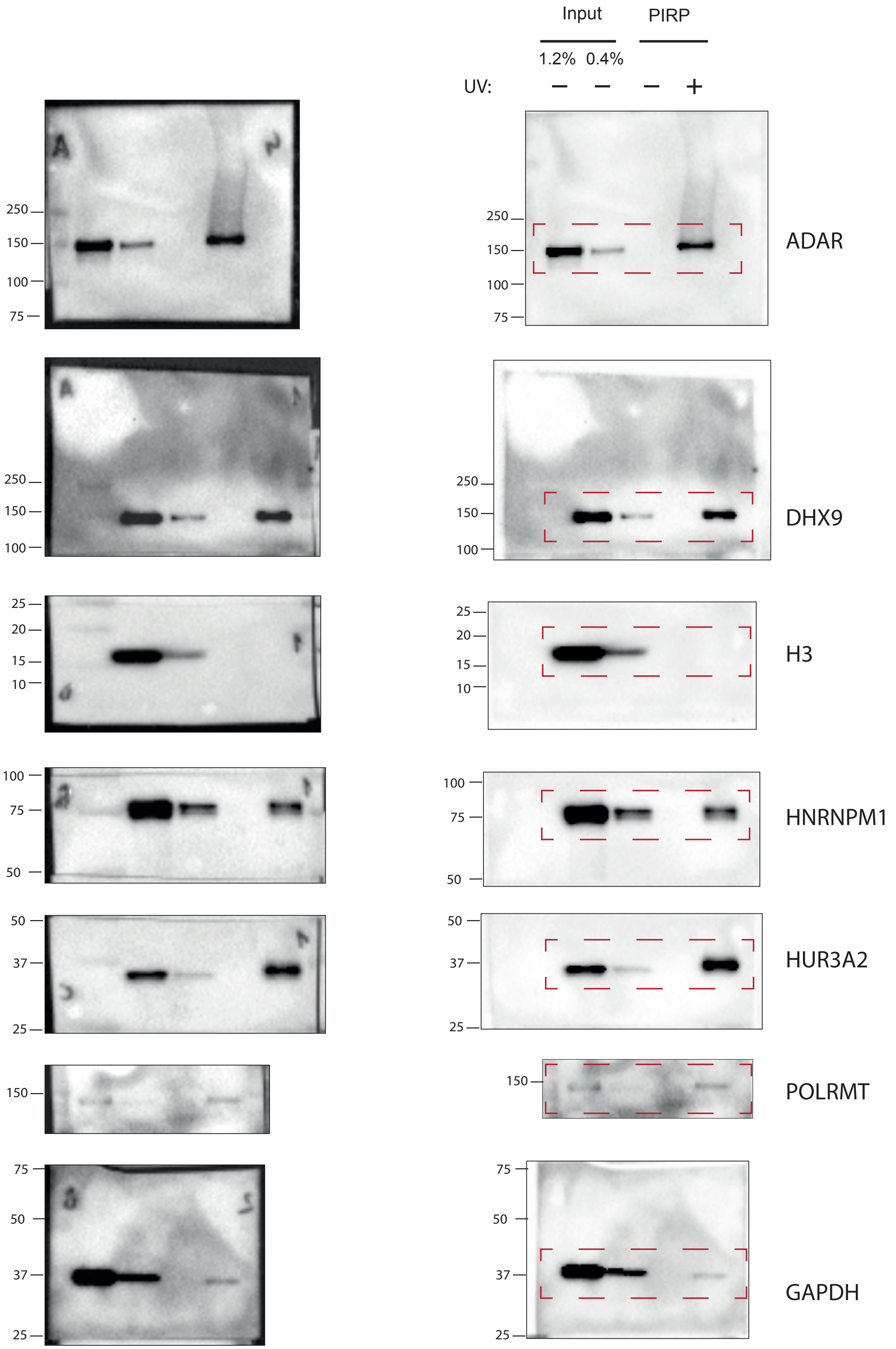

Supplementary Figure 21

Uncropped blots for Supplementary Fig. 13e

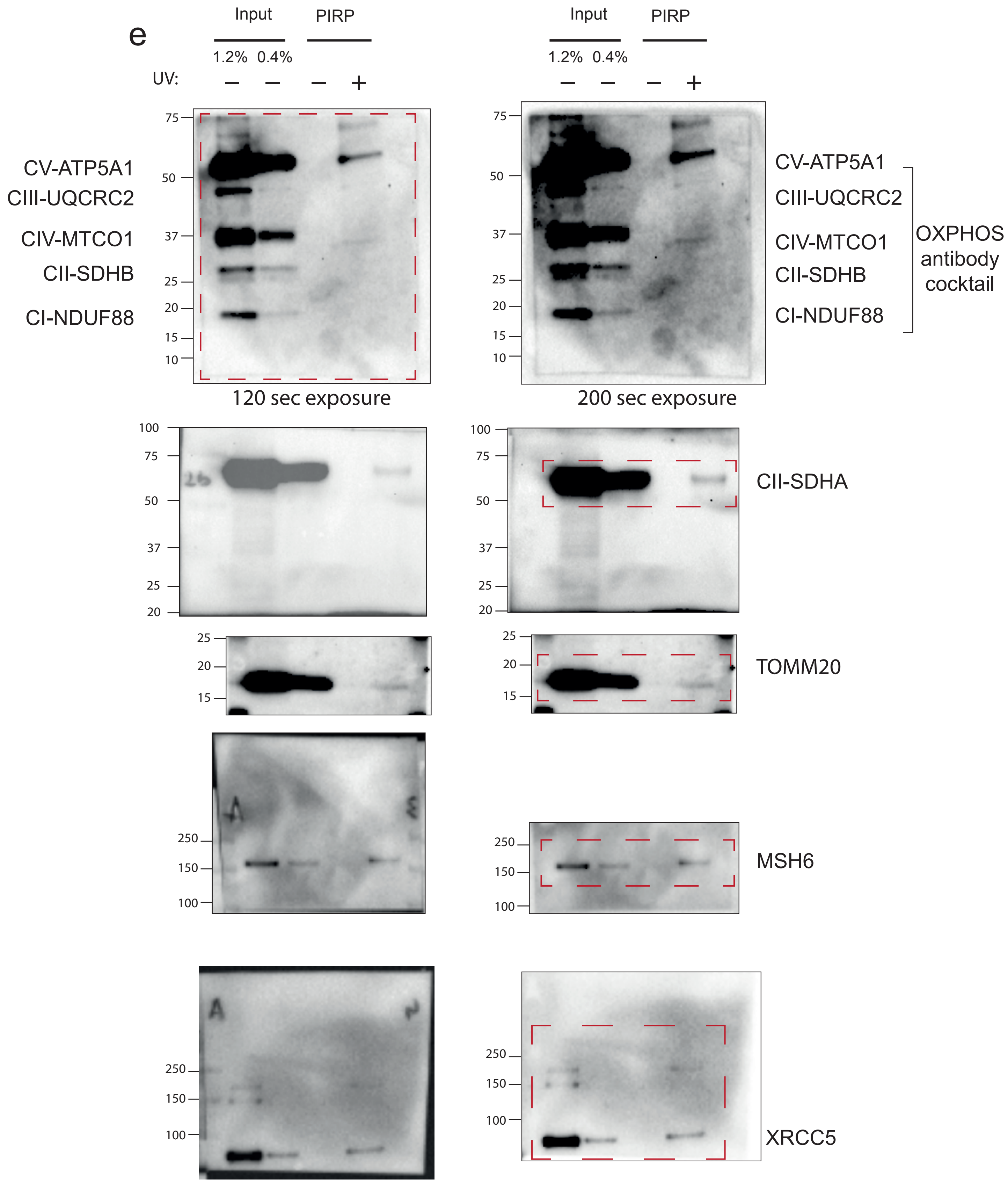

Supplementary References

1. Schwartz, J. C., Wang, X., Podell, E. R. & Cech, T. R. RNA seeds higher-order assembly of FUS protein. *Cell Rep.* **5**, 918–925 (2013).
2. Leo, G. *et al.* Ultraviolet laser-induced cross-linking in peptides. *Rapid Commun. Mass Spectrom.* **27**, 1660–1668 (2013).
3. Meisenheimer, K. M. & Koch, T. H. Photocross-linking of nucleic acids to associated proteins. *Crit. Rev. Biochem. Mol. Biol.* **32**, 101–140 (1997).
4. Budowsky, E. I. & Abdurashidova, G. G. Polynucleotide—Protein Cross-Links Induced by Ultraviolet Light and Their Use for Structural Investigation of Nucleoproteins. *Progress in Nucleic Acid Research and Molecular Biology* 1–65 (1989). doi:10.1016/s0079-6603(08)60694-7
5. Brimacombe, R., Stiege, W., Kyriatsoulis, A. & Maly, P. Intra-RNA and RNA-protein cross-linking techniques in Escherichia coli ribosomes. *Methods Enzymol.* **164**, 287–309 (1988).
6. McHugh, C. A. & Guttman, M. RAP-MS: A Method to Identify Proteins that Interact Directly with a Specific RNA Molecule in Cells. *Methods Mol. Biol.* **1649**, 473–488 (2018).
7. Urlaub, H., Hartmuth, K. & Lührmann, R. A two-tracked approach to analyze RNA-protein crosslinking sites in native, nonlabeled small nuclear ribonucleoprotein particles. *Methods* **26**, 170–181 (2002).
8. Kramer, K. *et al.* Photo-cross-linking and high-resolution mass spectrometry for assignment of RNA-binding sites in RNA-binding proteins. *Nat. Methods* **11**, 1064–1070 (2014).
9. Moore, K. S. & ’t Hoen, P. A. C. Computational approaches for the analysis of RNA–protein interactions: A primer for biologists. *J. Biol. Chem.* **294**, 1–9 (2019).
10. Sutherland, B. W., Toews, J. & Kast, J. Utility of formaldehyde cross-linking and mass spectrometry in the study of protein-protein interactions. *J. Mass Spectrom.* **43**, 699–715 (2008).
11. Srinivasa, S., Ding, X. & Kast, J. Formaldehyde cross-linking and structural proteomics:

Bridging the gap. *Methods* **89**, 91–98 (2015).

12. Alekseyenko, A. A., Gorchakov, A. A., Kharchenko, P. V. & Kuroda, M. I. Reciprocal interactions of human C10orf12 and C17orf96 with PRC2 revealed by BioTAP-XL cross-linking and affinity purification. *Proc. Natl. Acad. Sci. U. S. A.* **111**, 2488–2493 (2014).
13. Déjardin, J. & Kingston, R. E. Purification of proteins associated with specific genomic Loci. *Cell* **136**, 175–186 (2009).
14. Mohammed, H. *et al.* Rapid immunoprecipitation mass spectrometry of endogenous proteins (RIME) for analysis of chromatin complexes. *Nat. Protoc.* **11**, 316–326 (2016).
15. Liu, X. *et al.* In Situ Capture of Chromatin Interactions by Biotinylated dCas9. *Cell* **170**, 1028–1043.e19 (2017).
16. Hoffman, E. A., Frey, B. L., Smith, L. M. & Auble, D. T. Formaldehyde crosslinking: a tool for the study of chromatin complexes. *J. Biol. Chem.* **290**, 26404–26411 (2015).
17. Guerrero, C., Tagwerker, C., Kaiser, P. & Huang, L. An integrated mass spectrometry-based proteomic approach quantitative analysis of tandem affinity-purified in vivo cross-linked protein complexes (qtax) to decipher the 26 s proteasome-interacting network. *Mol. Cell. Proteomics* **5**, 366–378 (2006).
18. Tagwerker, C. *et al.* A tandem affinity tag for two-step purification under fully denaturing conditions: application in ubiquitin profiling and protein complex identification combined with in vivocross-linking. *Mol. Cell. Proteomics* **5**, 737–748 (2006).
19. Larance, M. *et al.* Global Membrane Protein Interactome Analysis using In vivo Crosslinking and Mass Spectrometry-based Protein Correlation Profiling. *Mol. Cell. Proteomics* **15**, 2476–2490 (2016).
20. Chu, C. *et al.* Systematic discovery of Xist RNA binding proteins. *Cell* **161**, 404–416 (2015).
21. Quinn, J. J. *et al.* Revealing long noncoding RNA architecture and functions using domain-specific chromatin isolation by RNA purification. *Nat. Biotechnol.* **32**, 933–940 (2014).
22. Simon, M. D. Capture Hybridization Analysis of RNA Targets (CHART). in *Current*

Protocols in Molecular Biology (2013).

23. Singh, G., Ricci, E. P. & Moore, M. J. RIPiT-Seq: a high-throughput approach for footprinting RNA:protein complexes. *Methods* **65**, 320–332 (2014).
24. Schmidt, C., Kramer, K. & Urlaub, H. Investigation of protein-RNA interactions by mass spectrometry--Techniques and applications. *J. Proteomics* **75**, 3478–3494 (2012).
25. Richter, F. M., Hsiao, H.-H., Plessmann, U. & Urlaub, H. Enrichment of protein-RNA crosslinks from crude UV-irradiated mixtures for MS analysis by on-line chromatography using titanium dioxide columns. *Biopolymers* **91**, 297–309 (2009).
26. Steen, H. & Jensen, O. N. Analysis of protein-nucleic acid interactions by photochemical cross-linking and mass spectrometry. *Mass Spectrom. Rev.* **21**, 163–182 (2002).
27. Kong, A. T., Leprevost, F. V., Avtonomov, D. M., Mellacheruvu, D. & Nesvizhskii, A. I. MSFragger: ultrafast and comprehensive peptide identification in mass spectrometry-based proteomics. *Nat. Methods* **14**, 513–520 (2017).
28. Kao, C., Vaughan, Running & Qi. Mapping protein–RNA interactions. *Virus Adaptation and Treatment* 29 (2012).
29. Oldfield, C. J. & Keith Dunker, A. Intrinsically Disordered Proteins and Intrinsically Disordered Protein Regions. *Annu. Rev. Biochem.* **83**, 553–584 (2014).
30. Bailey, T. L. *et al.* MEME SUITE: tools for motif discovery and searching. *Nucleic Acids Res.* **37**, W202–8 (2009).
31. Thandapani, P., O'Connor, T. R., Bailey, T. L. & Richard, S. Defining the RGG/RG motif. *Mol. Cell* **50**, 613–623 (2013).
32. Järvelin, A. I., Noerenberg, M., Davis, I. & Castello, A. The new (dis)order in RNA regulation. *Cell Commun. Signal.* **14**, 9 (2016).
33. Kiledjian, M. & Dreyfuss, G. Primary structure and binding activity of the hnRNP U protein: binding RNA through RGG box. *EMBO J.* **11**, 2655–2664 (1992).
34. Huang, D. W., Sherman, B. T. & Lempicki, R. A. Systematic and integrative analysis of

large gene lists using DAVID bioinformatics resources. *Nat. Protoc.* **4**, 44–57 (2009).

35. Liao, Y. *et al.* The Cardiomyocyte RNA-Binding Proteome: Links to Intermediary Metabolism and Heart Disease. *Cell Rep.* **16**, 1456–1469 (2016).
36. Castello, A. *et al.* Comprehensive Identification of RNA-Binding Domains in Human Cells. *Mol. Cell* **63**, 696–710 (2016).
37. He, C. *et al.* High-Resolution Mapping of RNA-Binding Regions in the Nuclear Proteome of Embryonic Stem Cells. *Mol. Cell* **64**, 416–430 (2016).
38. Mullari, M., Lyon, D., Jensen, L. J. & Nielsen, M. L. Specifying RNA-Binding Regions in Proteins by Peptide Cross-Linking and Affinity Purification. *J. Proteome Res.* **16**, 2762–2772 (2017).
39. Bailey, T. L. DREME: motif discovery in transcription factor ChIP-seq data. *Bioinformatics* **27**, 1653–1659 (2011).
40. Vacic, V., Uversky, V. N., Dunker, A. K. & Lonardi, S. Composition Profiler: a tool for discovery and visualization of amino acid composition differences. *BMC Bioinformatics* **8**, 211 (2007).
