# Supplementary material for: CAPRI enables comparison of evolutionarily conserved RNA interacting regions
Source: Nat Commun. 2019 Jun 18;10:2682. doi: 10.1038/s41467-019-10585-3 (PMC6581911; doi:10.1038/s41467-019-10585-3)
Supplement: Supplementary file 3 — Description of Additional Supplementary Files [file 41467_2019_10585_MOESM3_ESM.docx]

**Description of Additional Supplementary Files**

**File Name: Supplementary Data 1**

**Description:** Drosophila UV and FA RBPome with protein intensities protein-groups table.

**File Name: Supplementary Data 2**

**Description:** Drosophila adjacent peptides identified by MaxQuant and ADJ-peptides identified after in silico extension of adjacent peptides.

**File Name: Supplementary Data 3**

**Description:** Human adjacent peptides identified by MaxQuant and ADJ-peptides identified after in silico extension of adjacent peptides.

**File Name: Supplementary Data 4**

**Description:** Monoisotopic masses used to define RNA-PTMs and masses used to define RNA marker ions and neutral losses. Comparison of spectra identified in SS1, SS2 and SS3 in Drosophila XL-peptide analysis.

**File Name: Supplementary Data 5**

**Description:** This file represents a gallery of examples showing XL-peptides with diverse crosslinks in human and Drosophila.

**File Name: Supplementary Data 6**

**Description:** This file represents a gallery of examples showing crosslinked peptides and ADJ-peptides mapped to PDB structures in humans.

**File Name: Supplementary Data 7**

**Description:** This file represents a gallery of examples of crosslinked peptides identifying novel domains in Drosophila and humans.

**File Name: Supplementary Data 8**

**Description:** Drosophila XL-peptides and information about Pfam domains and disordered regions.

**File Name: Supplementary Data 9**

**Description:** Human XL-peptides and information about Pfam domains and disordered regions.

**File Name: Supplementary Data 10**

**Description:** Human and Drosophila CAPRI (ADJ- and XL-peptides together) with information about overlaps with Pfam domains.

**File Name: Supplementary Data 11**

**Description:** Drosophila FA-dom-peptides and information about Pfam domains and disordered regions.

**File Name: Supplementary Data 12**

**Description:** Human and Drosophila orthologous protein pairs with information about domains identified in each of them. All novel conserved domains identified in both Drosophila and humans.

**File Name: Supplementary Data 13**

**Description:** New RNA binding Pfam domains identified in Drosophila.
