# Supplementary material for: CAPRI enables comparison of evolutionarily conserved RNA interacting regions
Source: Nat Commun. 2019 Jun 18;10:2682. doi: 10.1038/s41467-019-10585-3 (PMC6581911; doi:10.1038/s41467-019-10585-3)
Supplement: Supplementary file 8 — Supplementary Data 5 [file 41467_2019_10585_MOESM8_ESM.pdf]

Supplementary Table 5

XL-peptide with Single nucleotide

1)

Name	Sequence	RNA PTMs	M/Z	z	Position of XL
RPS2	T(+212.01)YSY(+112.03)LTPDLWK	U	855.8701	2	4Y

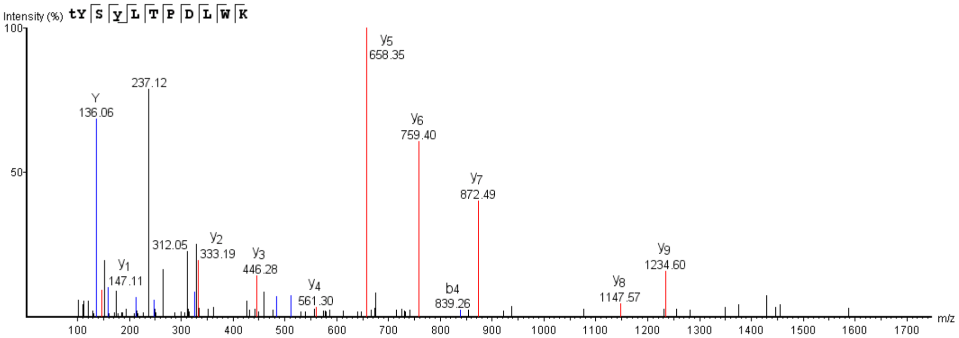

#	Immonium	b	b (2+)	Seq	y	y (2+)	#
1	286.07	314.06	157.53	T(+212.01)			11
2	136.06	477.13	239.06	Y	1397.67	699.34	10
3	60.04	564.16	282.58	S	1234.60	617.81	9
4	248.10	839.26	420.13	Y(+112.03)	1147.57	574.29	8
5	86.10	952.33	476.67	L	872.49	436.74	7
6	74.06	1053.38	527.19	T	759.40	380.20	6
7	70.07	1150.43	575.72	P	658.35	329.68	5
8	88.04	1265.46	633.23	D	561.30	281.15	4
9	86.10	1378.55	689.77	L	446.28	223.64	3
10	159.09	1564.62	782.81	W	333.19	167.10	2
11	101.11			K	147.11	74.06	1

2)

Name	Sequence	RNA PTMs	M/Z	z	Position of XL
RS5	T(+324.03)IAEC(+57.02)LADELINAAK	U	969.4324	2	TI

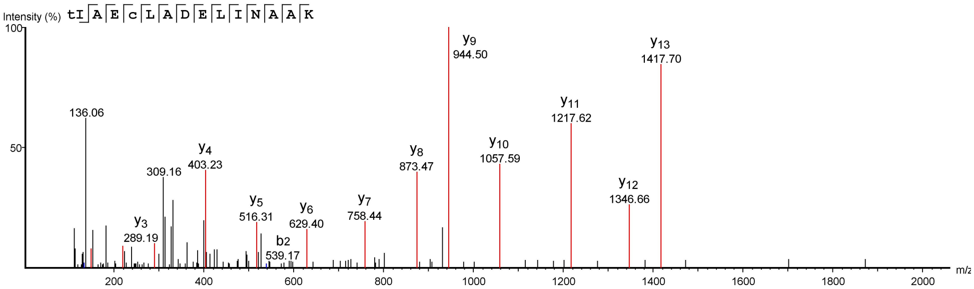

#	Immonium	b	b (2+)	Seq	y	y (2+)	#
1	398.10	426.09	213.55	T(+324.04)			15
2	86.10	539.17	270.09	I	1530.78	765.89	14
3	44.05	610.21	305.61	A	1417.70	709.35	13
4	102.06	739.26	370.13	E	1346.66	673.83	12
5	133.04	899.29	450.14	C(+57.02)	1217.62	609.31	11
6	86.10	1012.37	506.68	L	1057.59	529.29	10
7	44.05	1083.41	542.20	A	944.50	472.75	9
8	88.04	1198.43	599.72	D	873.47	437.23	8
9	102.06	1327.48	664.24	E	758.44	379.72	7
10	86.10	1440.56	720.78	L	629.40	315.20	6
11	86.10	1553.64	777.32	I	516.31	258.66	5
12	87.06	1667.69	834.34	N	403.23	202.11	4
13	44.05	1738.72	869.86	A	289.19	145.09	3
14	44.05	1809.76	905.38	A	218.15	109.57	2
15	101.11			K	147.11	74.06	1

3)

Name	Sequence	RNA PTMs	M/Z	z	Position of XL
NONO	L(+324.04)FVGNLPADITEDEFK	U	1092.4796	2	Via base to 1L

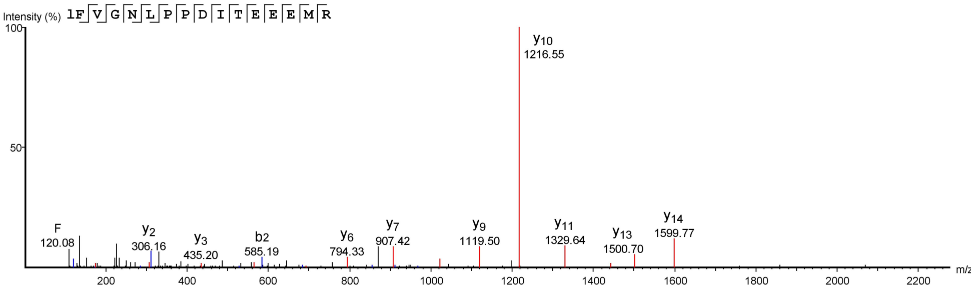

#	Immonium	b	b (2+)	Seq	y	y (2+)	#
1	410.13	438.13	219.56	L(+324.04)			16
2	120.08	585.19	293.10	F	1746.84	873.92	15
3	72.08	684.27	342.63	V	1599.77	800.38	14
4	30.03	741.29	371.14	G	1500.70	750.85	13
5	87.06	855.33	428.16	N	1443.67	722.34	12
6	86.10	968.41	484.71	L	1329.64	665.32	11
7	70.07	1065.47	533.23	P	1216.55	608.78	10
8	70.07	1162.52	581.76	P	1119.50	560.25	9
9	88.04	1277.55	639.27	D	1022.44	511.72	8
10	86.10	1390.63	695.81	I	907.42	454.21	7
11	74.06	1491.68	746.34	T	794.33	397.67	6
12	102.06	1620.72	810.86	E	693.29	347.14	5
13	102.06	1749.76	875.38	E	564.25	282.62	4
14	102.06	1878.80	939.90	E	435.20	218.10	3
15	104.05	2009.85	1005.42	M	306.16	153.58	2
16	129.11			R	175.12	88.06	1

4)

Name	Sequence	RNA PTMs	M/Z	z	Position of XL
SFPQ	L(+324.04)FVGNLPADITEDEFK	U	1066.4806	2	Via base to L

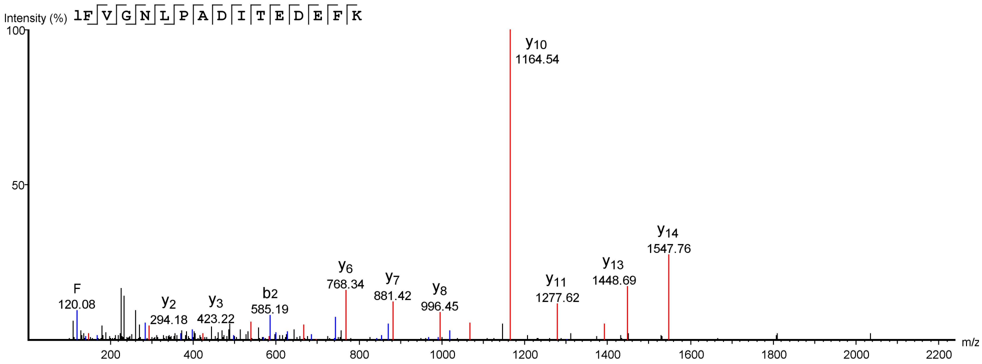

#	Immonium	b	b (2+)	Seq	y	y (2+)	#
1	410.13	438.13	219.56	L(+324.04)			16
2	120.08	585.19	293.10	F	1694.83	847.91	15
3	72.08	684.26	342.63	V	1547.76	774.38	14
4	30.03	741.28	371.14	G	1448.69	724.85	13
5	87.06	855.33	428.16	N	1391.66	696.32	12
6	86.10	968.41	484.71	L	1277.62	639.31	11
7	70.07	1065.47	533.23	P	1164.54	582.77	10
8	44.05	1136.50	568.75	A	1067.49	534.24	9
9	88.04	1251.53	626.26	D	996.45	498.73	8
10	86.10	1364.61	682.81	I	881.42	441.21	7
11	74.06	1465.66	733.33	T	768.34	384.67	6
12	102.06	1594.70	797.85	E	667.29	334.15	5
13	88.04	1709.73	855.37	D	538.25	269.63	4
14	102.06	1838.77	919.89	E	423.22	212.11	3
15	120.08	1985.84	993.42	F	294.18	147.59	2
16	101.11			K	147.11	74.06	1

5)

Name	Sequence	RNA PTMs	M/Z	z	Position of XL
CELF2	M(+324.04)FVGQIPR	U	636.2780	2	N.D.

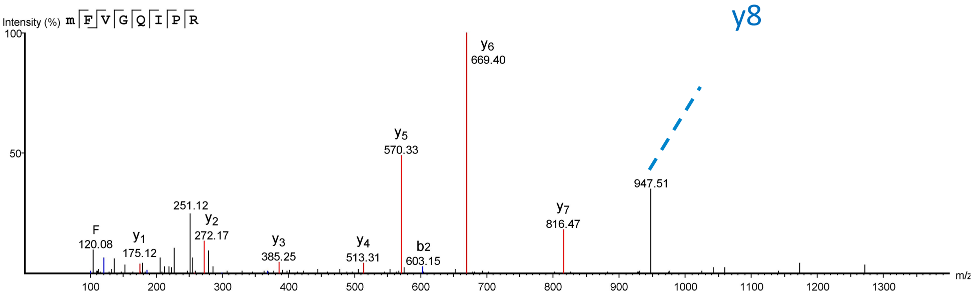

#	Immonium	b	b (2+)	Seq	y	y (2+)	#
1	428.09	456.08	228.54	M(+324.04)			8
2	120.08	603.15	302.08	F	816.47	408.74	7
3	72.08	702.22	351.61	V	669.40	335.20	6
4	30.03	759.24	380.12	G	570.33	285.67	5
5	101.07	887.30	444.15	Q	513.31	257.16	4
6	86.10	1000.39	500.69	I	385.25	193.13	3
7	70.07	1097.44	549.22	P	272.17	136.59	2
8	129.11			R	175.12	88.06	1

6)

Name	Sequence	RNA PTMs	M/Z	z	Position of XL
YBOX1	EDVFHQTAIK(+323.05)	C	805.3361	2	K or C-term

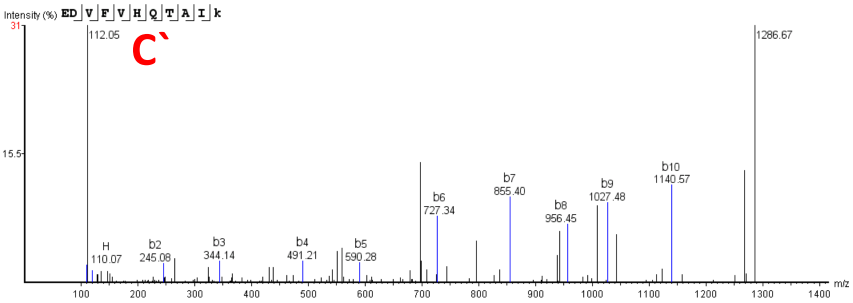

#	Immonium	b	b (2+)	Seq	y	y (2+)	#
1	102.06	130.05	65.53	E			11
2	88.04	245.08	123.04	D	1480.68	740.84	10
3	72.08	344.14	172.57	V	1365.66	683.33	9
4	120.08	491.21	246.11	F	1266.59	633.79	8
5	72.08	590.28	295.64	V	1119.52	560.26	7
6	110.07	727.34	364.17	H	1020.45	510.73	6
7	101.07	855.40	428.20	Q	883.39	442.20	5
8	74.06	956.45	478.72	T	755.33	378.17	4
9	44.05	1027.48	514.24	A	654.29	327.64	3
10	86.10	1140.57	570.78	I	583.25	292.12	2
11	424.16			K(+323.05)	470.16	235.58	1

7)

Name	Sequence	RNA PTMs	M/Z	z	Position of XL
YBOX1	E(+212.01)D(+111.04)VFHQTAIK	C	537.2469	3	D

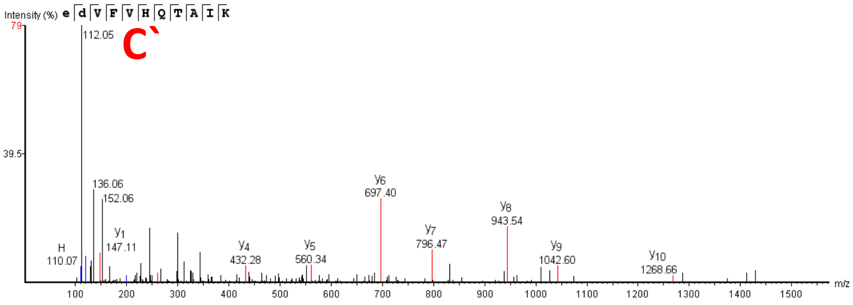

#	Immonium	b	b (2+)	Seq	y	y (2+)	#
1	314.06	342.06	171.53	E(+212.01)			11
2	199.07	568.13	284.56	D(+111.04)	1268.66	634.84	10
3	72.08	667.20	334.10	V	1042.60	521.80	9
4	120.08	814.27	407.63	F	943.54	472.27	8
5	72.08	913.33	457.17	V	796.47	398.73	7
6	110.07	1050.39	525.70	H	697.40	349.20	6
7	101.07	1178.45	589.73	Q	560.34	280.67	5
8	74.06	1279.50	640.25	T	432.28	216.64	4
9	44.05	1350.54	675.77	A	331.23	166.12	3
10	86.10	1463.62	732.31	I	260.20	130.60	2
11	101.11			K	147.11	74.06	1

8)

Name	Sequence	RNA PTMs	M/Z	z	Position of XL
NCL	V(+323.05)EGTEPTTAFNLFVGNLNFNK	C	879.0763	3	N.D.

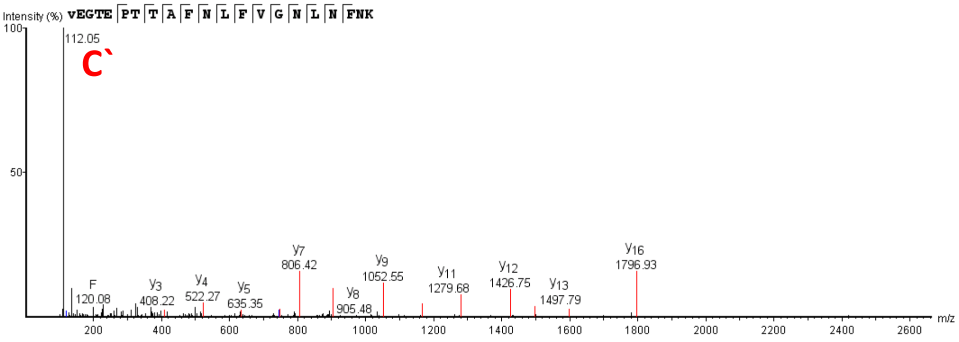

#	Immonium	b	b (2+)	Seq	y	y (2+)	#
1	395.13	423.13	212.06	V(+323.05)			21
2	102.06	552.17	276.59	E	2213.09	1107.04	20
3	30.03	609.19	305.10	G	2084.04	1042.52	19
4	74.06	710.24	355.62	T	2027.02	1014.01	18
5	102.06	839.28	420.14	E	1925.98	963.49	17
6	70.07	936.34	468.67	P	1796.93	898.97	16
7	74.06	1037.38	519.19	T	1699.88	850.44	15
8	74.06	1138.43	569.72	T	1598.83	799.92	14
9	44.05	1209.47	605.23	A	1497.79	749.40	13
10	120.08	1356.54	678.77	F	1426.75	713.87	12
11	87.06	1470.58	735.79	N	1279.68	640.34	11
12	86.10	1583.66	792.33	L	1165.63	583.32	10
13	120.08	1730.73	865.87	F	1052.55	526.78	9
14	72.08	1829.80	915.40	V	905.48	453.24	8
15	30.03	1886.82	943.91	G	806.42	403.71	7
16	87.06	2000.86	1000.93	N	749.40	375.20	6
17	86.10	2113.95	1057.47	L	635.35	318.18	5
18	87.06	2227.99	1114.50	N	522.27	261.63	4
19	120.08	2375.06	1188.03	F	408.22	204.61	3
20	87.06	2489.10	1245.05	N	261.16	131.08	2
21	101.11			K	147.11	74.06	1

XL-peptide with Single nucleotide H_2O loss

9)

Name	Sequence	RNA PTMs	M/Z	z	Position of XL
NCL	V(+306.03)EGTEPTTAFNLFVGNLNFNK	U-H2O	1309.5936	2	N.D.

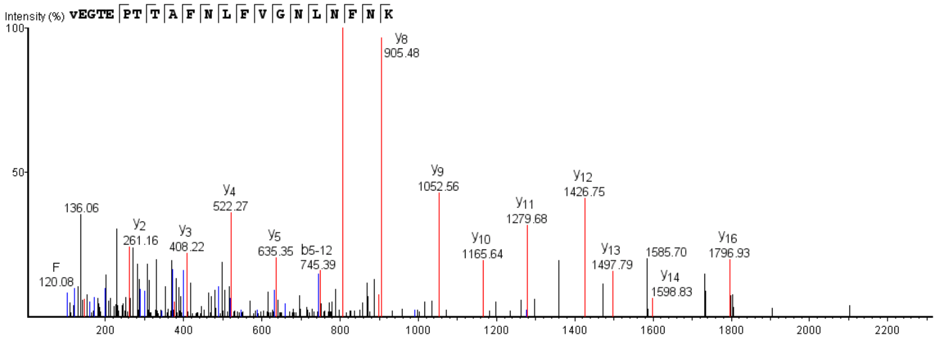

#	Immonium	b	b (2+)	Seq	y	y (2+)	#
1	378.11	406.10	203.55	V(+306.03)			21
2	102.06	535.14	268.07	E	2213.09	1107.04	20
3	30.03	592.17	296.58	G	2084.04	1042.52	19
4	74.06	693.21	347.11	T	2027.02	1014.01	18
5	102.06	822.26	411.63	E	1925.98	963.49	17
6	70.07	919.31	460.15	P	1796.93	898.97	16
7	74.06	1020.36	510.68	T	1699.88	850.44	15
8	74.06	1121.40	561.20	T	1598.83	799.92	14
9	44.05	1192.44	596.72	A	1497.79	749.39	13
10	120.08	1339.51	670.25	F	1426.75	713.87	12
11	87.06	1453.55	727.28	N	1279.68	640.34	11
12	86.10	1566.64	783.82	L	1165.64	583.32	10
13	120.08	1713.70	857.35	F	1052.56	526.78	9
14	72.08	1812.77	906.89	V	905.48	453.24	8
15	30.03	1869.79	935.40	G	806.42	403.71	7
16	87.06	1983.84	992.42	N	749.39	375.20	6
17	86.10	2096.92	1048.96	L	635.35	318.18	5
18	87.06	2210.96	1105.98	N	522.27	261.63	4
19	120.08	2358.03	1179.52	F	408.22	204.61	3
20	87.06	2472.08	1236.54	N	261.16	131.08	2
21	101.11			K	147.11	74.06	1

10)

Name	Sequence	RNA PTMs	M/Z	z	Position of XL
RS5	T(+306.03)IAEC(+57.02)LADELINAAK	U-H2O	969.4324	2	T

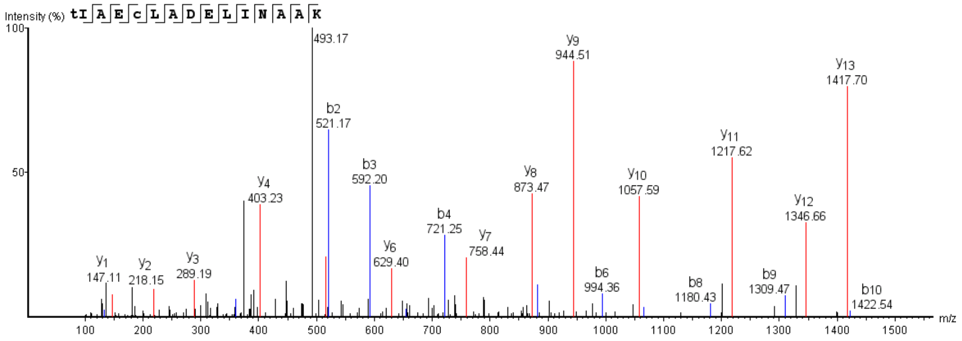

#	Immonium	b	b (2+)	Seq	y	y (2+)	#
1	380.09	408.08	204.54	T(+306.03)			15
2	86.10	521.17	261.08	I	1530.78	765.89	14
3	44.05	592.20	296.60	A	1417.70	709.35	13
4	102.06	721.25	361.12	E	1346.66	673.83	12
5	133.04	881.27	441.14	C(+57.02)	1217.62	609.31	11
6	86.10	994.36	497.68	L	1057.59	529.29	10
7	44.05	1065.39	533.20	A	944.51	472.75	9
8	88.04	1180.43	590.71	D	873.47	437.23	8
9	102.06	1309.47	655.24	E	758.44	379.72	7
10	86.10	1422.54	711.77	L	629.40	315.20	6
11	86.10	1535.63	768.32	I	516.32	258.66	5
12	87.06	1649.68	825.34	N	403.23	202.11	4
13	44.05	1720.71	860.86	A	289.19	145.09	3
14	44.05	1791.75	896.38	A	218.15	109.57	2
15	101.11			K	147.11	74.06	1

11)

Name	Sequence	RNA PTMs	M/Z	z	Position of XL
CELF2	MFVGQI(+112.03)PR(+194.00)	U-H2O	627.2778	2	Via base to I

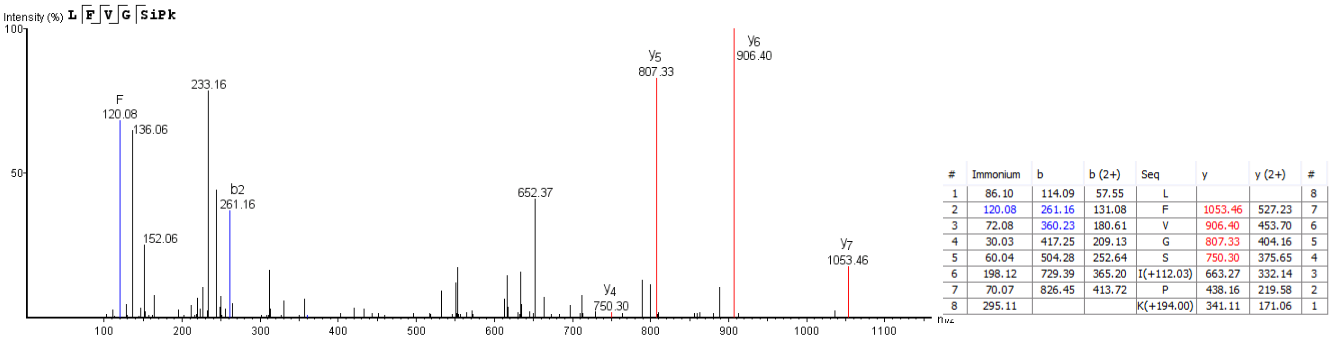

#	Immonium	b	b (2+)	Seq	y	y (2+)	#
1	86.10	114.09	57.55	L			8
2	120.08	261.16	131.08	F	1053.46	527.23	7
3	72.08	360.23	180.61	V	906.40	453.70	6
4	30.03	417.25	209.13	G	807.33	404.16	5
5	60.04	504.28	252.64	S	750.30	375.65	4
6	198.12	729.39	365.20	I(+112.03)	663.27	332.14	3
7	70.07	826.45	413.72	P	438.16	219.58	2
8	295.11			K(+194.00)	341.11	171.06	1

12)

Name	Sequence	RNA PTMs	M/Z	z	Position of XL
SFPQ	C(+57.02)(+306.03)RLFVGNLPADITEDEFK	U-H2O	1215.539	2	CRFLV

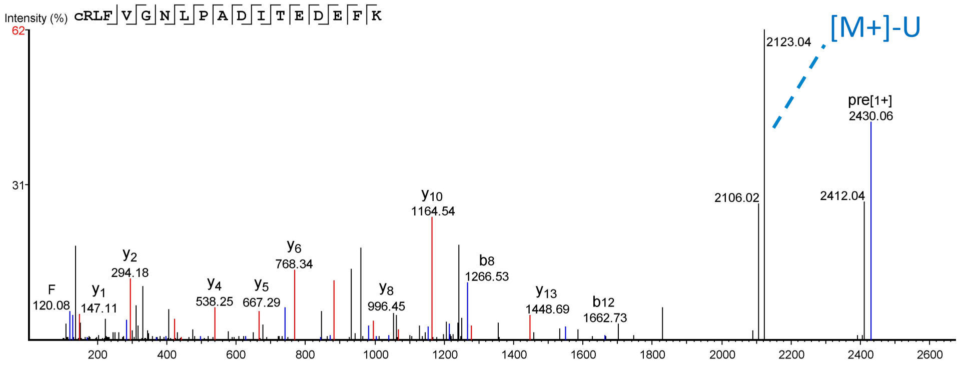

#	Immonium	b	b (2+)	Seq	y	y (2+)	#
1	439.08	467.07	234.04	C(+363.06)			18
2	129.10	623.18	312.09	R	1964.01	982.51	17
3	86.10	736.26	368.63	L	1807.91	904.46	16
4	120.08	883.32	442.16	F	1694.83	847.91	15
5	72.08	982.38	491.70	V	1547.76	774.38	14
6	30.03	1039.40	520.21	G	1448.69	724.85	13
7	87.06	1153.45	577.23	N	1391.67	696.33	12
8	86.10	1266.53	633.77	L	1277.62	639.31	11
9	70.07	1363.60	682.30	P	1164.54	582.77	10
10	44.05	1434.64	717.82	A	1067.49	534.24	9
11	88.04	1549.64	775.33	D	996.45	498.73	8
12	86.10	1662.73	831.87	I	881.42	441.20	7
13	74.06	1763.79	882.40	T	768.34	384.67	6
14	102.06	1892.84	946.92	E	667.29	334.15	5
15	88.04	2007.86	1004.42	D	538.25	269.63	4
16	102.06	2136.91	1068.95	E	423.22	212.11	3
17	120.08	2283.97	1142.49	F	294.18	147.59	2
18	101.11			K	147.11	74.06	1

13)

Name	Sequence	RNA PTMs	M/Z	z	Position of XL
YBOX1	E(+306.03)DVFHQTAIK	U-H2O	796.8436	2	E

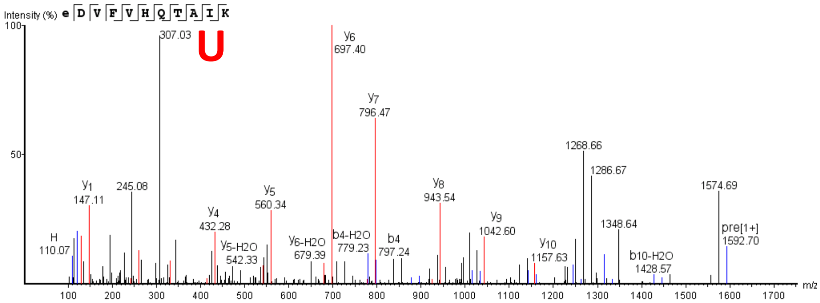

#	Immonium	b	b (2+)	Seq	y	y (2+)	#
1	408.08	436.08	218.54	E(+306.03)			11
2	88.04	551.10	276.05	D	1157.63	579.32	10
3	72.08	650.17	325.59	V	1042.60	521.80	9
4	120.08	797.24	399.12	F	943.54	472.27	8
5	72.08	896.31	448.65	V	796.47	398.73	7
6	110.07	1033.37	517.18	H	697.40	349.20	6
7	101.07	1161.42	581.21	Q	560.34	280.67	5
8	74.06	1262.47	631.74	T	432.28	216.64	4
9	44.05	1333.51	667.26	A	331.23	166.12	3
10	86.10	1446.59	723.80	I	260.20	130.60	2
11	101.11			K	147.11	74.06	1

XL-peptide with base alone

14)

Name	Sequence	RNA PTMs	M/Z	z	Position of XL
FUBP2	C(+112.03)GLVIGR	U`	415.2213	2	Via base to C

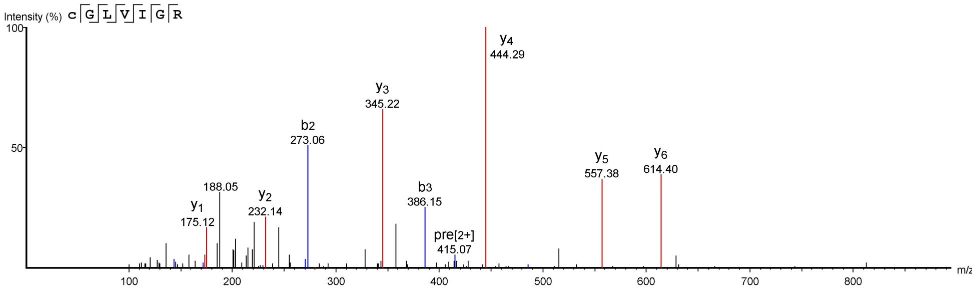

#	Immonium	b	b (2+)	Seq	y	y (2+)	#
1	188.05	216.04	108.52	C(+112.03)			7
2	30.03	273.06	137.03	G	614.40	307.70	6
3	86.10	386.15	193.57	L	557.38	279.19	5
4	72.08	485.22	243.11	V	444.29	222.65	4
5	86.10	598.30	299.65	I	345.22	173.13	3
6	30.03	655.32	328.16	G	232.14	116.57	2
7	129.11			R	175.12	88.06	1

XL-peptide with base alone

15)

Name	Sequence	RNA PTMs	M/Z	z	Position of XL
SRSF2	S(+42.01)YGRP(+151.05)PPDVEGMTSLK	G`	642.9759	3	Via base to P

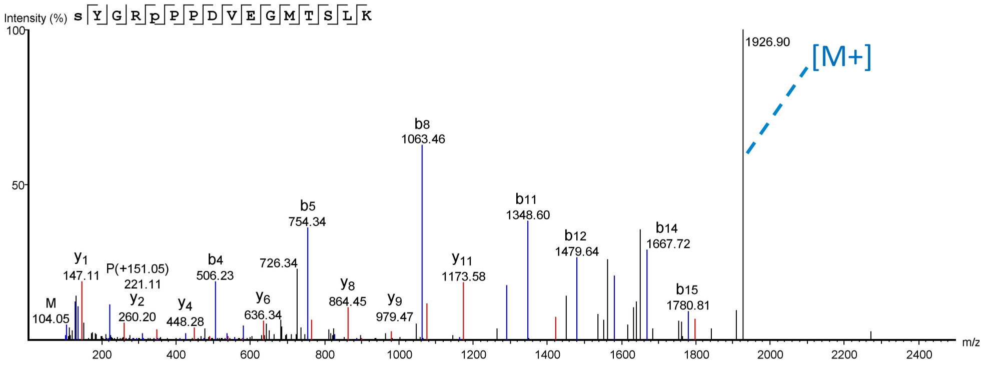

#	Immonium	b	b (2+)	Seq	y	y (2+)	#
1	102.06	130.05	65.53	S(+42.01)			16
2	136.08	293.11	147.06	Y	1797.86	899.42	15
3	30.03	350.13	175.57	G	1634.81	817.90	14
4	129.10	506.23	253.62	R	1577.79	789.39	13
5	221.11	754.34	377.67	P(+151.05)	1421.68	711.34	12
6	70.07	851.39	426.20	P	1173.58	587.29	11
7	70.07	948.44	474.72	P	1076.53	538.76	10
8	88.04	1063.46	532.24	D	979.47	490.24	9
9	72.08	1162.54	581.77	V	864.45	432.72	8
10	102.05	1291.58	646.29	E	765.38	383.19	7
11	30.03	1348.60	674.80	G	636.34	318.67	6
12	104.05	1479.64	740.32	M	579.32	290.16	5
13	74.06	1580.68	790.85	T	448.28	224.64	4
14	60.04	1667.72	834.36	S	347.23	174.11	3
15	86.10	1780.81	890.90	L	260.20	130.60	2
16	101.11			K	147.11	74.06	1

Category: XL-peptides with two nucleotides

16)

Name	Sequence	RNA PTMs	M/Z	z	Position of XL
RPS2	TY(+518.03)SY(+112.03)LTPDLWK	UU	1008.8829	2	4Y

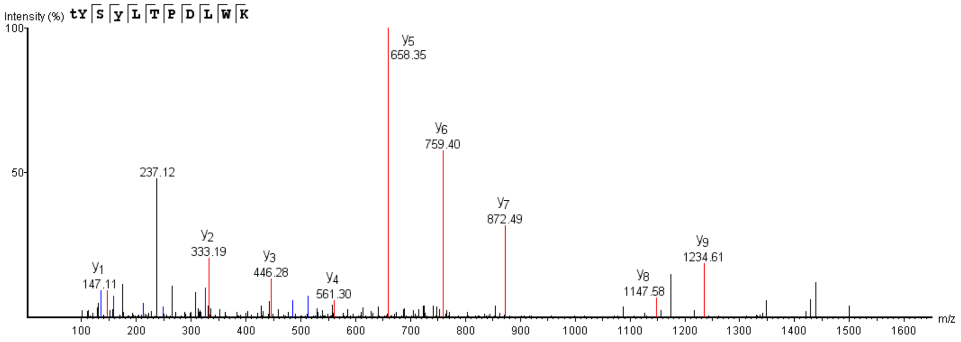

#	Immonium	b	b (2+)	Seq	y	y (2+)	#
1	592.09	620.09	310.54	T(+518.03)			11
2	136.06	783.15	392.08	Y	1397.67	699.34	10
3	60.04	870.18	435.59	S	1234.61	617.81	9
4	248.10	1145.28	573.14	Y(+112.03)	1147.58	574.29	8
5	86.10	1258.36	629.68	L	872.49	436.74	7
6	74.06	1359.41	680.20	T	759.40	380.20	6
7	70.07	1456.46	728.73	P	658.35	329.68	5
8	88.04	1571.49	786.24	D	561.30	281.15	4
9	86.10	1684.57	842.79	L	446.28	223.64	3
10	159.09	1870.65	935.83	W	333.19	167.10	2
11	101.11			K	147.11	74.06	1

17)

Name	Sequence	RNA PTMs	M/Z	z	Position of XL
RPS2	TY(+518.03)S(+112.03)YLTPDLWK	UU	1008.8848	2	Via base to 2Y + 3S

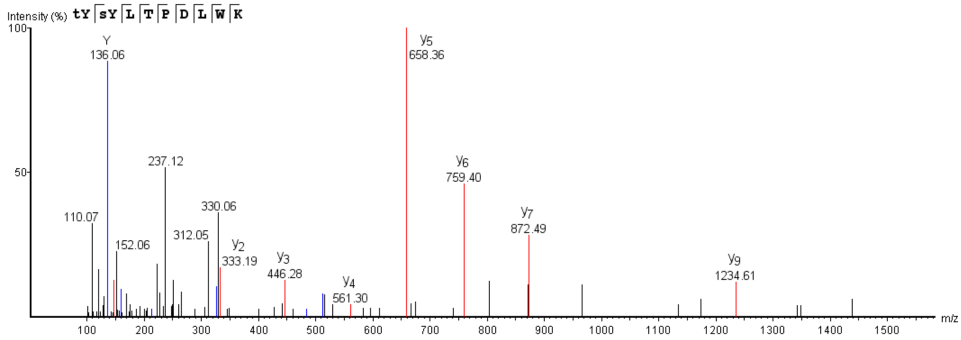

#	Immonium	b	b (2+)	Seq	y	y (2+)	#
1	592.09	620.09	310.54	T(+518.03)			11
2	136.06	783.15	392.08	Y	1397.67	699.34	10
3	172.07	982.21	491.61	S(+112.03)	1234.61	617.81	9
4	136.08	1145.28	573.14	Y	1035.55	518.28	8
5	86.10	1258.36	629.68	L	872.49	436.74	7
6	74.06	1359.41	680.20	T	759.40	380.20	6
7	70.07	1456.46	728.73	P	658.36	329.68	5
8	88.04	1571.49	786.24	D	561.30	281.15	4
9	86.10	1684.57	842.79	L	446.28	223.64	3
10	159.09	1870.65	935.83	W	333.19	167.10	2
11	101.11			K	147.11	74.06	1

18)

Name	Sequence	RNA PTMs	M/Z	z	Position of XL
NCL	V(+652.10)EGTEPTTAFNLFVGNLNFNK	CA	988.7565	3	N.D.

#	Immonium	b	b (2+)	Seq	y	y (2+)	#
1	724.19	752.18	376.59	V(+652.10)			21
2	102.06	881.22	441.11	E	2213.09	1107.04	20
3	30.03	938.24	469.62	G	2084.04	1042.52	19
4	74.06	1039.29	520.15	T	2027.02	1014.01	18
5	102.06	1168.33	584.67	E	1925.98	963.49	17
6	70.07	1265.39	633.19	P	1796.92	898.97	16
7	74.06	1366.44	683.72	T	1699.88	850.44	15
8	74.06	1467.48	734.24	T	1598.81	799.92	14
9	44.05	1538.52	769.76	A	1497.79	749.39	13
10	120.08	1685.59	843.29	F	1426.74	713.87	12
11	87.06	1799.63	900.32	N	1279.67	640.34	11
12	86.10	1912.72	956.86	L	1165.64	583.32	10
13	120.08	2059.78	1030.39	F	1052.55	526.78	9
14	72.08	2158.85	1079.93	V	905.48	453.24	8
15	30.03	2215.87	1108.44	G	806.42	403.71	7
16	87.06	2329.92	1165.46	N	749.39	375.20	6
17	86.10	2443.00	1222.00	L	635.35	318.18	5
18	87.06	2557.04	1279.02	N	522.27	261.63	4
19	120.08	2704.11	1352.56	F	408.22	204.61	3
20	87.06	2818.16	1409.58	N	261.16	131.08	2
21	101.11			K	147.11	74.06	1

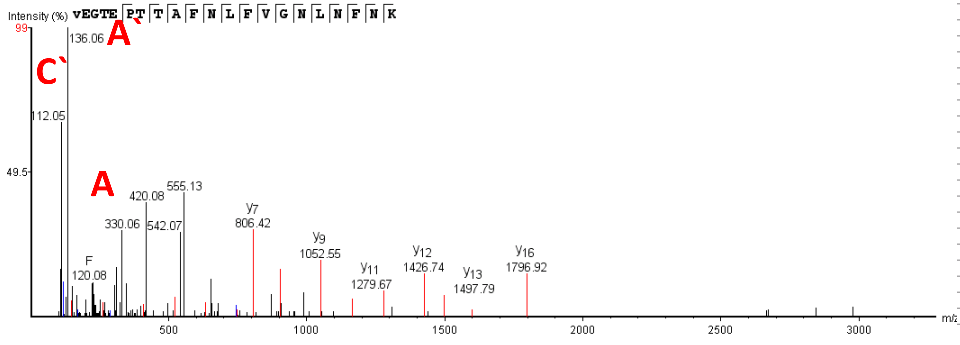

19)

Name	Sequence	RNA PTMs	M/Z	z	Position of XL
NCL	V(+668.10)EGTEPTTAFNLFVGNLNFNK	CG	994.0901	3	N.D.

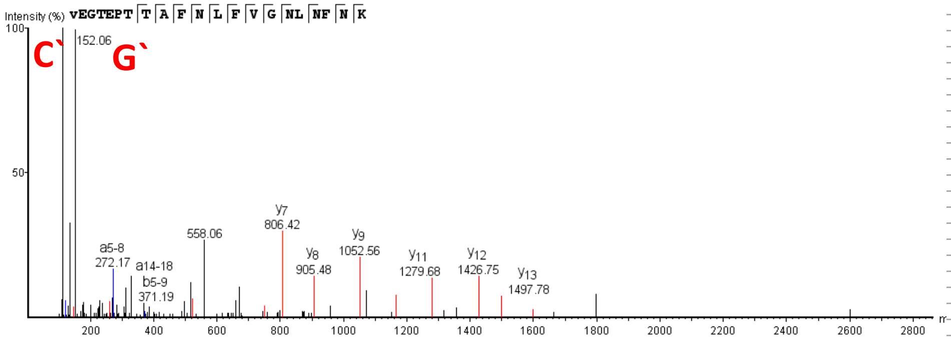

#	Immonium	b	b (2+)	Seq	y	y (2+)	#
1	740.18	768.18	384.59	V(+668.10)			21
2	102.06	897.22	449.11	E	2213.09	1107.04	20
3	30.03	954.24	477.62	G	2084.04	1042.52	19
4	74.06	1055.29	528.14	T	2027.02	1014.01	18
5	102.06	1184.33	592.66	E	1925.98	963.49	17
6	70.07	1281.38	641.19	P	1796.93	898.97	16
7	74.06	1382.43	691.72	T	1699.88	850.44	15
8	74.06	1483.48	742.24	T	1598.82	799.92	14
9	44.05	1554.52	777.76	A	1497.78	749.39	13
10	120.08	1701.58	851.29	F	1426.75	713.87	12
11	87.06	1815.63	908.31	N	1279.68	640.34	11
12	86.10	1928.71	964.86	L	1165.63	583.32	10
13	120.08	2075.78	1038.39	F	1052.56	526.78	9
14	72.08	2174.85	1087.92	V	905.48	453.24	8
15	30.03	2231.87	1116.43	G	806.42	403.71	7
16	87.06	2345.91	1173.46	N	749.39	375.20	6
17	86.10	2459.00	1230.00	L	635.35	318.18	5
18	87.06	2573.04	1287.02	N	522.27	261.63	4
19	120.08	2720.11	1360.55	F	408.22	204.61	3
20	87.06	2834.15	1417.57	N	261.16	131.08	2
21	101.11			K	147.11	74.06	1

20)

Name	Sequence	RNA PTMs	M/Z	z	Position of XL
**THOC4**	L(+517.05)LVSNLDFG(+151.05)VSDADIQELFAEFGTLK	CG	1170.5234	3	Via base to GV

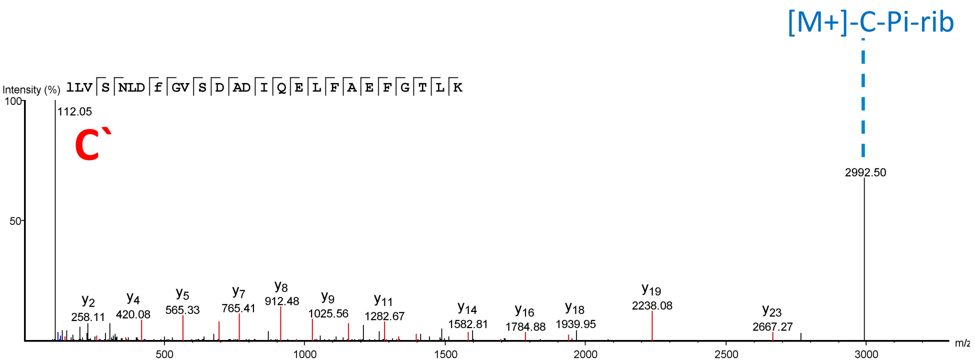

#	Immonium	b	b (2+)	Seq	y	y (2+)	#
1	603.15	631.14	316.07	L(+517.05)			26
2	86.10	744.23	372.61	L	2879.42	1440.21	25
3	72.08	843.29	422.15	V	2766.34	1383.67	24
4	60.04	930.33	465.66	S	2667.27	1334.12	23
5	87.06	1044.37	522.68	N	2580.24	1290.62	22
6	86.10	1157.45	579.23	L	2466.19	1233.60	21
7	88.04	1272.48	636.74	D	2353.11	1177.05	20
8	271.13	1570.60	785.80	F(+151.05)	2238.08	1119.54	19
9	30.03	1627.62	814.31	G	1939.95	970.50	18
10	72.08	1726.69	863.84	V	1882.94	941.97	17
11	60.04	1813.72	907.36	S	1783.86	892.44	16
12	88.04	1928.75	964.87	D	1696.84	848.92	15
13	44.05	1999.78	1000.39	A	1581.82	791.41	14
14	88.04	2114.81	1057.91	D	1510.78	755.89	13
15	86.10	2227.90	1114.45	I	1395.75	698.38	12
16	101.07	2355.95	1178.48	Q	1282.67	641.83	11
17	102.06	2485.00	1243.00	E	1154.61	577.80	10
18	86.10	2598.08	1299.54	L	1025.56	513.28	9
19	120.08	2745.15	1373.07	F	912.48	456.74	8
20	44.05	2816.19	1408.59	A	765.41	383.21	7
21	102.06	2945.23	1473.11	E	694.38	347.69	6
22	120.08	3092.30	1546.65	F	565.33	283.17	5
23	30.03	3149.32	1575.16	G	418.27	209.63	4
24	74.06	3250.37	1625.68	T	361.24	181.12	3
25	86.10	3363.45	1682.22	L	260.20	130.60	2
26	101.11			K	147.11	74.06	1

21)

Name	Sequence	RNA PTMs	M/Z	z	Position of XL
**HNRNPH1**	G(+557.06)LPF(+151.05)GC(+57.02)SK	GG	787.2680	2	Via base to F

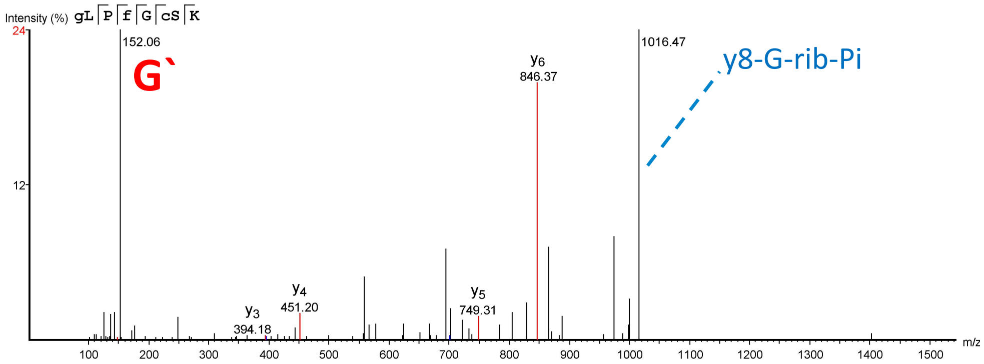

#	Immonium	b	b (2+)	Seq	y	y (2+)	#
1	587.09	615.09	308.04	G(+557.06)			8
2	86.10	728.17	364.58	L	959.45	480.23	7
3	70.07	825.22	413.11	P	846.37	423.68	6
4	271.13	1123.34	562.17	F(+151.05)	749.31	375.16	5
5	30.03	1180.36	590.68	G	451.20	226.10	4
6	133.04	1340.39	670.70	C(+57.02)	394.18	197.59	3
7	60.04	1427.42	714.21	S	234.14	117.57	2
8	101.11			K	147.11	74.06	1

Spectrum legend: For each spectrum the top panel is a table with details of the XL-peptide. The bottom panel is a MS2 spectral annotation view from PEAKS Studio (on left) along with matched ion series table (on right) in which ions detected in the MS2 spectrum are colored in blue (b series) and red (y series), respectively.
