# Supplementary material for: CAPRI enables comparison of evolutionarily conserved RNA interacting regions
Source: Nat Commun. 2019 Jun 18;10:2682. doi: 10.1038/s41467-019-10585-3 (PMC6581911; doi:10.1038/s41467-019-10585-3)

Supplementary Data 6

CAPRI peptides annotated on PDB structures. The crosslinked amino acid is annotated with a stick model. Below each structure example spectra of each of the XL-peptides are shown with annotations.

XL-peptide
ADJ-peptide
RNA
Protein

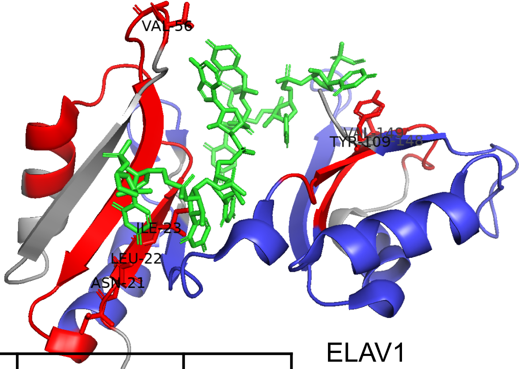

ELAV1
PDB-ID: 4ED5

1)

Name	Sequence	RNA PTMs	M/Z	z
ELAV1	V(+112.03)(+518.03)AGHSLGYGFVNYVTAK	UU	1206.9915	2

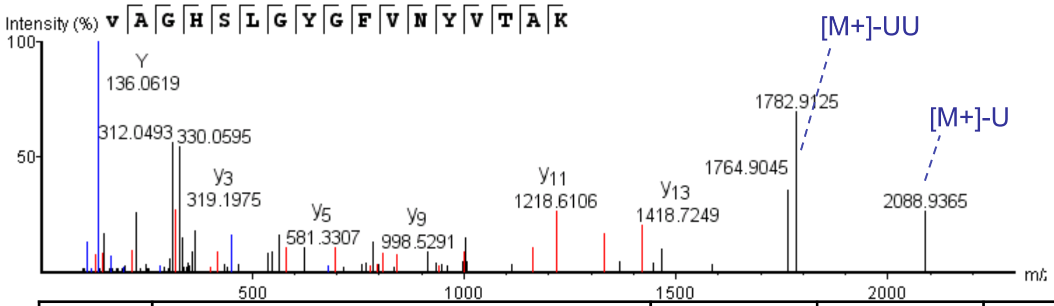

2)

Name	Sequence	RNA PTMs	M/Z	z
ELAV1	T(+112.03)(+212.01)NLIVNYLPQNMTQDELR	U	1243.5675	2

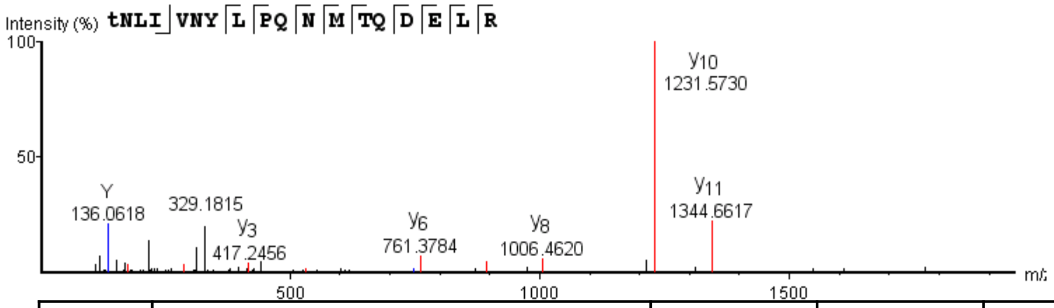

3)

Name	Sequence	RNA PTMs	M/Z	z
ELAV1	D(+212.01)ANLY(+112.03)ISGLPR	U	771.8448	2

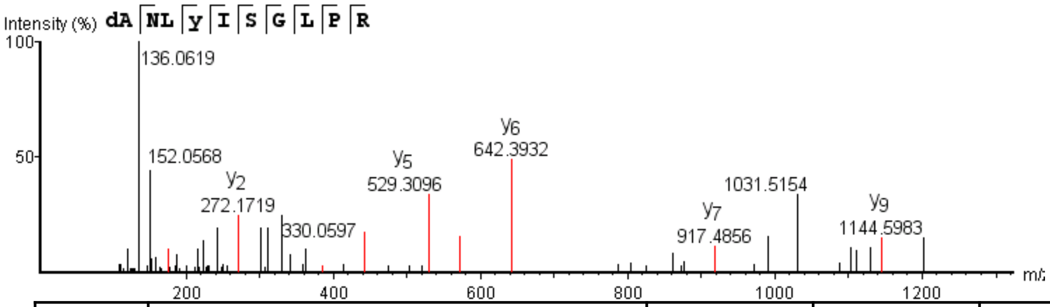

4)

Name	Sequence	RNA PTMs	M/Z	z
ELAV1	G(+212.01)(+112.03)VAFIR	U	493.72095	2

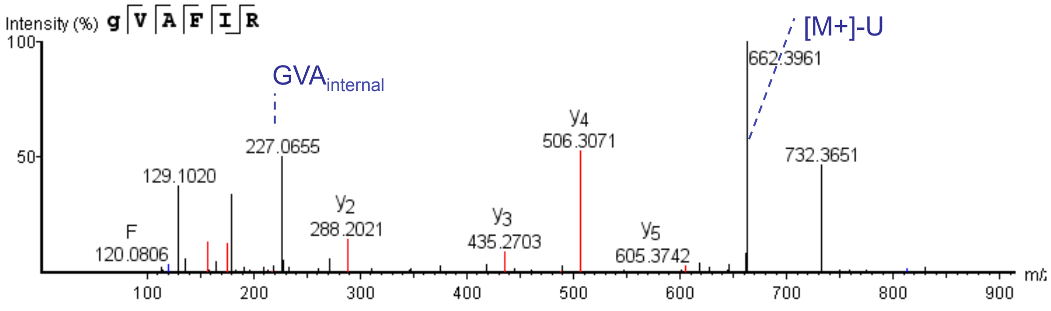

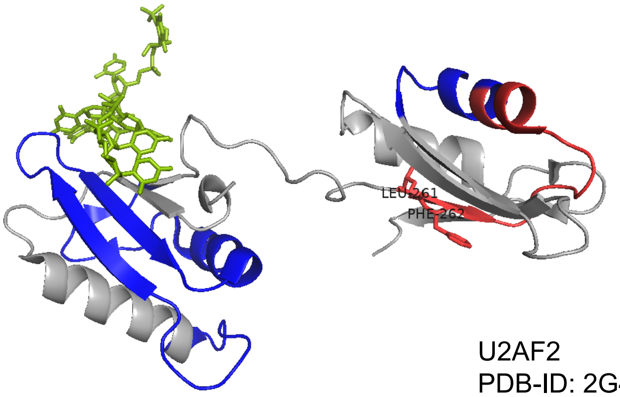

U2AF2
PDB-ID: 2G4B

5)

Name	Sequence	RNA PTMs	M/Z	z
U2AF2	L(+112.03)(+194.00)FIGGLPNYLNDDQVK	U-H2O	1056.4895	2

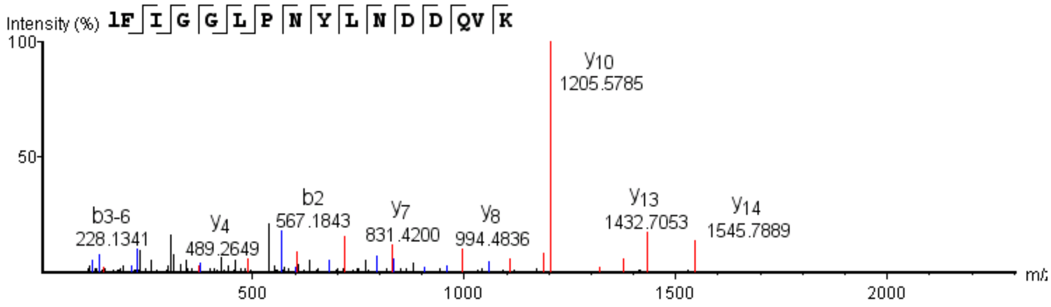

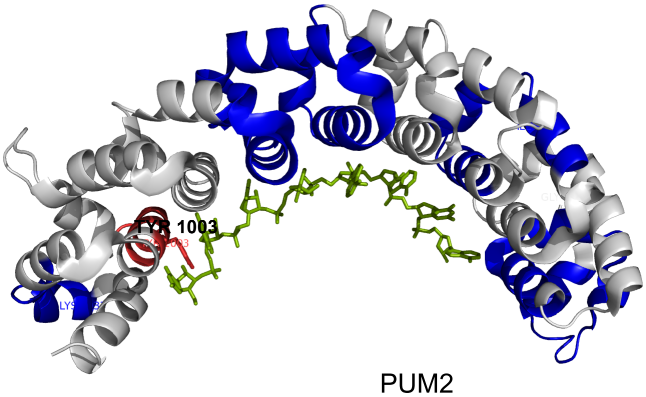

PUM2
PDB-ID: 3Q0Q

6)

Name	Sequence	RNA PTMs	M/Z	z
PUM2	DQYANY(+112.03)VVQK(+212.01)	U	776.32196	2

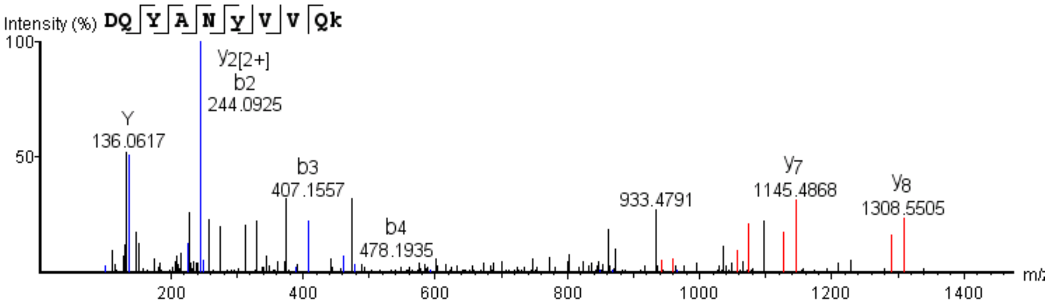

RBMX
PDB-ID: 2MB0

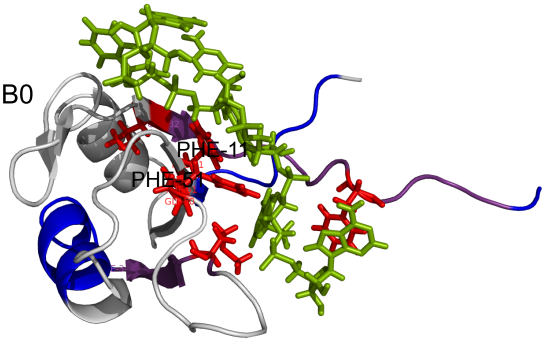

7)

Name	Sequence	RNA PTMs	M/Z	z
RBMX	V(+112.03)(+212.01)EQATKPSFESGR	U	587.25574	3

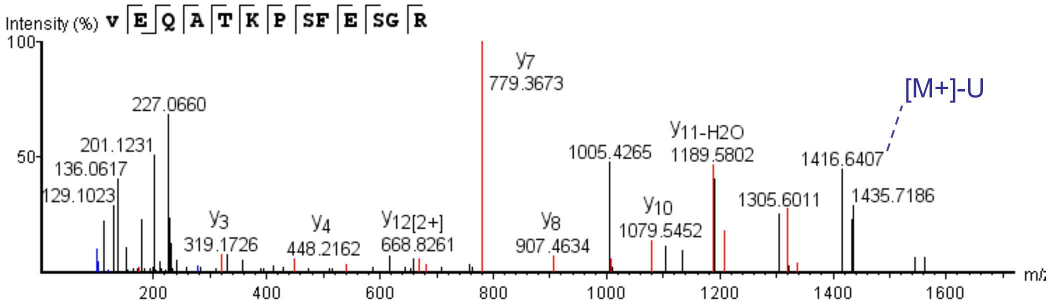

8)

Name	Sequence	RNA PTMs	M/Z	z
RBMX	L(+212.01)F(+112.03)IGGLNTETNEK	U	880.394	2

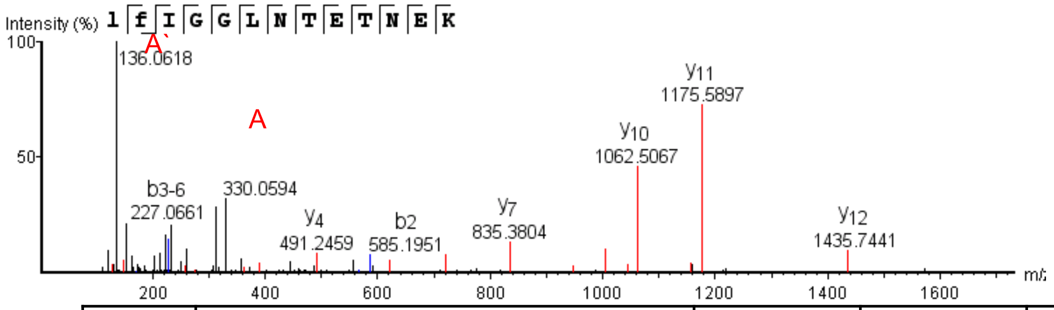

9)

Name	Sequence	RNA PTMs	M/Z	z
RBMX	IVEVLLMK(+112.03)(+194.00)	U-H2O	625.80896	2

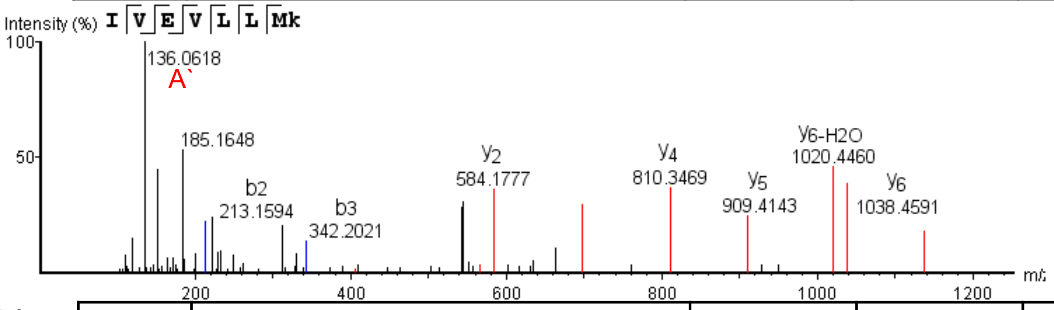

10)

Name	Sequence	RNA PTMs	M/Z	z
RBMX	G(+112.03)(+212.01)FAFVTFESPADAK	U	905.8822	2

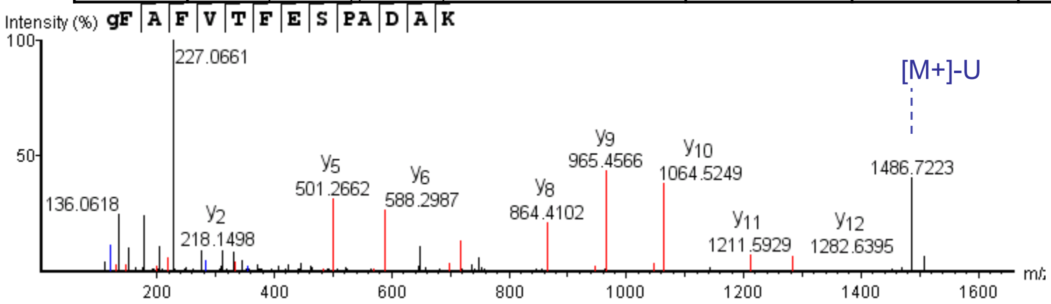

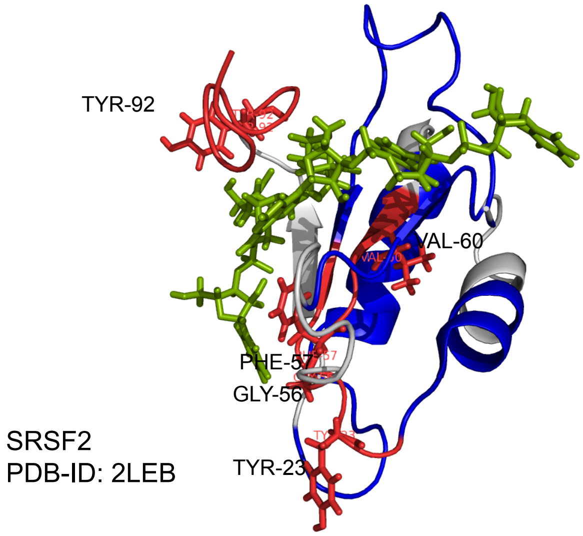

11)

Name	Sequence	RNA PTMs	M/Z	z
SRSF2	Y(+112.03)(+212.01)GRPPDSHHSR	U	544.88885	3

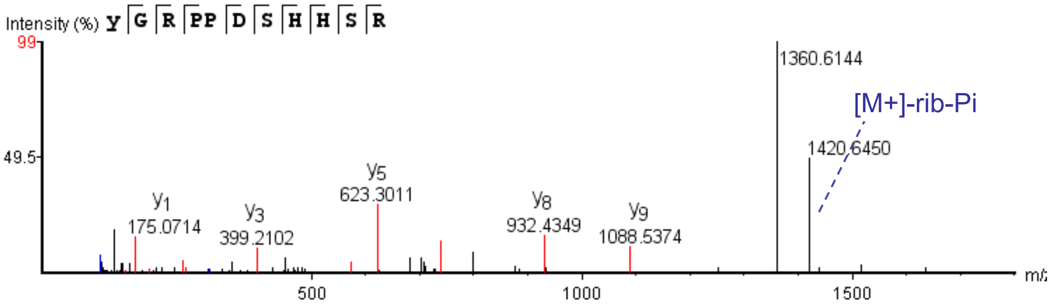

12)

Name	Sequence	RNA PTMs	M/Z	z
SRSF2	VDNLTYR(+112.03)(+194.00) 593.74225	U-H2O	593.74225	2

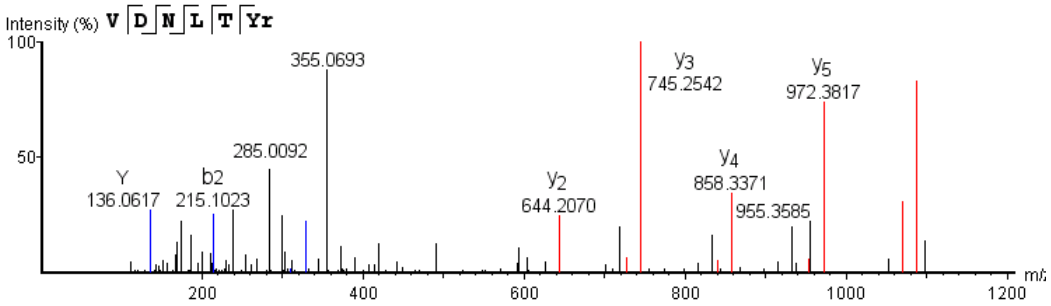

13)

Name	Sequence	RNA PTMs	M/Z	z
SRSF2	G(+112.03)(+212.01)FAFVR 510.71313	U	510.71313	2

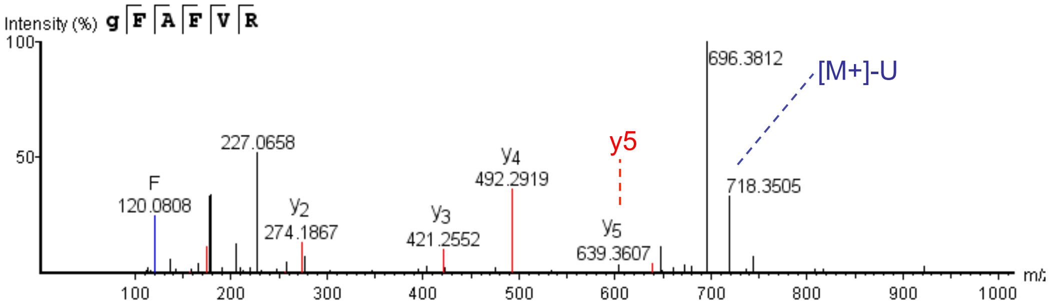

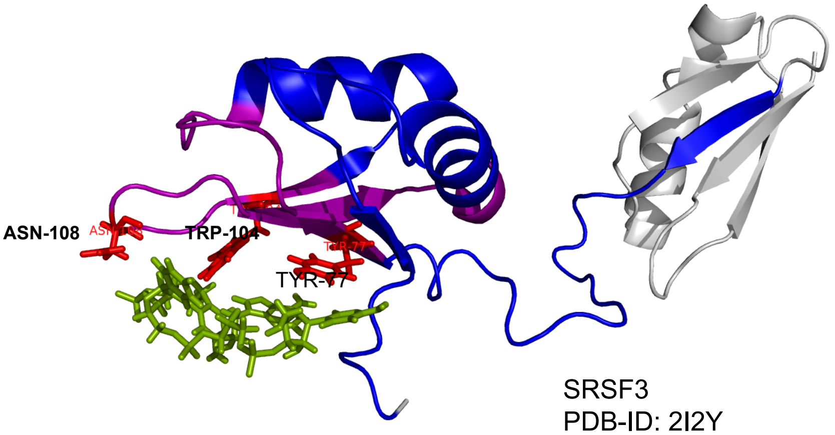

14)

Name	Sequence	RNA PTMs	M/Z	z
SRSF3	V(+112.03)(+212.01)YVGNLGNNGNK	U	786.8383	2

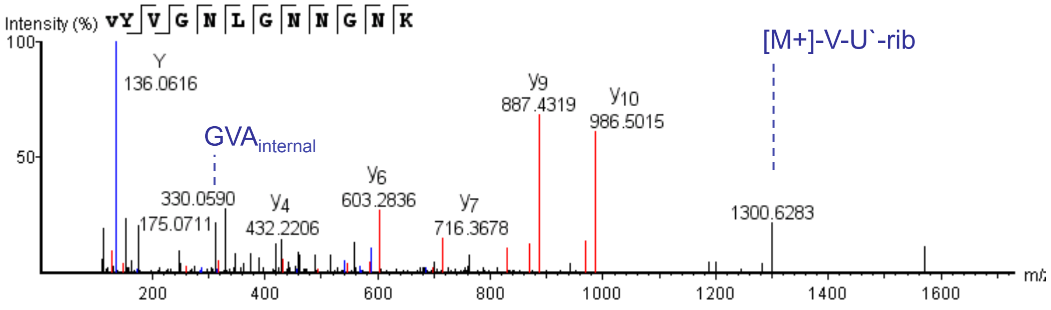

15)

Name	Sequence	RNA PTMs	M/Z	z
SRSF3	S(+518.03)VWVARNP(+135.05)PGFAFVEFEDPR	AU	991.7518	3

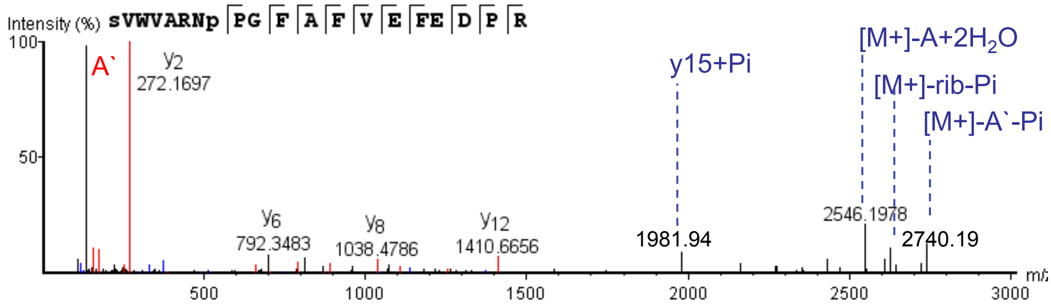

16)

Name	Sequence	RNA PTMs	M/Z	z
SRSF3	S(+517.05)VWVARN(+112.03)PPGFAFVEFEDPR	CU	983.7484	3

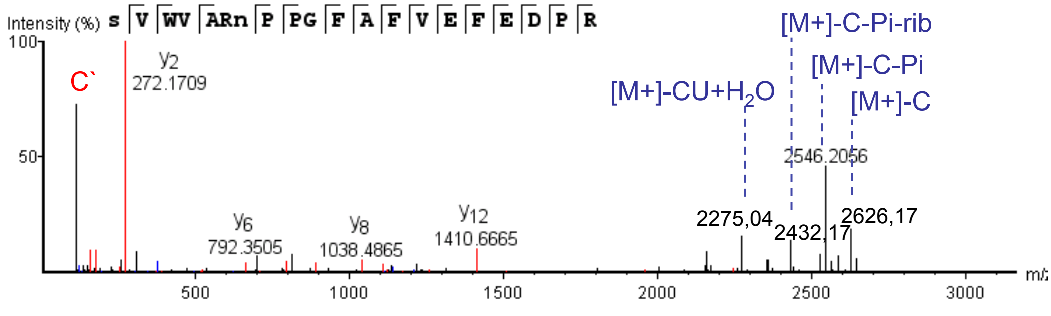

Supplement: Supplementary file 9 — Supplementary Data 6 [file 41467_2019_10585_MOESM9_ESM.pdf]
