# Supplementary material for: CAPRI enables comparison of evolutionarily conserved RNA interacting regions
Source: Nat Commun. 2019 Jun 18;10:2682. doi: 10.1038/s41467-019-10585-3 (PMC6581911; doi:10.1038/s41467-019-10585-3)
Supplement: Supplementary file 10 — Supplementary Data 7 [file 41467_2019_10585_MOESM10_ESM.pdf]

Supplementary Data 7

Drosophila XL-peptides identifying Novel sites of interaction

Top panels: Table with details of the XL-peptide.
Middle panels: ADJ- and XL- peptide coverage maps of proteins with domain annotations.
Bottom panels: MS2 spectral annotation view from PEAKS Studio along with matched ion series table in which ions detected in the MS2 spectrum are colored in blue (b series) and red (y series), respectively.

1)

Uniprot ID	Name	Sequence	RNA PTMs	M/Z	-lgP	z	start	end
O01666	ATPsyngamma	S(+112.03)(+212.01)VVSYQC(+57.02)STLPIFSGSTVEK	U',rest	1257.0665	33.28	2	191	210

ATPsyngamma

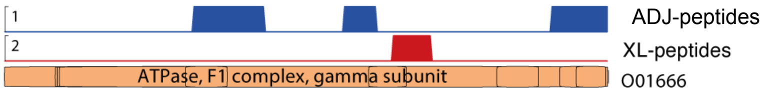

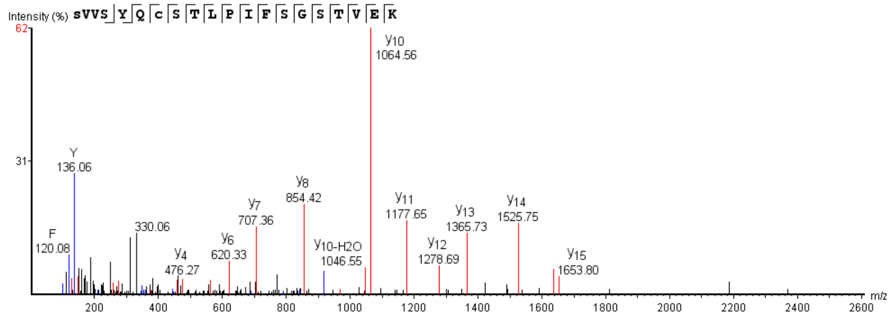

#	Immonium	b	b-H2O	b (2+)	Seq	y	y-H2O	y (2+)	#
1	384.08	412.08	394.07	206.54	S(+324.04)				20
2	72.08	511.14	493.13	256.07	V	2102.05	2084.04	1051.52	19
3	72.08	610.21	592.20	305.61	V	2002.98	1984.97	1001.99	18
4	60.04	697.24	679.23	349.11	S	1903.91	1885.90	952.46	17
5	136.06	860.31	842.29	430.65	Y	1816.88	1798.87	908.94	16
6	101.07	988.37	970.36	494.68	Q	1653.80	1635.80	827.41	15
7	133.04	1148.40	1130.39	574.70	C(+57.02)	1525.75	1507.75	763.38	14
8	60.04	1235.43	1217.42	618.21	S	1365.73	1347.72	683.36	13
9	74.06	1336.48	1318.47	668.74	T	1278.69	1260.68	639.85	12
10	86.10	1449.56	1431.55	725.28	L	1177.65	1159.64	589.32	11
11	70.07	1546.61	1528.60	773.81	P	1064.56	1046.55	532.78	10
12	86.10	1659.70	1641.69	830.35	I	967.51	949.50	484.25	9
13	120.08	1806.77	1788.76	903.88	F	854.42	836.41	427.71	8
14	60.04	1893.80	1875.79	947.40	S	707.36	689.35	354.18	7
15	30.03	1950.82	1932.81	975.91	G	620.33	602.31	310.66	6
16	60.04	2037.85	2019.84	1019.43	S	563.30	545.29	282.15	5
17	74.06	2138.90	2120.89	1069.95	T	476.27	458.26	238.64	4
18	72.08	2237.97	2219.96	1119.48	V	375.22	357.21	188.11	3
19	102.06	2367.01	2349.00	1184.01	E	276.16	258.14	138.58	2
20	101.11				K	147.11	129.10	74.06	1

2)

Name	Uniprot ID	Sequence	RNA PTMs	M/Z	-lgP	z	start	end
bic	Q7KM15	I(+112.03)(+212.01)VHSTPATDDK	U',rest	754.31885	23.05	2	27	37

bic

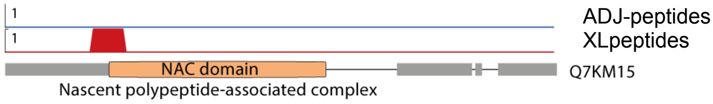

bic_Q7KM15_I(+112.03)(+212.01)VHSTPATDDK_754.31885_2

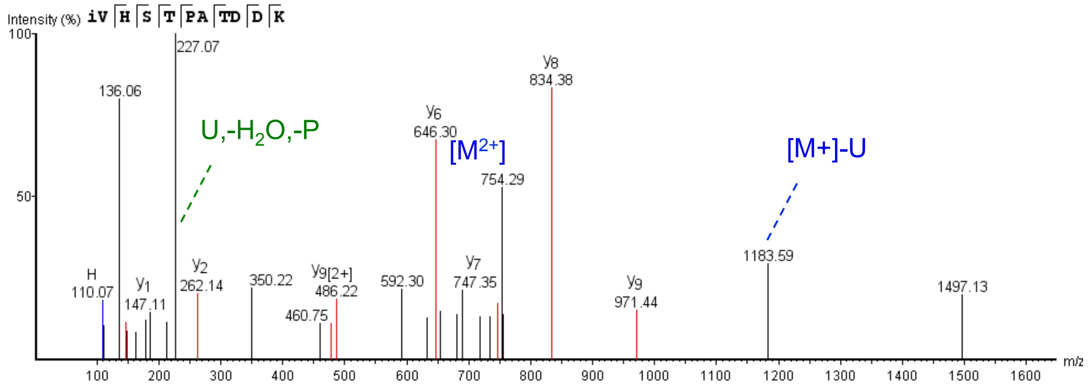

#	Immonium	b	b-H2O	b (2+)	Seq	y	y-H2O	y (2+)	#
1	410.13	438.13	420.12	219.56	I(+324.04)				11
2	72.08	537.20	519.19	269.10	V	1070.51	1052.50	535.76	10
3	110.07	674.26	656.24	337.63	H	971.44	953.43	486.22	9
4	60.04	761.29	743.28	381.14	S	834.38	816.37	417.69	8
5	74.06	862.33	844.32	431.67	T	747.35	729.34	374.18	7
6	70.07	959.39	941.38	480.19	P	646.30	628.29	323.65	6
7	44.05	1030.42	1012.41	515.71	A	549.25	531.24	275.13	5
8	74.06	1131.47	1113.46	566.24	T	478.21	460.20	239.61	4
9	88.04	1246.50	1228.49	623.75	D	377.17	359.16	189.08	3
10	88.04	1361.53	1343.52	681.26	D	262.14	244.13	131.57	2
11	101.11				K	147.11	129.10	74.06	1

3)

Name	Uniprot ID	Sequence	RNA PTMs	M/Z	-lgP	z	start	end
bsf	Q9VJ86	R.M(+212.01)Q(+112.03)QEFSVFPNSETVR.D	U'; rest	1061.9438	29.92	2	441	455

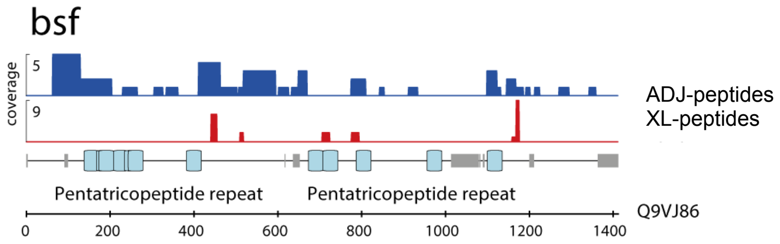

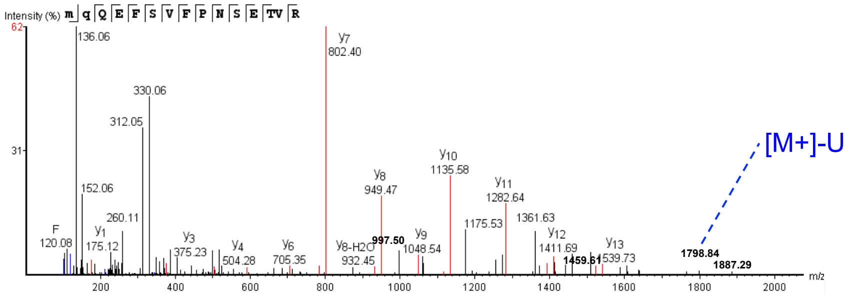

#	Immonium	b	b-H2O	b (2+)	Seq	y	y-H2O	y (2+)	#
1	316.06	344.07	326.05	172.53	M(+212.01)				15
2	213.10	584.14	566.13	292.57	Q(+112.03)	1779.83	1761.82	890.41	14
3	101.07	712.20	694.19	356.60	Q	1539.73	1521.74	770.37	13
4	102.06	841.24	823.23	421.12	E	1411.69	1393.66	706.34	12
5	120.08	988.31	970.30	494.66	F	1282.64	1264.63	641.82	11
6	60.04	1075.34	1057.33	538.17	S	1135.58	1117.57	568.29	10
7	72.08	1174.41	1156.40	587.71	V	1048.54	1030.53	524.77	9
8	120.08	1321.48	1303.47	661.24	F	949.47	931.45	475.24	8
9	70.07	1418.53	1400.52	709.77	P	802.40	784.40	401.70	7
10	87.06	1532.58	1514.57	766.79	N	705.35	687.34	353.18	6
11	60.04	1619.61	1601.60	810.30	S	591.31	573.30	296.15	5
12	102.06	1748.65	1730.64	874.83	E	504.28	486.27	252.64	4
13	74.06	1849.70	1831.69	925.35	T	375.23	357.22	188.12	3
14	72.08	1948.77	1930.76	974.88	V	274.19	256.18	137.59	2
15	129.11				R	175.12	157.11	88.06	1

4)

Name	Uniprot ID	Sequence	RNA PTMs	M/Z	-lgP	z	start	end
bsf	Q9VJ86	RL(+112.03)ISFDNR	U'	566.79675	22.36	2	1169	1176

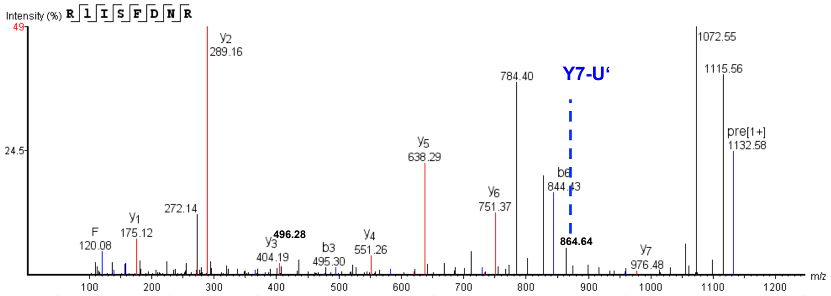

#	Immonium	b	b-H2O	b (2+)	Seq	y	y-H2O	y (2+)	#
1	129.11	157.11	139.09	79.05	R				8
2	198.12	382.22	364.21	191.61	L(+112.03)	976.48	958.48	488.74	7
3	86.10	495.30	477.28	248.15	I	751.37	733.36	376.19	6
4	60.04	582.34	564.33	291.67	S	638.29	620.28	319.64	5
5	120.08	729.40	711.39	365.19	F	551.26	533.25	276.13	4
6	88.04	844.43	826.42	422.72	D	404.19	386.18	202.59	3
7	87.06	958.48	940.46	479.74	N	289.16	271.15	145.08	2
8	129.11				R	175.12	157.11	88.06	1

5)

Name	Uniprot ID	Sequence	RNA PTMs	M/Z	-lgP	z	start	end
bsf	Q9VJ86	K.R(+112.03)(+194.00)LISFDNR.F	U'; cyclicrest	663.7967	26.96	2	1169	1176

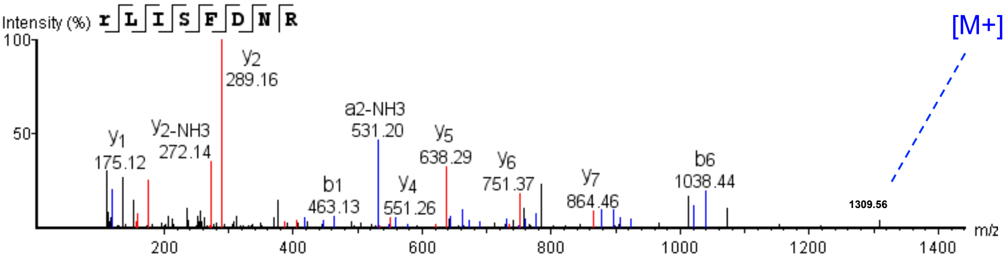

#	Immonium	b	b-NH3	a	a-NH3	Seq	x	y	y-H2O	y-NH3	#
1	435.14	463.13	446.11	435.14	418.11	R(+306.03)					8
2	86.10	576.22	559.19	548.22	531.20	L	890.44	864.46	846.45	847.43	7
3	86.10	689.30	672.28	661.31	644.28	I	777.35	751.37	733.36	734.35	6
4	60.04	776.34	759.31	748.34	731.31	S	664.27	638.29	620.28	621.26	5
5	120.08	923.41	906.39	895.41	878.38	F	577.24	551.26	533.25	534.23	4
6	88.04	1038.44	1021.40	1010.43	993.41	D	430.17	404.19	386.18	387.16	3
7	87.06	1152.47	1135.45	1124.48	1107.45	N	315.14	289.16	271.15	272.14	2
8	129.11					R	201.10	175.12	157.11	158.09	1

6)

Name	Uniprot ID	Sequence	RNA PTMs	M/Z	-lgP	z	start	end
CG10565	Q9VP77	R.V(+42.01)(+112.03)KELVELVNSK.K	U'	706.3903	13.18	2	628	638

CG10565

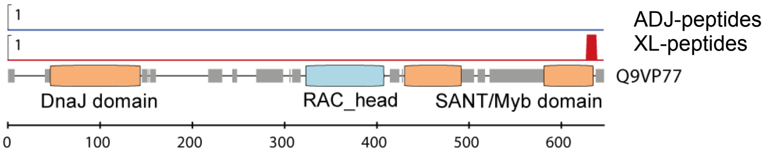

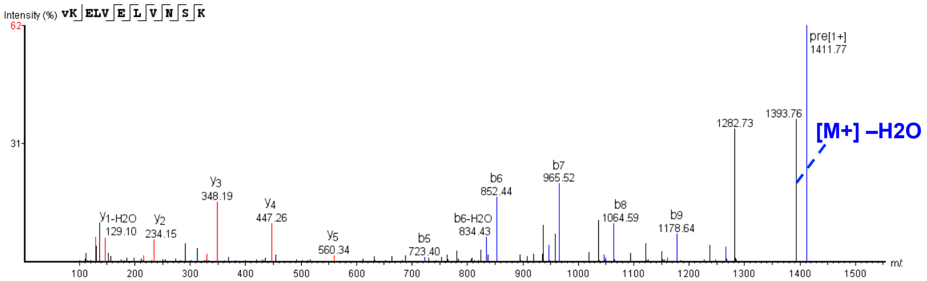

#	Immonium	b	b-H2O	b (2+)	Seq	y	y-H2O	y (2+)	#
1	226.12	254.11	236.10	127.56	V(+154.04)				11
2	101.11	382.20	364.20	191.60	K	1158.67	1140.66	579.84	10
3	102.06	511.25	493.24	256.13	E	1030.58	1012.57	515.79	9
4	86.10	624.34	606.33	312.67	L	901.54	883.52	451.27	8
5	72.08	723.40	705.39	362.20	V	788.45	770.44	394.73	7
6	102.06	852.44	834.43	426.72	E	689.38	671.37	345.19	6
7	86.10	965.52	947.51	483.27	L	560.34	542.33	280.67	5
8	72.08	1064.59	1046.59	532.80	V	447.26	429.25	224.13	4
9	87.06	1178.64	1160.63	589.82	N	348.19	330.18	174.59	3
10	60.04	1265.67	1247.66	633.34	S	234.15	216.13	117.57	2
11	101.11				K	147.11	129.10	74.06	1

7)

Name	Uniprot ID	Sequence	RNA PTMs	M/Z	-lgP	z	start	end
CG15784	Q9W4C1	R.L(+111.04)(+541.06)GHGFGHK.H	A+rest; C’	752.78	13.79	2	186	193

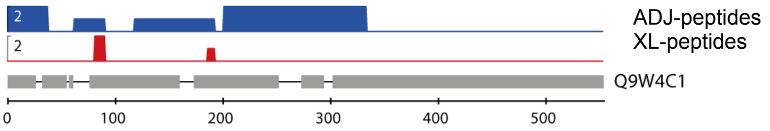

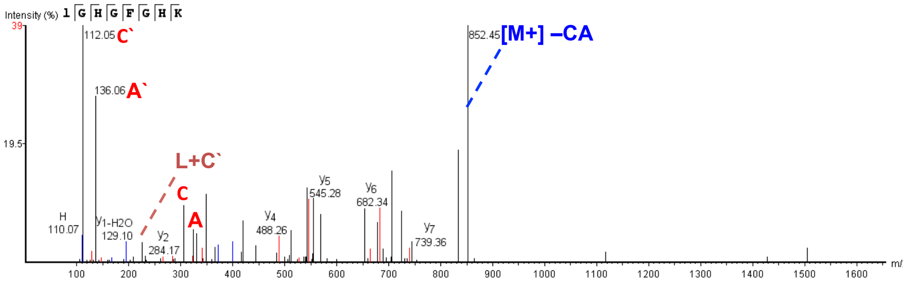

#	Immonium	b	b-H2O	b (2+)	Seq	y	y-H2O	y (2+)	#
1	738.20	766.20	748.19	383.60	L(+652.10)				8
2	30.03	823.22	805.21	412.11	G	739.36	721.35	370.18	7
3	110.07	960.28	942.27	480.64	H	682.34	664.33	341.67	6
4	30.03	1017.30	999.29	509.15	G	545.28	527.28	273.14	5
5	120.08	1164.37	1146.36	582.68	F	488.26	470.25	244.63	4
6	30.03	1221.39	1203.38	611.19	G	341.19	323.18	171.10	3
7	110.07	1358.45	1340.44	679.72	H	284.17	266.16	142.59	2
8	101.11				K	147.11	129.10	74.06	1

8)

Name	Uniprot ID	Sequence	RNA PTMs	M/Z	-lgP	z	start	end
CG15784	Q9W4C1	R.C(+112.03)RQQEQEQGTK.R	U’	723.8231	48.57	2	81	91

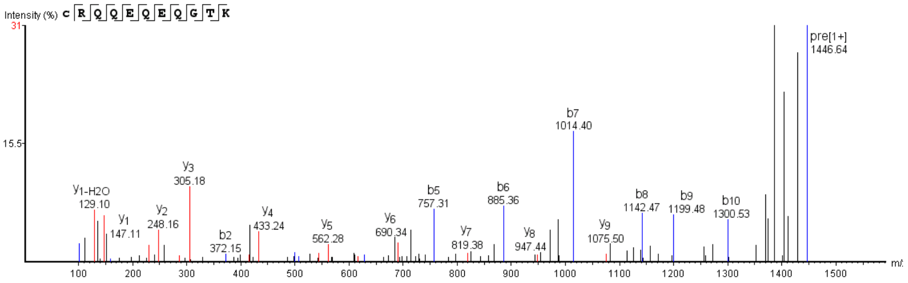

#	Immonium	b	b-H2O	b (2+)	Seq	y	y-H2O	y (2+)	#
1	188.05	216.04	198.03	108.52	C(+112.03)				11
2	129.10	372.15	354.13	186.57	R	1231.60	1213.59	616.31	10
3	101.07	500.20	482.19	250.60	Q	1075.50	1057.49	538.25	9
4	101.07	628.26	610.25	314.63	Q	947.44	929.43	474.22	8
5	102.06	757.31	739.29	379.15	E	819.38	801.37	410.19	7
6	101.07	885.36	867.35	443.18	Q	690.34	672.33	345.67	6
7	102.06	1014.40	996.40	507.70	E	562.28	544.27	281.64	5
8	101.07	1142.47	1124.45	571.73	Q	433.24	415.23	217.12	4
9	30.03	1199.48	1181.48	600.24	G	305.18	287.17	153.09	3
10	74.06	1300.53	1282.52	650.77	T	248.16	230.15	124.58	2
11	101.11				K	147.11	129.10	74.06	1

9)

Name	Uniprot ID	Sequence	RNA PTMs	M/Z	-lgP	z	start	end
CG2807 / sf3b1	Q9VPR5	MEN(+.98)IP(+151.05)R.T	G’	456.2123	15.07	2	1	6

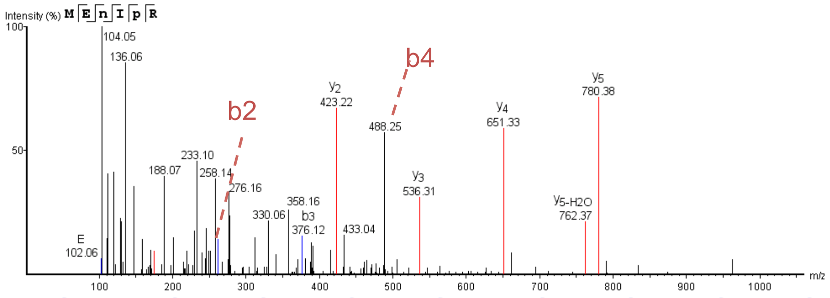

#	Immonium	b	b-H2O	b (2+)	Seq	y	y-H2O	y (2+)	#
1	104.05	132.05	114.04	66.52	M				6
2	102.06	261.09	243.08	131.05	E	780.38	762.37	390.69	5
3	88.04	376.12	358.11	188.56	N(+.98)	651.33	633.32	326.17	4
4	86.10	489.20	471.19	245.10	I	536.31	518.29	268.65	3
5	221.11	737.30	719.29	369.15	P(+151.05)	423.22	405.21	212.11	2
6	129.11				R	175.12	157.11	88.06	1

10)

Name	Uniprot ID	Sequence	RNA PTMs	M/Z	-lgP	z	start	end
CG42668	Q7KSB3	MNLL(+112.03)QR.I	U’	443.7318	15.03	2	0	5

CG42668

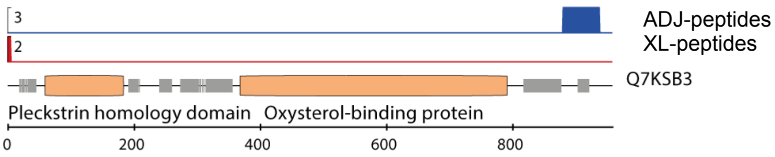

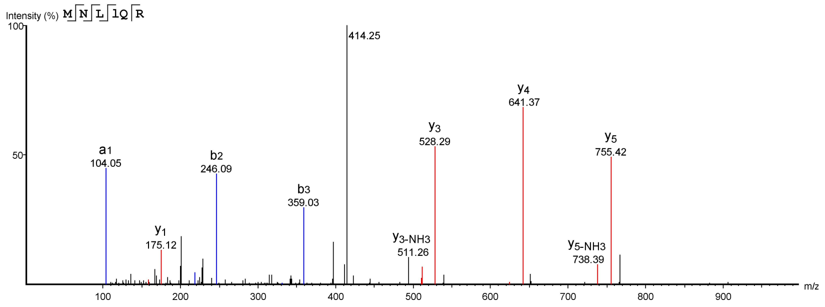

#	Immonium	b	b-H2O	b (2+)	Seq	y	y-H2O	y (2+)	#
1	104.05	132.05	114.04	66.52	M				6
2	87.06	246.09	228.08	123.55	N	755.42	737.41	378.21	5
3	86.10	359.17	341.17	180.09	L	641.37	623.36	321.19	4
4	198.12	584.29	566.28	292.64	L(+112.03)	528.29	510.28	264.64	3
5	101.07	712.35	694.33	356.67	Q	303.18	285.17	152.09	2
6	129.11				R	175.12	157.11	88.06	1

11)

Name	Uniprot ID	Sequence	RNA PTMs	M/Z	-lgP	z	start	end
CG7518	Q9VG05	R.C(+112.03)(+212.01)EHLQTQQAPR.S	U; rest	817.8358	36.2	2	1660	1670

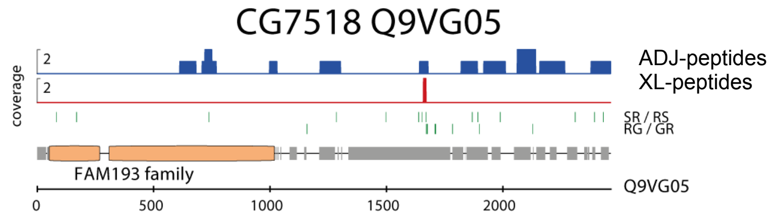

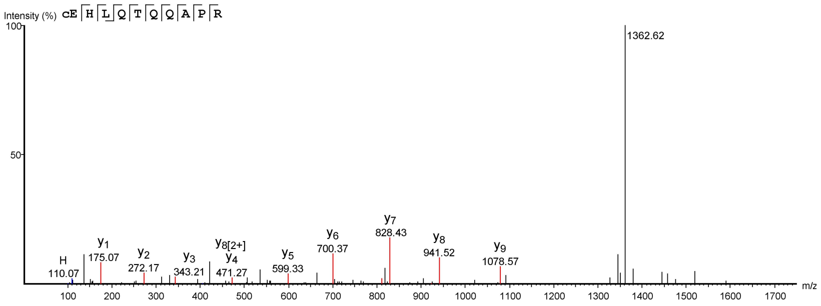

#	Immonium	b	b-H2O	b (2+)	Seq	y	y-H2O	y (2+)	#
1	400.06	428.05	410.04	214.53	C(+324.04)				11
2	102.06	557.10	539.08	279.05	E	1207.62	1189.61	604.31	10
3	110.07	694.15	676.14	347.58	H	1078.57	1060.56	539.79	9
4	86.10	807.24	789.23	404.10	L	941.52	923.51	471.27	8
5	101.07	935.30	917.29	468.15	Q	828.43	810.42	414.72	7
6	74.06	1036.34	1018.33	518.67	T	700.37	682.36	350.69	6
7	101.07	1164.40	1146.39	582.70	Q	599.33	581.32	300.16	5
8	101.07	1292.46	1274.45	646.73	Q	471.27	453.26	236.13	4
9	44.05	1363.50	1345.49	682.25	A	343.21	325.20	172.10	3
10	70.07	1460.55	1442.54	730.78	P	272.17	254.16	136.59	2
11	129.11				R	175.12	157.11	88.06	1

12)

Name	Uniprot ID	Sequence	RNA PTMs	M/Z	-lgP	z	start	end
Hrb87F	E1JIK0	R.N(+112.03)(+212.01)SNFGNNRPAPYSQGGGGGGFNK.G	U; rest	874.3647	27.58	3	331	353

Hrb87F

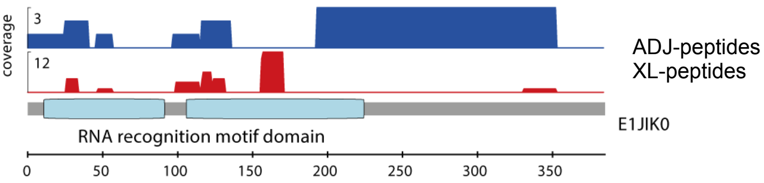

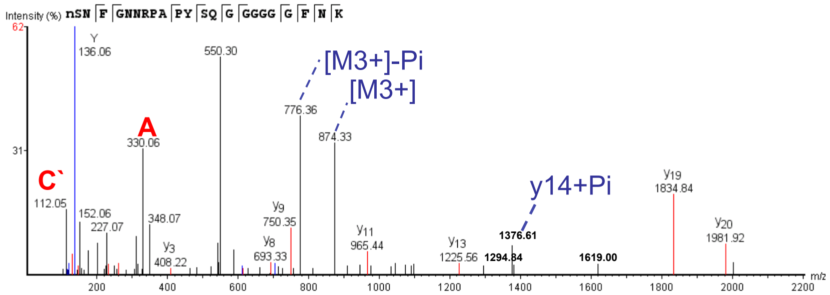

#	Immonium	b	b-H2O	b (2+)	Seq	y	y-H2O	y (2+)	#
1	411.09	439.09	421.08	220.04	N(+324.04)				23
2	60.04	526.12	508.11	263.56	S	2183.00	2164.99	1092.00	22
3	87.06	640.16	622.15	320.58	N	2095.97	2077.96	1048.48	21
4	120.08	787.23	769.22	394.11	F	1981.92	1963.92	991.46	20
5	30.03	844.25	826.24	422.63	G	1834.84	1816.85	917.93	19
6	87.06	958.29	940.28	479.65	N	1777.84	1759.83	889.42	18
7	87.06	1072.34	1054.33	536.67	N	1663.79	1645.78	832.40	17
8	129.10	1228.44	1210.43	614.72	R	1549.75	1531.74	775.38	16
9	70.07	1325.49	1307.48	663.25	P	1393.65	1375.64	697.32	15
10	44.05	1396.53	1378.52	698.76	A	1296.60	1278.59	648.80	14
11	70.07	1493.58	1475.57	747.29	P	1225.56	1207.55	613.28	13
12	136.06	1656.64	1638.63	828.82	Y	1128.51	1110.50	564.75	12
13	60.04	1743.68	1725.67	872.34	S	965.44	947.43	483.22	11
14	101.07	1871.73	1853.72	936.37	Q	878.41	860.40	439.71	10
15	30.03	1928.76	1910.75	964.88	G	750.35	732.34	375.68	9
16	30.03	1985.78	1967.77	993.39	G	693.33	675.32	347.17	8
17	30.03	2042.80	2024.79	1021.90	G	636.31	618.30	318.65	7
18	30.03	2099.82	2081.81	1050.41	G	579.29	561.28	290.14	6
19	30.03	2156.84	2138.83	1078.92	G	522.27	504.26	261.63	5
20	30.03	2213.86	2195.85	1107.43	G	465.25	447.24	233.13	4
21	120.08	2360.93	2342.92	1180.97	F	408.22	390.21	204.61	3
22	87.06	2474.98	2456.96	1237.99	N	261.15	243.15	131.08	2
23	101.11				K	147.11	129.10	74.06	1

13)

Name	Uniprot ID	Sequence	RNA PTMs	M/Z	-lgP	z	start	end
Hrb98DE	A4V3J6	R.M(+212.01)Q(+112.03)PYQGGGGFK.A	U; rest	747.2928	20.55	2	326	336

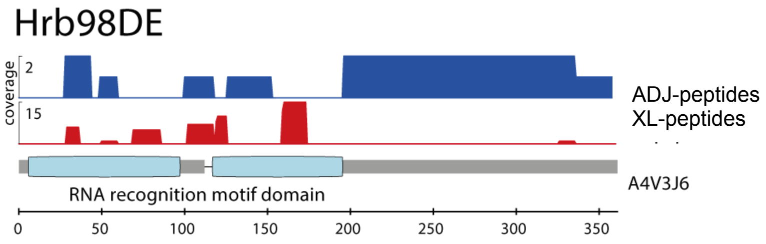

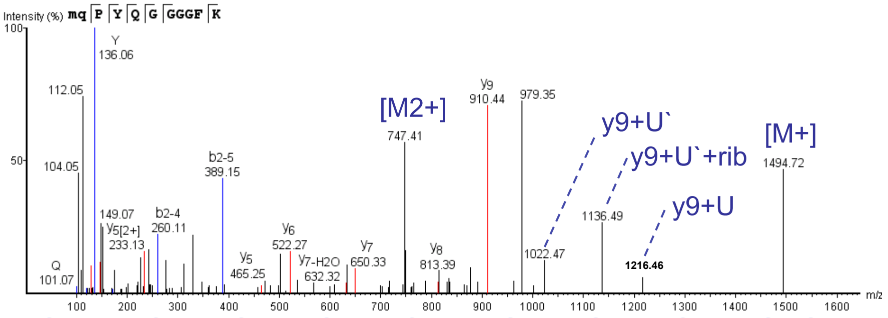

#	Immonium	b	b-H2O	b (2+)	Seq	y	y-H2O	y (2+)	#
1	316.06	344.06	326.05	172.53	M(+212.01)				11
2	213.10	584.14	566.13	292.57	Q(+112.03)	1150.53	1132.52	575.76	10
3	70.07	681.20	663.18	341.10	P	910.44	892.43	455.72	9
4	136.06	844.26	826.25	422.63	Y	813.39	795.38	407.19	8
5	101.07	972.32	954.31	486.66	Q	650.33	632.32	325.66	7
6	30.03	1029.34	1011.33	515.17	G	522.27	504.26	261.63	6
7	30.03	1086.36	1068.35	543.68	G	465.25	447.24	233.13	5
8	30.03	1143.38	1125.37	572.19	G	408.22	390.21	204.61	4
9	30.03	1200.40	1182.39	600.70	G	351.20	333.19	176.10	3
10	120.08	1347.47	1329.46	674.24	F	294.18	276.17	147.59	2
11	101.11				K	147.11	129.10	74.06	1

14)

Name	Uniprot ID	Sequence	RNA PTMs	M/Z	-lgP	z	start	end
l(2)35Bd	Q9VJQ4	R.M(+112.03)(+212.01)SGLESVQPQR.C	U; rest	778.3262	16.11	2	357	367

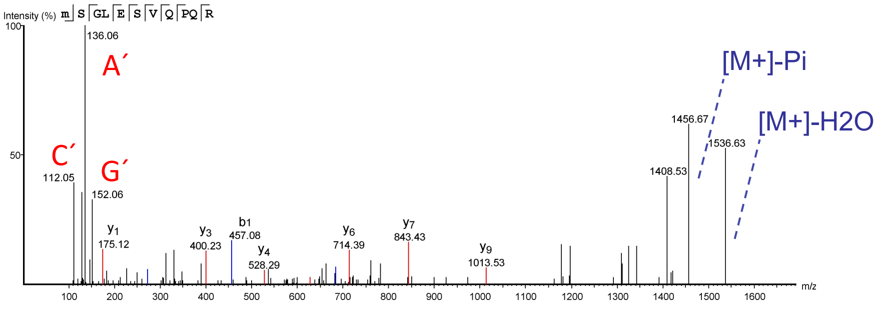

#	Immonium	b	b-H2O	b (2+)	Seq	y	y-H2O	y (2+)	#
1	428.09	456.08	438.07	228.54	M(+324.04)				11
2	60.04	543.12	525.11	272.06	S	1100.57	1082.56	550.78	10
3	30.03	600.14	582.13	300.57	G	1013.53	995.53	507.27	9
4	86.10	713.22	695.21	357.11	L	956.52	938.51	478.76	8
5	102.06	842.26	824.25	421.63	E	843.43	825.42	422.22	7
6	60.04	929.30	911.29	465.15	S	714.39	696.38	357.69	6
7	72.08	1028.36	1010.35	514.68	V	627.35	609.35	314.18	5
8	101.07	1156.42	1138.41	578.71	Q	528.29	510.28	264.64	4
9	70.07	1253.48	1235.47	627.24	P	400.23	382.22	200.62	3
10	101.07	1381.53	1363.52	691.27	Q	303.18	285.17	152.09	2
11	129.11				R	175.12	157.11	88.06	1

15)

Name	Uniprot ID	Sequence	RNA PTMs	M/Z	-lgP	z	start	end
lost	Q9VN21	R.V(+112.03)(+212.01)LHEFK.E	U; rest	548.7398	14.49	2	93	98

5-FTHF cyclo-ligase

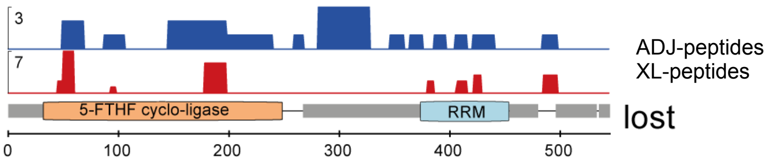

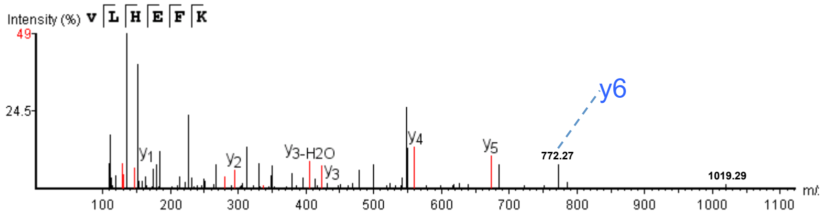

#	b	b-H2O	b-NH3	b (2+)	Seq	y	y-H2O	y-NH3	y (2+)	#
1	424.11	406.10	407.09	212.56	V(+324.04)					6
2	537.20	519.19	520.17	269.10	L	673.37	655.36	656.34	337.19	5
3	674.26	656.24	657.23	337.63	H	560.28	542.27	543.26	280.64	4
4	803.30	785.29	786.27	402.15	E	423.22	405.21	406.20	212.11	3
5	950.37	932.36	933.34	475.68	F	294.18	276.17	277.15	147.59	2
6					K	147.11	129.10	130.09	74.06	1

16)

Name	Uniprot ID	Sequence	RNA PTMs	M/Z	-lgP	z	start	end
lost	Q9VN21	R.G(+112.03)(+212.01)NGFADLDIGLLIELGAITPK.T	U; rest	1226.1115	47.26	2	178	198

67) Q9VN21_G(+112.03)(+212.01)NGFADLDIGLLIELGAITPK_1226.1115_2_178_198

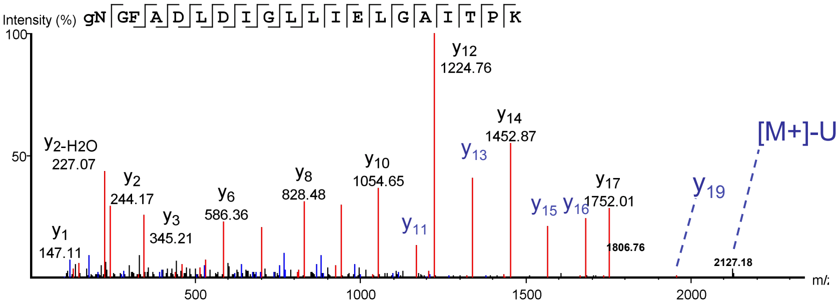

#	Immonium	b	b-H2O	b-NH3	b (2+)	Seq	y	y-H2O	y-NH3	y (2+)	#
1	354.07	382.07	364.05	365.04	191.53	G(+324.04)					21
2	87.06	496.11	478.10	479.08	248.55	N	2070.15	2052.14	2053.12	1035.57	20
3	30.03	553.13	535.12	536.10	277.06	G	1956.12	1938.09	1939.08	978.55	19
4	120.08	700.20	682.18	683.17	350.60	F	1899.08	1881.07	1882.06	950.04	18
5	44.05	771.25	753.24	754.21	386.12	A	1752.01	1734.00	1735.00	876.51	17
6	88.04	886.26	868.25	869.24	443.63	D	1680.98	1662.97	1663.96	840.99	16
7	86.10	999.36	981.34	982.33	500.17	L	1565.95	1547.94	1548.92	783.48	15
8	88.04	1114.36	1096.35	1097.35	557.69	D	1452.87	1434.84	1435.85	726.93	14
9	86.10	1227.46	1209.44	1210.43	614.23	I	1337.84	1319.83	1320.81	669.42	13
10	30.03	1284.48	1266.47	1267.46	642.74	G	1224.76	1206.74	1207.73	612.88	12
11	86.10	1397.56	1379.55	1380.55	699.28	L	1167.73	1149.72	1150.71	584.37	11
12	86.10	1510.65	1492.64	1493.62	755.82	L	1054.65	1036.64	1037.62	527.83	10
13	86.10	1623.73	1605.72	1606.70	812.37	I	941.56	923.56	924.54	471.28	9
14	102.06	1752.77	1734.76	1735.75	876.89	E	828.48	810.47	811.45	414.74	8
15	86.10	1865.86	1847.85	1848.83	933.43	L	699.44	681.43	682.41	350.22	7
16	30.03	1922.88	1904.87	1905.85	961.94	G	586.36	568.35	569.33	293.68	6
17	44.05	1993.92	1975.91	1976.89	997.46	A	529.33	511.32	512.31	265.17	5
18	86.10	2107.00	2088.99	2089.97	1054.00	I	458.30	440.29	441.27	229.65	4
19	74.06	2208.05	2190.04	2191.02	1104.52	T	345.21	327.20	328.19	173.11	3
20	70.07	2305.10	2287.09	2288.07	1153.05	P	244.17	226.15	227.14	122.58	2
21	101.11					K	147.11	129.10	130.09	74.06	1

17)

Name	Uniprot ID	Sequence	RNA PTMs	M/Z	-lgP	z	start	end
mRpL1	Q9VHT5	K.K(+112.03)(+518.03)VGFIPHNQR.N	U; U+rest	913.3729	19.11	2	52	61

mRpL1

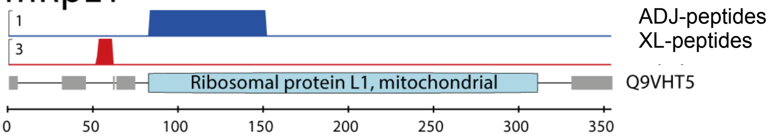

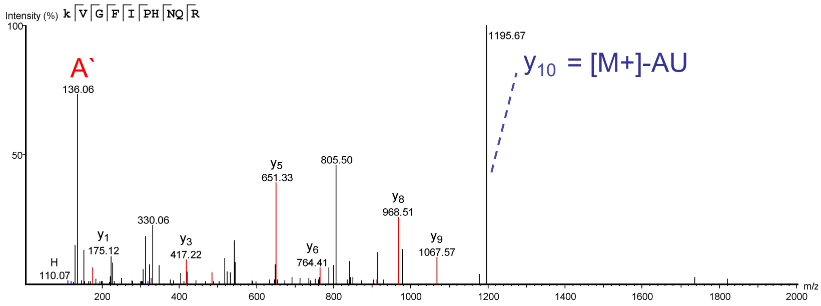

#	Immonium	b	b-H2O	b (2+)	Seq	y	y-H2O	y (2+)	#
1	731.17	759.16	741.15	380.08	K(+630.06)				10
2	72.08	858.23	840.22	429.62	V	1067.57	1049.56	534.29	9
3	30.03	915.25	897.24	458.13	G	968.51	950.50	484.76	8
4	120.08	1062.32	1044.31	531.66	F	911.49	893.47	456.24	7
5	86.10	1175.41	1157.40	588.20	I	764.41	746.41	382.71	6
6	70.07	1272.46	1254.45	636.73	P	651.33	633.32	326.17	5
7	110.07	1409.52	1391.51	705.26	H	554.28	536.27	277.64	4
8	87.06	1523.56	1505.55	762.28	N	417.22	399.21	209.11	3
9	101.07	1651.62	1633.61	826.31	Q	303.18	285.17	152.09	2
10	129.11				R	175.12	157.11	88.06	1

18)

Name	Uniprot ID	Sequence	RNA PTMs	M/Z	-lgP	z	start	end
mRpS30	Q9VXP3	K.Y(+541.06)L(+112.03)ASNRPR.H	A+rest; U	815.3146	20.54	2	480	487

mRpS30

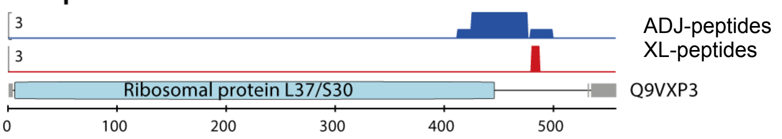

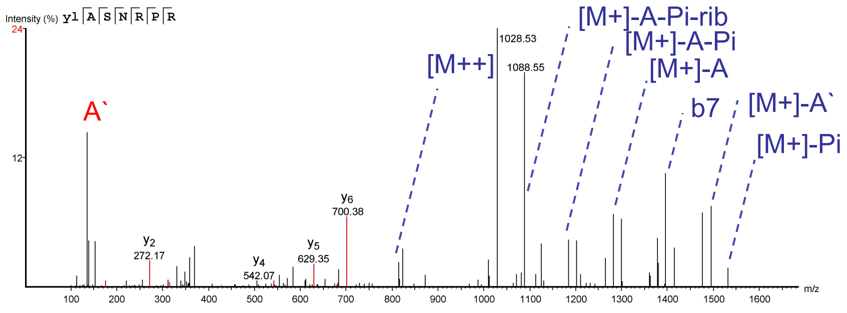

#	Immonium	b	b-H2O	b (2+)	Seq	y	y-H2O	y (2+)	#
1	677.14	705.13	687.12	353.07	Y(+541.06)				8
2	198.12	930.24	912.23	465.62	L(+112.03)	925.50	907.49	463.25	7
3	44.05	1001.28	983.27	501.14	A	700.38	682.37	350.69	6
4	60.04	1088.31	1070.30	544.66	S	629.35	611.34	315.18	5
5	87.06	1202.36	1184.35	601.68	N	542.32	524.31	271.66	4
6	129.10	1358.46	1340.45	679.73	R	428.27	410.26	214.64	3
7	70.07	1455.51	1437.50	728.25	P	272.17	254.16	136.59	2
8	129.11				R	175.12	157.11	88.06	1

19)

Name	Uniprot ID	Sequence	RNA PTMs	M/Z	-lgP	z	start	end
Rbp2	X2JC79	R.YNNFNR(+151.05)(+212.01).H	G; rest	595.7231	13.35	2	252	257

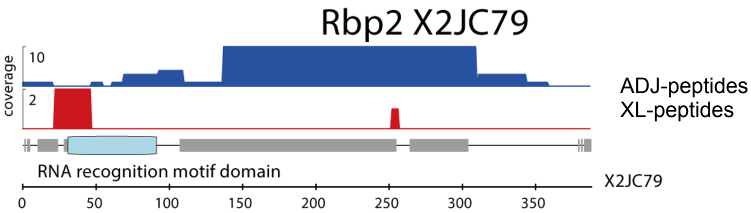

78) X2JC79_YNNFNR(+151.05)(+212.01)_595.72314_2

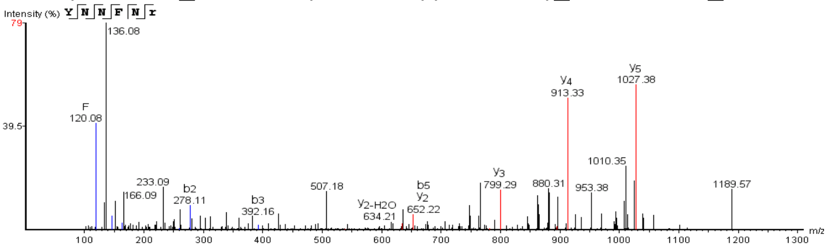

#	Immonium	b	b-H2O	b (2+)	Seq	y	y-H2O	y (2+)	#
1	136.08	164.06	146.06	82.54	Y				6
2	87.06	278.11	260.10	139.56	N	1027.38	1009.36	514.19	5
3	87.06	392.16	374.15	196.58	N	913.33	895.32	457.17	4
4	120.08	539.23	521.21	270.11	F	799.29	781.28	400.14	3
5	87.06	653.29	635.26	327.13	N	652.22	634.21	326.61	2
6	492.17				R(+363.06)	538.20	520.17	269.59	1

20)

Name	Uniprot ID	Sequence	RNA PTMs	M/Z	-lgP	z	start	end
Rm62	P19109	R.F(+112.03)(+212.01)GGGGGFGDR.R	U; rest	625.7272	28.46	2	209	218

Rm62

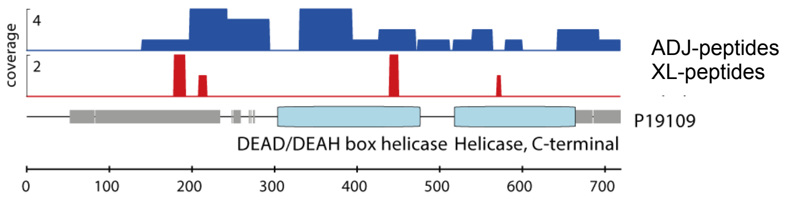

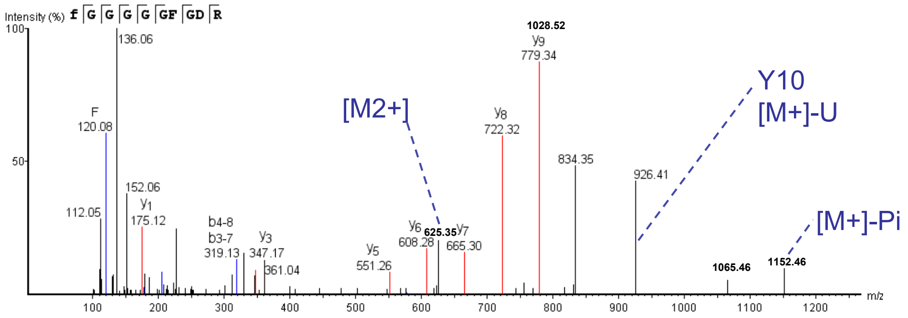

#	Immonium	b	b-H2O	b (2+)	Seq	y	y-H2O	y (2+)	#
1	444.12	472.11	454.10	236.56	F(+324.04)				10
2	30.03	529.13	511.12	265.07	G	779.34	761.33	390.17	9
3	30.03	586.16	568.14	293.58	G	722.32	704.31	361.66	8
4	30.03	643.18	625.17	322.09	G	665.30	647.29	333.15	7
5	30.03	700.20	682.19	350.60	G	608.28	590.27	304.64	6
6	30.03	757.22	739.21	379.11	G	551.26	533.25	276.13	5
7	120.08	904.29	886.28	452.64	F	494.24	476.23	247.62	4
8	30.03	961.31	943.30	481.15	G	347.17	329.16	174.08	3
9	88.04	1076.34	1058.33	538.67	D	290.15	272.14	145.57	2
10	129.11				R	175.12	157.11	88.06	1

21)

Name	Uniprot ID	Sequence	RNA PTMs	M/Z	-lgP	z	start	end
Rm62	P19109	R.F(+212.01)G(+112.03)GGGGGGDYHGIR.N	U; rest	815.8165	16.28	2	179	192

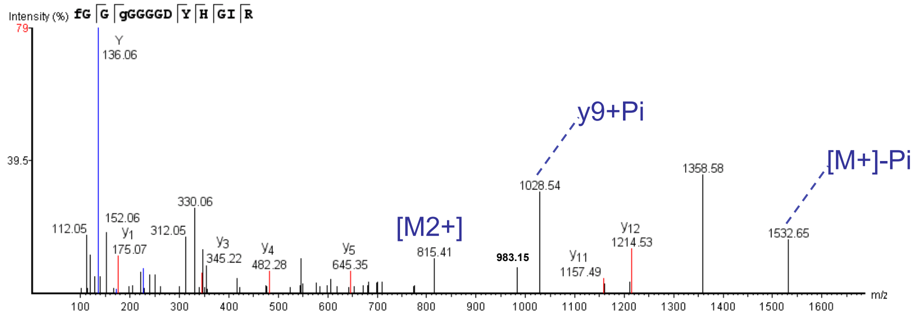

#	Immonium	b	b-H2O	b (2+)	Seq	y	y-H2O	y (2+)	#
1	332.09	360.08	342.07	180.54	F(+212.01)				14
2	30.03	417.11	399.10	209.05	G	1271.55	1253.54	636.28	13
3	30.03	474.13	456.12	237.56	G	1214.53	1196.52	607.76	12
4	142.06	643.18	625.17	322.09	G(+112.03)	1157.49	1139.50	579.25	11
5	30.03	700.20	682.19	350.60	G	988.46	970.45	494.73	10
6	30.03	757.22	739.21	379.11	G	931.44	913.43	466.22	9
7	30.03	814.24	796.23	407.62	G	874.42	856.41	437.71	8
8	30.03	871.26	853.25	436.13	G	817.40	799.38	409.20	7
9	88.04	986.29	968.28	493.64	D	760.37	742.36	380.69	6
10	136.06	1149.35	1131.34	575.18	Y	645.35	627.34	323.17	5
11	110.07	1286.41	1268.40	643.71	H	482.28	464.27	241.64	4
12	30.03	1343.43	1325.42	672.22	G	345.22	327.21	173.11	3
13	86.10	1456.52	1438.51	728.76	I	288.20	270.19	144.60	2
14	129.11				R	175.12	157.11	88.06	1

22)

Name	Uniprot ID	Sequence	RNA PTMs	M/Z	-lgP	z	start	end
tho2	E2QCS8	R.G(+111.04)GEERIR.H	C	464.2444	12.66	2	1452	1458

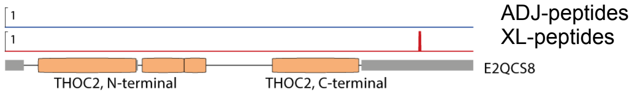

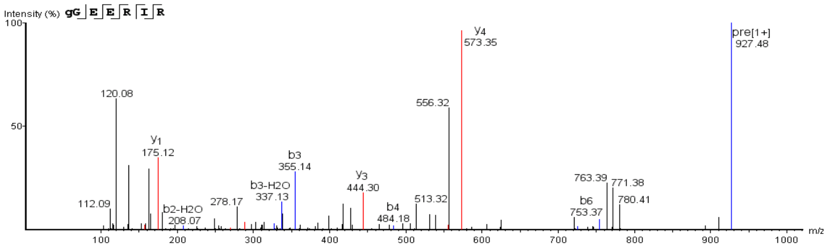

#	Immonium	b	b-H2O	b (2+)	Seq	y	y-H2O	y (2+)	#
1	141.08	169.07	151.06	85.04	G(+111.04)				7
2	30.03	226.09	208.07	113.55	G	759.41	741.40	380.21	6
3	102.06	355.14	337.13	178.07	E	702.39	684.38	351.69	5
4	102.06	484.18	466.17	242.59	E	573.35	555.34	287.17	4
5	129.11	640.28	622.27	320.64	R	444.30	426.29	222.65	3
6	86.10	753.37	735.35	377.18	I	288.20	270.19	144.60	2
7	129.11				R	175.12	157.11	88.06	1

23)

Name	Uniprot ID	Sequence	RNA PTMs	M/Z	-lgP	z	start	end
yps	Q95RE4	R.G(+112.03)(+212.01)LGGGDGSAEPGVHDQNPEGLQR.G	U; rest	857.6973	25.23	3	243	265

yps

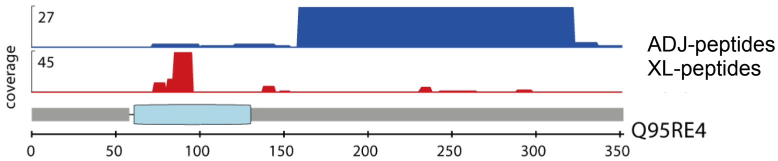

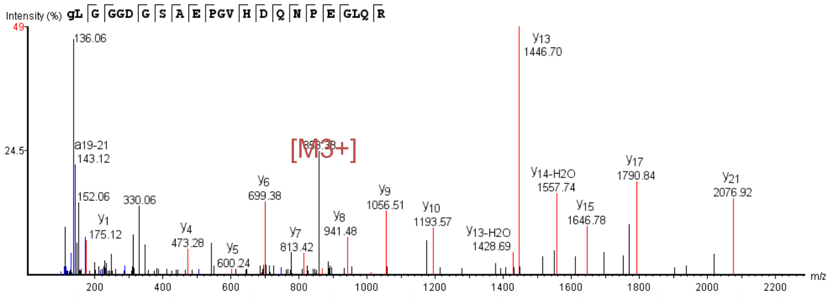

#	Immonium	b	b-H2O	b (2+)	Seq	y	y-H2O	y (2+)	#
1	354.07	382.07	364.05	191.53	G(+324.04)				23
2	86.10	495.15	477.14	248.07	L	2190.02	2172.01	1095.51	22
3	30.03	552.17	534.16	276.59	G	2076.92	2058.92	1038.97	21
4	30.03	609.19	591.18	305.10	G	2019.91	2001.90	1010.45	20
5	30.03	666.21	648.20	333.61	G	1962.89	1944.88	981.94	19
6	88.04	781.24	763.23	391.12	D	1905.87	1887.86	953.43	18
7	30.03	838.26	820.25	419.63	G	1790.84	1772.83	895.92	17
8	60.04	925.29	907.28	463.15	S	1733.82	1715.81	867.42	16
9	44.05	996.33	978.32	498.67	A	1646.78	1628.78	823.89	15
10	102.06	1125.37	1107.36	563.19	E	1575.75	1557.74	788.38	14
11	70.07	1222.43	1204.42	611.71	P	1446.70	1428.69	723.85	13
12	30.03	1279.45	1261.44	640.22	G	1349.66	1331.65	675.33	12
13	72.08	1378.52	1360.51	689.76	V	1292.63	1274.62	646.82	11
14	110.07	1515.58	1497.56	758.29	H	1193.57	1175.56	597.28	10
15	88.04	1630.60	1612.59	815.80	D	1056.51	1038.50	528.75	9
16	101.07	1758.66	1740.65	879.83	Q	941.48	923.47	471.24	8
17	87.06	1872.70	1854.69	936.85	N	813.42	795.41	407.21	7
18	70.07	1969.76	1951.75	985.38	P	699.38	681.37	350.19	6
19	102.06	2098.80	2080.79	1049.90	E	602.32	584.32	301.66	5
20	30.03	2155.82	2137.81	1078.41	G	473.28	455.27	237.14	4
21	86.10	2268.90	2250.89	1134.95	L	416.26	398.25	208.63	3
22	101.07	2396.96	2378.95	1198.98	Q	303.18	285.17	152.09	2
23	129.11				R	175.12	157.11	88.06	1

24)

Name	Uniprot ID	Sequence	RNA PTMs	M/Z	-lgP	z	start	end
yps	Q95RE4	R.G(+112.03)(+517.05)SQFAADK.R	C+rest; U	726.7407	18.35	2	138	145

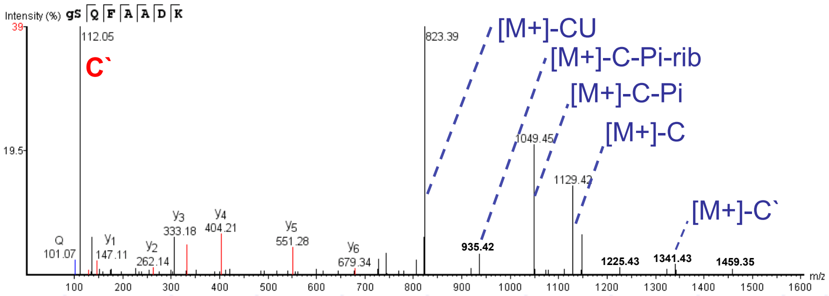

#	Immonium	b	b-H2O	b (2+)	Seq	y	y-H2O	y (2+)	#
1	659.11	687.11	669.10	344.05	G(+629.08)				8
2	60.04	774.14	756.13	387.57	S	766.37	748.36	383.69	7
3	101.07	902.20	884.19	451.60	Q	679.34	661.33	340.17	6
4	120.08	1049.27	1031.26	525.13	F	551.28	533.27	276.14	5
5	44.05	1120.30	1102.29	560.65	A	404.21	386.20	202.61	4
6	44.05	1191.34	1173.33	596.17	A	333.18	315.17	167.09	3
7	88.04	1306.37	1288.36	653.68	D	262.14	244.13	131.57	2
8	101.11				K	147.11	129.10	74.06	1

25)

Name	Uniprot ID	Sequence	RNA PTMs	M/Z	-lgP	z	start	end
larp	Q9VAW5	R.F(+112.03)(+212.01)SDDIADQR.R	U'; rest	695.7621	23.6	2	561	569

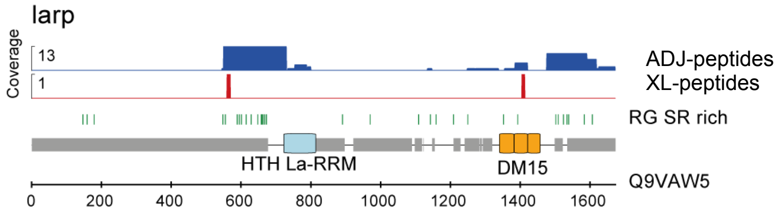

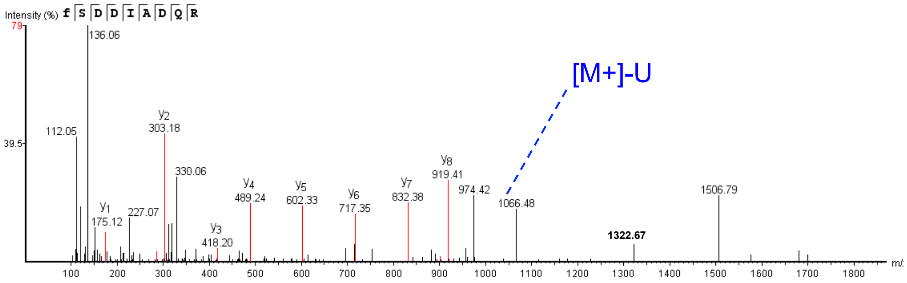

#	Immonium	b	b-H2O	b (2+)	Seq	y	y-H2O	y (2+)	#
1	444.12	472.11	454.10	236.56	F(+324.04)				9
2	60.04	559.14	541.13	280.07	S	919.41	901.40	460.21	8
3	88.04	674.17	656.16	337.59	D	832.38	814.37	416.69	7
4	88.04	789.20	771.19	395.10	D	717.35	699.34	359.18	6
5	86.10	902.28	884.27	451.64	I	602.33	584.32	301.66	5
6	44.05	973.32	955.31	487.16	A	489.24	471.23	245.12	4
7	88.04	1088.35	1070.34	544.67	D	418.20	400.19	209.60	3
8	101.07	1216.40	1198.39	608.70	Q	303.18	285.17	152.09	2
9	129.11				R	175.12	157.11	88.06	1

26)

Name	Uniprot ID	Sequence	RNA PTMs	M/Z	z	Position of XL
Sf3b1	Q9VPR5	MEN(+.98)IP(+151.05)R	G`	456.2123	2	Via base at P

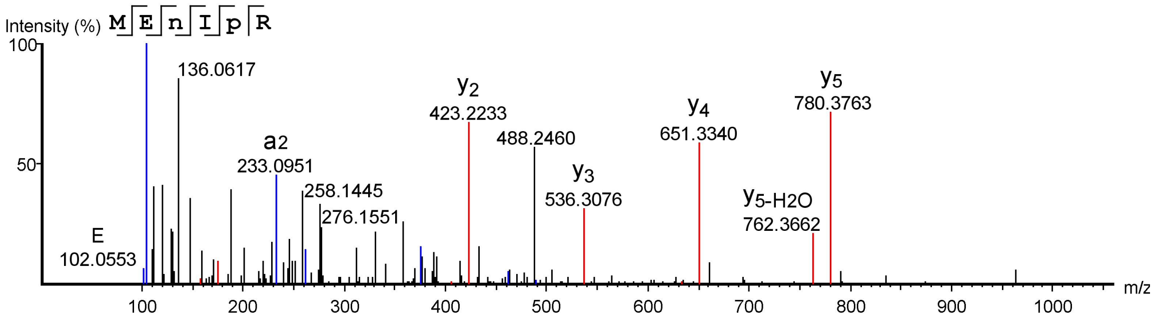

#	Immonium	b	a	b (2+)	a (2+)	Seq	y	y-H2O	y-NH3	y (2+)	#
1	104.0533	132.0483	104.0532	66.5242	52.5267	M					6
2	102.0553	261.0898	233.0951	131.0454	117.0479	E	780.3763	762.3662	763.3477	390.6873	5
3	88.0398	376.1171	348.1229	188.5589	174.5614	N(+.98)	651.3340	633.3235	634.3051	326.1660	4
4	86.0969	489.2005	461.2065	245.1010	231.1035	I	536.3076	518.2946	519.2781	268.6526	3
5	221.1150	737.3041	709.3091	369.1520	355.1545	P(+151.05)	423.2233	405.2134	406.1941	212.1105	2
6	129.1139					R	175.1188	157.0970	158.0919	88.0595	1

Human XL-peptides identifying Novel sites of interaction

27)

Name	Uniprot ID	Sequence	RNA PTMs	M/Z	z	Position of XL
HNRNPA1	P09651	N(+112.03)(+212.01)QGGYGGSSSSSSYGSGR	U	1009.87274	2	N.D.

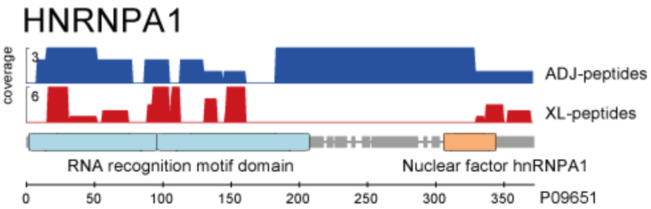

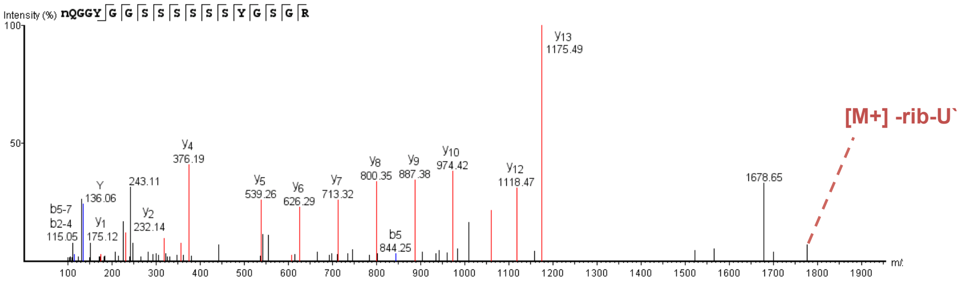

#	Immonium	b	b-H2O	b (2+)	Seq	y	y-H2O	y (2+)	#
1	411.09	439.09	421.08	220.04	N(+324.04)				18
2	101.07	567.15	549.13	284.07	Q	1580.66	1562.65	790.83	17
3	30.03	624.17	606.16	312.58	G	1452.60	1434.59	726.80	16
4	30.03	681.19	663.18	341.09	G	1395.58	1377.57	698.29	15
5	136.06	844.25	826.24	422.63	Y	1338.56	1320.55	669.78	14
6	30.03	901.27	883.26	451.14	G	1175.49	1157.48	588.25	13
7	30.03	958.29	940.28	479.65	G	1118.47	1100.46	559.74	12
8	60.04	1045.33	1027.32	523.16	S	1061.45	1043.44	531.22	11
9	60.04	1132.36	1114.35	566.68	S	974.42	956.41	487.71	10
10	60.04	1219.39	1201.38	610.20	S	887.38	869.37	444.19	9
11	60.04	1306.42	1288.41	653.71	S	800.35	782.34	400.68	8
12	60.04	1393.45	1375.44	697.23	S	713.32	695.31	357.15	7
13	60.04	1480.49	1462.48	740.74	S	626.29	608.28	313.64	6
14	136.08	1643.55	1625.54	822.27	Y	539.26	521.25	270.13	5
15	30.03	1700.57	1682.56	850.79	G	376.19	358.18	188.60	4
16	60.04	1787.60	1769.59	894.30	S	319.17	301.16	160.09	3
17	30.03	1844.62	1826.61	922.81	G	232.14	214.13	116.57	2
18	129.11				R	175.12	157.11	88.06	1

28)

Name	Uniprot ID	Sequence	RNA PTMs	M/Z	z	Position of XL
HNRNPA1	P09651	NQGGYGGSSSSSSYGSGR(+112.03)(+212.01)	U	1009.87085	2	N.D.

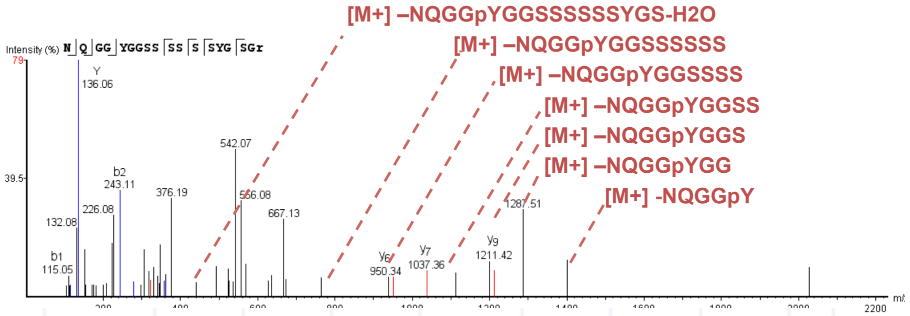

#	Immonium	b	b-H2O	b (2+)	Seq	y	y-H2O	y (2+)	#
1	87.06	115.05	97.04	58.03	N				18
2	101.07	243.11	225.10	122.05	Q	1904.69	1886.68	952.85	17
3	30.03	300.13	282.12	150.57	G	1776.63	1758.62	888.82	16
4	30.03	357.15	339.14	179.08	G	1719.61	1701.60	860.31	15
5	136.06	520.22	502.21	260.61	Y	1662.59	1644.58	831.80	14
6	30.03	577.24	559.23	289.12	G	1499.53	1481.52	750.26	13
7	30.03	634.26	616.25	317.63	G	1442.51	1424.50	721.75	12
8	60.04	721.29	703.28	361.15	S	1385.49	1367.47	693.24	11
9	60.04	808.32	790.31	404.66	S	1298.45	1280.44	649.73	10
10	60.04	895.35	877.34	448.18	S	1211.42	1193.41	606.21	9
11	60.04	982.39	964.38	491.69	S	1124.39	1106.38	562.69	8
12	60.04	1069.42	1051.41	535.21	S	1037.36	1019.35	519.18	7
13	60.04	1156.45	1138.44	578.73	S	950.34	932.31	475.66	6
14	136.08	1319.51	1301.50	660.26	Y	863.29	845.28	432.15	5
15	30.03	1376.54	1358.52	688.77	G	700.23	682.22	350.61	4
16	60.04	1463.57	1445.56	732.28	S	643.21	625.20	322.11	3
17	30.03	1520.59	1502.58	760.79	G	556.18	538.17	278.59	2
18	453.15				R(+324.04)	499.15	481.14	250.08	1

29)

Name	Uniprot ID	Sequence	RNA PTMs	M/Z	z	Position of XL
ALYREF	P09651	S(+212.01)SGPY(+112.03)GGGGQYFAK	U	850.3356	2	Via base at Y

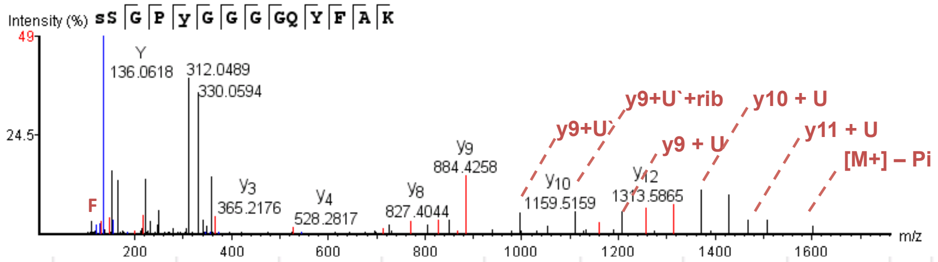

#	Immonium	b	b-H2O	b (2+)	Seq	y	y-H2O	y (2+)	#
1	272.0534	300.0484	282.0379	150.5242	S(+212.01)				14
2	60.0449	387.0804	369.0699	194.0402	S	1400.6229	1382.6124	700.8115	13
3	30.0343	444.1019	426.0914	222.5510	G	1313.5865	1295.5803	657.2954	12
4	70.0656	541.1547	523.1441	271.0773	P	1256.5635	1238.5590	628.7847	11
5	248.1034	816.2453	798.2347	408.6227	Y(+112.03)	1159.5159	1141.5061	580.2583	10
6	30.0343	873.2667	855.2562	437.1334	G	884.4258	866.4174	442.7130	9
7	30.0343	930.2882	912.2776	465.6441	G	827.4044	809.3940	414.2023	8
8	30.0343	987.3097	969.2991	494.1548	G	770.3831	752.3726	385.6916	7
9	30.0343	1044.3312	1026.3207	522.6656	G	713.3618	695.3511	357.1808	6
10	101.0714	1172.3896	1154.3792	586.6948	Q	656.3402	638.3297	328.6701	5
11	136.0618	1335.4530	1317.4425	668.2265	Y	528.2817	510.2711	264.6408	4
12	120.0810	1482.5215	1464.5110	741.7607	F	365.2176	347.2077	183.1091	3
13	44.0499	1553.5586	1535.5481	777.2793	A	218.1497	200.1397	109.5750	2
14	101.1078				K	147.1128	129.1024	74.0564	1

30)

Name	Uniprot ID	Sequence	RNA PTMs	M/Z	z	Position of XL
HNRNPA2B1	P22626	G(+212.01)G(+112.03)NFGFGDSR	U	669.24286	2	N.D.

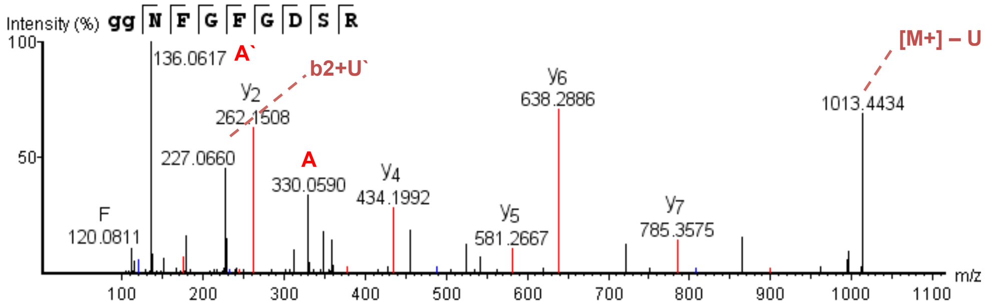

#	Immonium	b	b-H2O	b (2+)	Seq	y	y-H2O	y (2+)	#
1	242.0429	270.0379	252.0273	135.5189	G(+212.01)				10
2	142.0616	439.0866	421.0760	220.0433	G(+112.03)	1068.4493	1050.4388	534.7247	9
3	87.0558	553.1295	535.1190	277.0648	N	899.3923	881.3900	450.2003	8
4	120.0811	700.1979	682.1874	350.5990	F	785.3575	767.3470	393.1788	7
5	30.0343	757.2194	739.2089	379.1097	G	638.2886	620.2787	319.6446	6
6	120.0812	904.2878	886.2773	452.6439	F	581.2667	563.2572	291.1339	5
7	30.0343	961.3093	943.2987	481.1546	G	434.1992	416.1888	217.5997	4
8	88.0398	1076.3362	1058.3257	538.6681	D	377.1765	359.1673	189.0889	3
9	60.0449	1163.3683	1145.3578	582.1841	S	262.1508	244.1399	131.5755	2
10	129.1139				R	175.1188	157.1084	88.0595	1

31)

Name	Uniprot ID	Sequence	RNA PTMs	M/Z	z	Position of XL
HNRNPCL1	O60812	S(+112.03)(+212.01)GFNSK	U	482.17645	2	N.D.

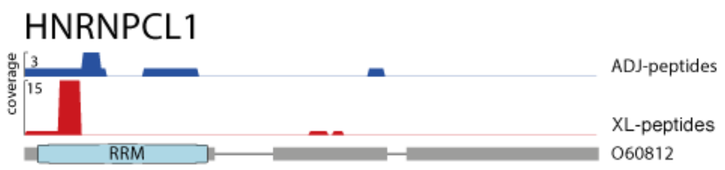

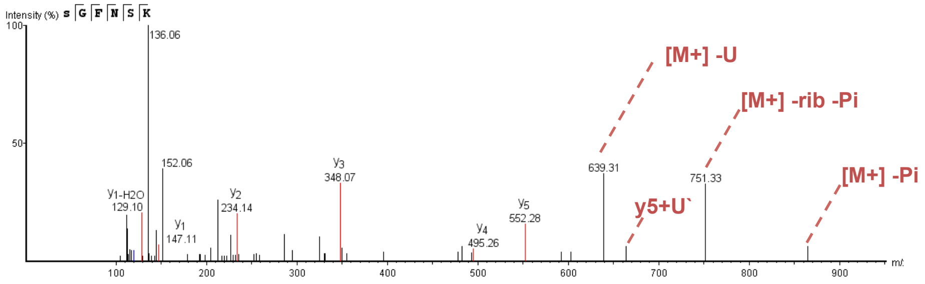

#	Immonium	b	b-H2O	b (2+)	Seq	y	y-H2O	y (2+)	#
1	384.08	412.08	394.07	206.54	S(+324.04)				6
2	30.03	469.10	451.09	235.05	G	552.28	534.27	276.64	5
3	120.08	616.17	598.16	308.58	F	495.26	477.25	248.13	4
4	87.06	730.21	712.20	365.60	N	348.19	330.18	174.59	3
5	60.04	817.24	799.23	409.12	S	234.14	216.13	117.57	2
6	101.11				K	147.11	129.10	74.06	1

32)

Name	Uniprot ID	Sequence	RNA PTMs	M/Z	z	Position of XL
LRPPRC	P42704	D(+112.03)(+212.01)IQEESTFSSR	U	811.8136	2	N.D.

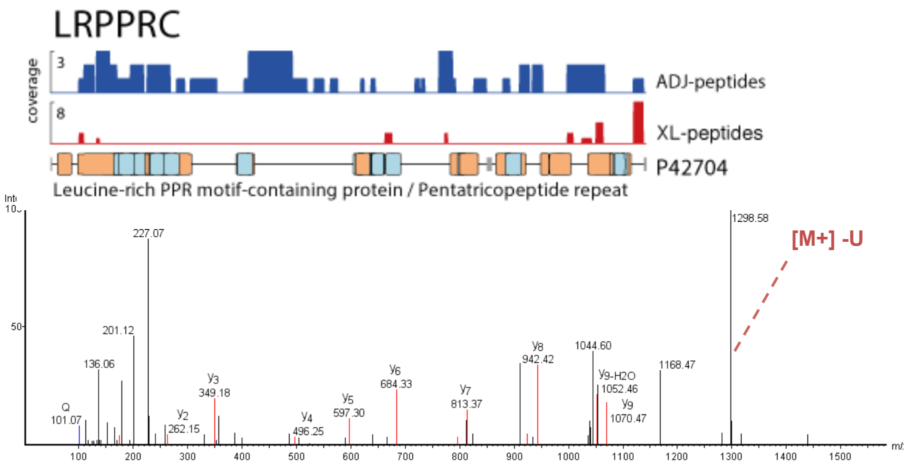

#	Immonium	b	b-H2O	b (2+)	Seq	y	y-H2O	y (2+)	#
1	412.08	440.07	422.06	220.54	D(+324.04)				11
2	86.10	553.15	535.14	277.08	I	1183.56	1165.55	592.28	10
3	101.07	681.21	663.20	341.11	Q	1070.47	1052.46	535.74	9
4	102.06	810.26	792.25	405.63	E	942.42	924.40	471.71	8
5	102.06	939.30	921.29	470.15	E	813.37	795.36	407.19	7
6	60.04	1026.33	1008.32	513.67	S	684.33	666.32	342.67	6
7	74.06	1127.38	1109.37	564.19	T	597.30	579.29	299.15	5
8	120.08	1274.45	1256.44	637.72	F	496.25	478.24	248.63	4
9	60.04	1361.48	1343.47	681.24	S	349.18	331.17	175.09	3
10	60.04	1448.51	1430.50	724.76	S	262.15	244.14	131.58	2
11	129.11				R	175.12	157.11	88.06	1

33)

Name	Uniprot ID	Sequence	RNA PTMs	M/Z	z	Position of XL
LRPPRC	P42704	L(+194.00)QW(+112.03)FC(+57.02)DR	U-H2O	665.75024	2	Via base at W

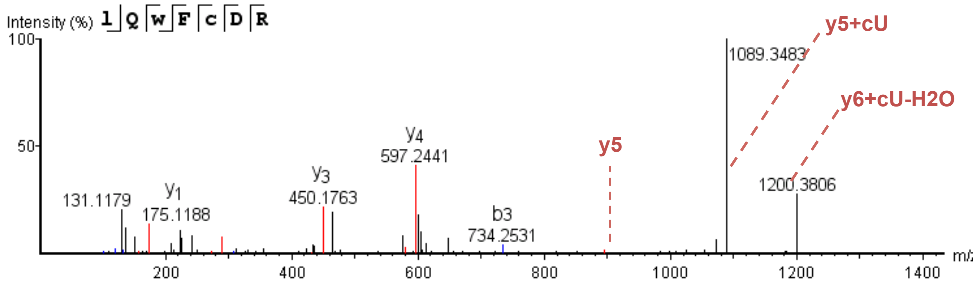

#	Immonium	b	b-H2O	b (2+)	Seq	y	y-H2O	y (2+)	#
1	280.0949	308.1068	290.0793	154.5450	L(+194.00)				7
2	101.0713	436.1485	418.1379	218.5742	Q	1023.4102	1005.3995	512.2051	6
3	271.1194	734.2531	716.2445	367.6275	W(+112....	895.3524	877.3409	448.1758	5
4	120.0808	881.3235	863.3129	441.1617	F	597.2441	579.2362	299.1225	4
5	133.0606	1041.3541	1023.3435	521.1771	C(+57.02)	450.1763	432.1668	225.5883	3
6	88.0398	1156.3811	1138.3706	578.6906	D	290.1453	272.1353	145.5729	2
7	129.1139				R	175.1188	157.1086	88.0595	1

34)

Name	Uniprot ID	Sequence	RNA PTMs	M/Z	z	Position of XL
MTHFSD	Q2M296	IGK(+151.05)GEGYADLEYAMMVSMGAVSK	G`	853.40204	3	unknown

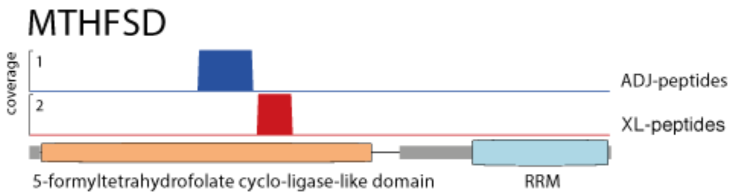

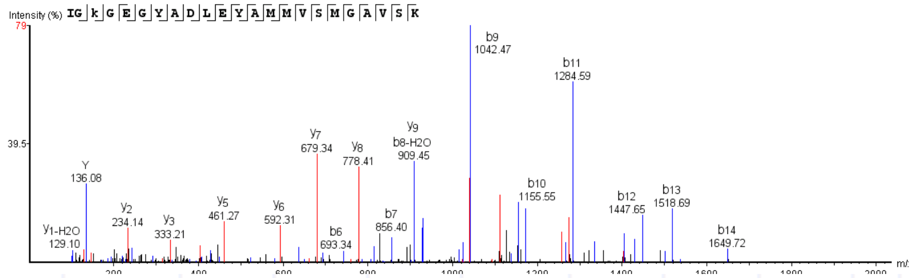

#	Immonium	b	b-H2O	b (2+)	Seq	y	y-H2O	y (2+)	#
1	86.10	114.09	96.08	57.55	I				23
2	30.03	171.11	153.10	86.06	G	2445.10	2427.09	1223.05	22
3	252.16	450.25	432.25	225.63	K(+151.05)	2388.08	2370.07	1194.54	21
4	30.03	507.28	489.27	254.14	G	2108.93	2090.92	1054.97	20
5	102.05	636.32	618.31	318.66	E	2051.91	2033.90	1026.45	19
6	30.03	693.34	675.33	347.17	G	1922.87	1904.86	961.93	18
7	136.08	856.40	838.40	428.70	Y	1865.85	1847.84	933.42	17
8	44.05	927.44	909.45	464.22	A	1702.78	1684.77	851.89	16
9	88.04	1042.47	1024.46	521.74	D	1631.75	1613.74	816.37	15
10	86.10	1155.55	1137.53	578.28	L	1516.72	1498.71	758.86	14
11	102.06	1284.59	1266.58	642.80	E	1403.63	1385.63	702.32	13
12	136.08	1447.65	1429.64	724.33	Y	1274.59	1256.59	637.80	12
13	44.05	1518.69	1500.68	759.85	A	1111.53	1093.52	556.27	11
14	104.05	1649.72	1631.73	825.37	M	1040.49	1022.48	520.75	10
15	104.05	1780.78	1762.77	890.89	M	909.45	891.44	455.23	9
16	72.08	1879.85	1861.84	940.42	V	778.41	760.40	389.71	8
17	60.04	1966.88	1948.87	983.94	S	679.34	661.33	340.17	7
18	104.05	2097.92	2079.91	1049.46	M	592.31	574.30	296.66	6
19	30.03	2154.94	2136.93	1077.97	G	461.27	443.26	231.14	5
20	44.05	2225.98	2207.97	1113.49	A	404.25	386.24	202.63	4
21	72.08	2325.05	2307.04	1163.02	V	333.21	315.20	167.11	3
22	60.04	2412.08	2394.07	1206.54	S	234.14	216.13	117.57	2
23	101.11				K	147.11	129.10	74.06	1

35)

Name	Uniprot ID	Sequence	RNA PTMs	M/Z	z	Position of XL
YBX1	H0Y449	N(+212.01)YQQNY(+112.03)QNSESGEK	U	1006.8802	2	Via base at Y

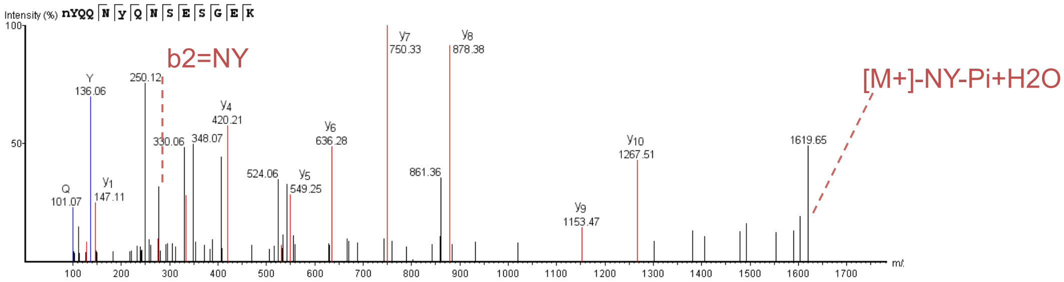

#	Immonium	b	b-H2O	b (2+)	Seq	y	y-H2O	y (2+)	#
1	299.06	327.06	309.05	164.03	N(+212.01)				14
2	136.06	490.12	472.11	245.56	Y	1686.70	1668.69	843.85	13
3	101.07	618.18	600.17	309.59	Q	1523.64	1505.63	762.32	12
4	101.07	746.24	728.23	373.62	Q	1395.58	1377.57	698.29	11
5	87.06	860.28	842.27	430.64	N	1267.51	1249.51	634.26	10
6	248.10	1135.37	1117.36	568.19	Y(+112.03)	1153.47	1135.47	577.24	9
7	101.07	1263.43	1245.42	632.22	Q	878.38	860.37	439.69	8
8	87.06	1377.47	1359.46	689.24	N	750.33	732.32	375.66	7
9	60.04	1464.51	1446.50	732.75	S	636.28	618.27	318.64	6
10	102.06	1593.55	1575.54	797.27	E	549.25	531.24	275.13	5
11	60.04	1680.58	1662.57	840.79	S	420.21	402.20	210.60	4
12	30.03	1737.60	1719.59	869.30	G	333.18	315.17	167.09	3
13	102.06	1866.65	1848.64	933.82	E	276.16	258.14	138.58	2
14	101.11				K	147.11	129.10	74.06	1

36)

Name	Uniprot ID	Sequence	RNA PTMs	M/Z	z	Position of XL
YBX3	P16989	Y(+212.01)(+112.03)AADR	U	460.16287	2	Via ribose at N-term

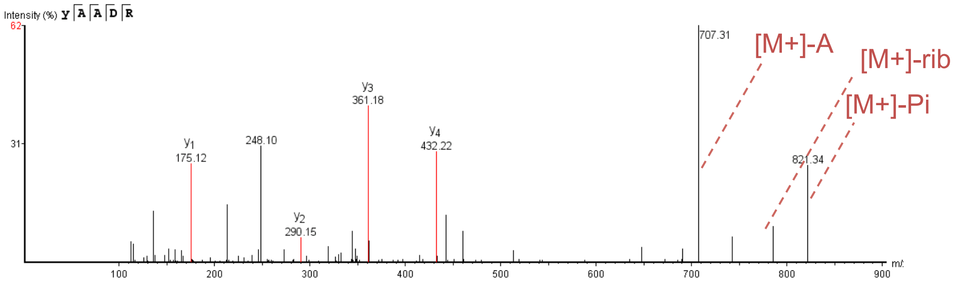

#	Immonium	b	b-H2O	b (2+)	Seq	y	y-H2O	y (2+)	#
1	460.11	488.11	470.10	244.55	Y(+324.04)				5
2	44.05	559.14	541.13	280.07	A	432.22	414.21	216.61	4
3	44.05	630.18	612.17	315.59	A	361.18	343.17	181.09	3
4	88.04	745.21	727.20	373.10	D	290.15	272.14	145.57	2
5	129.11				R	175.12	157.11	88.06	1

37)

Name	Uniprot ID	Sequence	RNA PTMs	M/Z	z	Position of XL
PRKDC	P78527	C(+112.03)GAALAGHQLIR	U`	661.3421	2	Via base at C

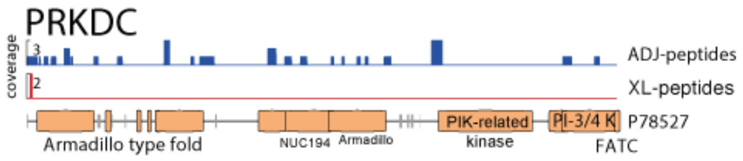

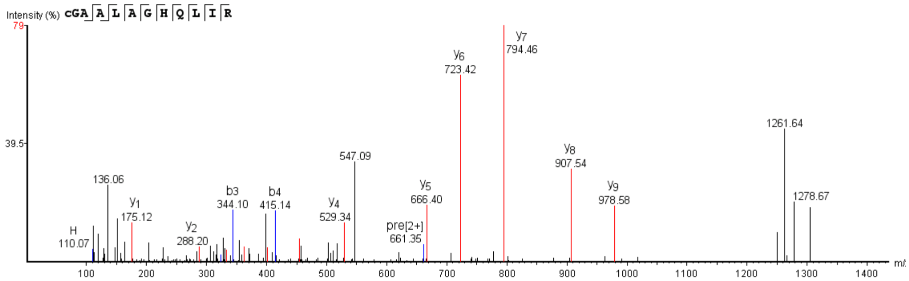

#	Immonium	b	b-H2O	b (2+)	Seq	y	y-H2O	y (2+)	#
1	188.05	216.04	198.03	108.52	C(+112.03)				12
2	30.03	273.07	255.06	137.03	G	1106.64	1088.63	553.82	11
3	44.05	344.10	326.09	172.55	A	1049.62	1031.61	525.31	10
4	44.05	415.14	397.13	208.07	A	978.58	960.57	489.79	9
5	86.10	528.22	510.21	264.61	L	907.54	889.54	454.28	8
6	44.05	599.26	581.25	300.13	A	794.46	776.45	397.73	7
7	30.03	656.28	638.27	328.64	G	723.42	705.42	362.21	6
8	110.07	793.34	775.33	397.17	H	666.40	648.39	333.70	5
9	101.07	921.40	903.39	461.20	Q	529.34	511.34	265.17	4
10	86.10	1034.48	1016.47	517.74	L	401.29	383.28	201.14	3
11	86.10	1147.57	1129.56	574.28	I	288.20	270.19	144.60	2
12	129.11				R	175.12	157.11	88.06	1
