# Supplementary material for: CAPRI enables comparison of evolutionarily conserved RNA interacting regions
Source: Nat Commun. 2019 Jun 18;10:2682. doi: 10.1038/s41467-019-10585-3 (PMC6581911; doi:10.1038/s41467-019-10585-3)
Supplement: Supplementary file 17 — Reporting Summary [file 41467_2019_10585_MOESM17_ESM.pdf]

naturereseach

Corresponding author(s): Asifa Akhtar, Gerhard Mittler

Last updated by author(s): May 9, 2019

Reporting Summary

Nature Research wishes to improve the reproducibility of the work that we publish. This form provides structure for consistency and transparency in reporting. For further information on Nature Research policies, see Authors & Referees and the Editorial Policy Checklist.

Statistics

For all statistical analyses, confirm that the following items are present in the figure legend, table legend, main text, or Methods section.

n/a	Confirmed	
☐	☒	The exact sample size (*n*) for each experimental group/condition, given as a discrete number and unit of measurement
☐	☒	A statement on whether measurements were taken from distinct samples or whether the same sample was measured repeatedly
☐	☒	The statistical test(s) used AND whether they are one- or two-sided<br>*Only common tests should be described solely by name; describe more complex techniques in the Methods section.*
☒	☐	A description of all covariates tested
☐	☒	A description of any assumptions or corrections, such as tests of normality and adjustment for multiple comparisons
☐	☒	A full description of the statistical parameters including central tendency (e.g. means) or other basic estimates (e.g. regression coefficient) AND variation (e.g. standard deviation) or associated estimates of uncertainty (e.g. confidence intervals)
☐	☒	For null hypothesis testing, the test statistic (e.g. *F*, *t*, *r*) with confidence intervals, effect sizes, degrees of freedom and *P* value noted<br>*Give P values as exact values whenever suitable.*
☒	☐	For Bayesian analysis, information on the choice of priors and Markov chain Monte Carlo settings
☒	☐	For hierarchical and complex designs, identification of the appropriate level for tests and full reporting of outcomes
☒	☐	Estimates of effect sizes (e.g. Cohen's *d*, Pearson's *r*), indicating how they were calculated

Our web collection on statistics for biologists contains articles on many of the points above.

Software and code

Policy information about availability of computer code

Data collection	Mass spectrometric data collection was performed in Q Exactive mass spectrometer using Xcalibur software.
Data analysis	Mass Spectrometric spectra were analyzed by MaxQuant and PEAKS8.0<br>Protein structure rendering was done by UCSF Chimera, MacPyMol.<br>Custom Code was written using Python 2.7, Jupyter Notebook, PANDAS, SQLite3, matplotLib. All codes are available on reasonable request.

For manuscripts utilizing custom algorithms or software that are central to the research but not yet described in published literature, software must be made available to editors/reviewers. We strongly encourage code deposition in a community repository (e.g. GitHub). See the Nature Research guidelines for submitting code & software for further information.

Data

Policy information about availability of data

All manuscripts must include a data availability statement. This statement should provide the following information, where applicable:

- Accession codes, unique identifiers, or web links for publicly available datasets
- A list of figures that have associated raw data
- A description of any restrictions on data availability

All data including raw and analysed data is made available via link in the manuscript. Upon publication the raw data will be made accessible via PRIDE

Field-specific reporting

Please select the one below that is the best fit for your research. If you are not sure, read the appropriate sections before making your selection.

☒ Life sciences ☐ Behavioural & social sciences ☐ Ecological, evolutionary & environmental sciences

For a reference copy of the document with all sections, see nature.com/documents/nr-reporting-summary-flat.pdf

Life sciences study design

All studies must disclose on these points even when the disclosure is negative.

Sample size	A minimum of three biological replicates were used for all mass spectrometric analysis.
Data exclusions	No data was excluded.
Replication	Independent biological replicates were used.
Randomization	No Randomisation was used in the study
Blinding	Not Applicable

Reporting for specific materials, systems and methods

We require information from authors about some types of materials, experimental systems and methods used in many studies. Here, indicate whether each material, system or method listed is relevant to your study. If you are not sure if a list item applies to your research, read the appropriate section before selecting a response.

Materials & experimental systems

n/a	Involved in the study	
☐	☒	Antibodies
☐	☒	Eukaryotic cell lines
☒	☐	Palaeontology
☒	☐	Animals and other organisms
☒	☐	Human research participants
☒	☐	Clinical data

Methods

n/a	Involved in the study	
☒	☐	ChIP-seq
☒	☐	Flow cytometry
☒	☐	MRI-based neuroimaging

Antibodies

Antibodies used	We utilized the following primary antibodies directed against Drosophila MLE (custom made Mendjan et al., 2006), glorund (DSHB 5B7_C), Squid (DSHB 2G9-c), Rump (DSHB 5G4), Histone H3 (Active Motif 39763) and Beta Actin (Santa Cruz (I-19) sc-1616) as well as antibodies recognizing human DHX9 (Abcam ab183731), HNRNPM (Santa Cruz sc-20002), EIF-4A1 (Abcam EPR14506/Ab185946), KHDRBS1 (Sigma S9575), BETA ACTIN (Santa Cruz (I-19) sc- 1616), histone H3 (Active Motif 39763), H4 (Millipore 05-858), OXPHOS Rodent WB Antibody Cocktail (Abcam ab110413), SDHA(Invitrogen 459200), TOMM20 (Santa Cruz sc-11415), XRCC5 (Invitrogen MA5-15873), MSH6 (Cell Signalling 5424P), GAPDH (Bethyl A300-641A), HUR (3A2) (Santa Cruz 5261) and FLAG HRP (Sigma A8592)
Validation	Drosophila MLE (custom made Mendjan et al., 2006)<br>Following commercial antibodies have been validated by the respective manufacturers:<br>glorund (DSHB 5B7_C), Squid (DSHB 2G9-c), Rump (DSHB 5G4), Histone H3 (Active Motif 39763) and Beta Actin (Santa Cruz (I-19) sc-1616) as well as antibodies recognizing human DHX9 (Abcam ab183731), HNRNPM (Santa Cruz sc-20002), EIF-4A1 (Abcam EPR14506/Ab185946), KHDRBS1 (Sigma S9575), BETA ACTIN (Santa Cruz (I-19) sc- 1616), histone H3 (Active Motif 39763), H4 (Millipore 05-858),OXPHOS Rodent WB Antibody Cocktail (Abcam ab110413), SDHA(Invitrogen 459200), TOMM20 (Santa Cruz sc-11415), XRCC5 (Invitrogen MA5-15873), MSH6 (Cell Signalling 5424P), GAPDH (Bethyl A300-641A), HUR (3A2) (Santa Cruz 5261) and FLAG HRP (Sigma A8592)

Eukaryotic cell lines

Policy information about cell lines

Cell line source(s)	S2 DRSC cells from DGRC (Stock #181 from Drosophila Genomics Resource Centre), Flp-In 293 T-REx (ThermoFisher R78007)
Authentication	None of the cell lines were authenticated as they were procured from established sources

Mycoplasma contamination	Mycoplasma contamination test was not performed.
Commonly misidentified lines<br>(See ICLAC register)	*Name any commonly misidentified cell lines used in the study and provide a rationale for their use.*
